# Inhibition of Ezh2 redistributes bivalent domains within transcriptional regulators associated with WNT and Hedgehog pathways in osteoblasts

**DOI:** 10.1016/j.jbc.2023.105155

**Published:** 2023-08-11

**Authors:** Margarita E. Carrasco, Roman Thaler, Gino Nardocci, Amel Dudakovic, Andre J. van Wijnen

**Affiliations:** 1 Department of Orthopedic Surgery, Mayo Clinic, Rochester, Minnesota, USA; 2 Program in Molecular Biology and Bioinformatics, Faculty of Medicine, Center for Biomedical Research and Innovation (CIIB), Universidad de los Andes, Santiago, Chile; 3 IMPACT, Center of Interventional Medicine for Precision and Advanced Cellular Therapy, Santiago, Chile; 4 Department of Biochemistry and Molecular Biology, Mayo Clinic, Rochester, Minnesota, USA; 5 Department of Biochemistry, University of Vermont, Burlington, Vermont, USA

**Keywords:** enhancer of zeste homolog, Ezh2, EPZ6438, epigenetics, histone methylation, osteoblast, osteogenesis, bivalent domains

## Abstract

Bivalent epigenomic regulatory domains containing both activating histone 3 lysine 4 (H3K4me3) and repressive lysine 27 (H3K27me3) trimethylation are associated with key developmental genes. These bivalent domains repress transcription in the absence of differentiation signals but maintain regulatory genes in a poised state to allow for timely activation. Previous studies demonstrated that enhancer of zeste homolog 2 (Ezh2), a histone 3 lysine 27 (H3K27) methyltransferase, suppresses osteogenic differentiation and that inhibition of Ezh2 enhances commitment of osteoblast progenitors *in vitro* and bone formation *in vivo*. Here, we examined the mechanistic effects of Tazemetostat (EPZ6438), an Food and Drug Administration approved Ezh2 inhibitor for epithelioid sarcoma treatment, because this drug could potentially be repurposed to stimulate osteogenesis for clinical indications. We find that Tazemetostat reduces H3K27me3 marks in bivalent domains in enhancers required for bone formation and stimulates maturation of MC3T3 preosteoblasts. Furthermore, Tazemetostat activates bivalent genes associated with the Wingless/integrated (WNT), adenylyl cyclase (cAMP), and Hedgehog (Hh) signaling pathways based on transcriptomic (RNA-seq) and epigenomic (chromatin immunoprecipitation [ChIP]-seq) data. Functional analyses using selective pathway inhibitors and silencing RNAs demonstrate that the WNT and Hh pathways modulate osteogenic differentiation after Ezh2 inhibition. Strikingly, we show that loss of the Hh-responsive transcriptional regulator Gli1, but not Gli2, synergizes with Tazemetostat to accelerate osteoblast differentiation. These studies establish epigenetic cooperativity of Ezh2, Hh-Gli1 signaling, and bivalent regulatory genes in suppressing osteogenesis. Our findings may have important translational ramifications for anabolic applications requiring bone mass accrual and/or reversal of bone loss.

In bone regenerative medicine, a critical step in the treatment of bone defects and skeletal disorders is the osteogenic differentiation efficiency of mesenchymal stromal/stem cells (MSCs) and precommitted osteoblasts (1, 2). Because transcription of bone-related genes is regulated by the degree of chromatin compaction, it is necessary to understand how chromatin-related epigenetic mechanisms control osteogenesis (3, 4). Bone cell differentiation is controlled by (hydroxy-)methylation of DNA, ATP-dependent chromatin remodeling, and nucleosome deposition, as well as removal of posttranslational modifications on histone and non-histone proteins (3, 5, 6). Chromatin organization in osteogenic cells is controlled by covalent posttranslational modifications of the four core histones (*i.e.*, H2A, H2B, H3, and H4), including methylation, acetylation, phosphorylation, and ubiquitination (7).

One important chromatin modifier during osteogenesis is the polycomb repressor complex 2 (PRC2), which contains three core proteins (Suz12, Eed, and RbAsp48) and one of the two methyltransferases (Ezh1 or Ezh2) (8, 9). PRC2 is responsible for catalyzing the trimethylation of histone H3 at lysine 27 (H3K27me3), which then serves as an epigenetic signal for chromatin condensation and transcriptional repression (10, 11). In humans, heterozygous mutations in EZH2 cause Weaver syndrome, which is a disorder characterized by overgrowth, macrocephaly, advanced bone age, variable intellectual disability, and distinctive facial features (12). Similarly, heterozygous mutations in EED cause Cohen–Gibson syndrome (12, 13), which is very similar to Weaver syndrome. Furthermore, rare coding variants in SUZ12 have been shown to resemble Weaver syndrome and Cohen–Gibson syndrome (14).

The key roles of EZH2 in stem cell selfrenewal (15) and the oncogenic effects EZH2 mutations and overexpression have rendered this protein attractive as a drug target for cancer therapy (16). EZH2 has a SET domain that catalyzes the monomethylation, dimethylation, and trimethylation of H3K27 from the universal methyl donor SAM (17). One class of inhibitors that affects EZH2 activity targets the regeneration of SAM *via* the folate/methionine cycles, which provide a continuous source of methyl groups. EZH2 activity results in methyl donation, which converts SAM to SAH. SAH is then hydrolyzed by with SAH hydrolase to generate homocysteine, which is then converted to cysteine *via trans*-sulfuration into methionine. Drugs that target the folate cycle (*e.g.*, the SAH hydrolase inhibitor 3-Deazaneplanocin; DZNep) have been considered for bone stimulation (18, 19) but such drugs have mechanistic effects beyond EZH2, which is not desirable for future bone anabolic clinical applications.

Potent inhibitors have been developed that directly inhibit the enzymatic activity of EZH2 (*e.g.*, GSK126, UNC1999) (8, 20, 21), many of which we have systematically tested *in vitro* and/or *in vivo* (22, 23). Because EZH2 only possesses methyltransferase activity when incorporated into PRC2, a second class of inhibitors disrupts the EZH2––EED complex by binding to an α-helix of EZH2 the (*e.g.*, EPZ6438, Tazemetostat, Tazverik). Tazemetostat is a small molecule EZH2 inhibitor that is clinically approved for the treatment of epithelioid sarcoma (20, 21). Because extensive safety data are available for Tazemetostat, we are compelled to test its osteogenic activity and assess whether this inhibitor could be repurposed for short-term bone stimulatory applications.

Ezh2 is one of several enzymes that controls normal skeletal development (24). This epigenetic enzyme supports selfrenewal of MSCs, while blocking osteogenic commitment of progenitor cells. siRNA-mediated depletion and/or small molecule inhibition of Ezh2 enhance osteogenic differentiation of MSCs and precommitted osteoblasts *in vitro*, while inhibition of Ezh2 stimulates bone formation and mitigates bone loss associated with estrogen depletion *in vivo* (18, 22, 23, 24, 25, 26, 27, 28, 29, 30, 31, 32, 33, 34, 35, 36, 37, 38, 39, 40, 41). Mechanistically, the pro-osteogenic effects resulting from Ezh2 inactivation arise from the activation of key osteogenic pathways and enhanced expression of genes associated with bone formation (22, 24, 25, 27). It remains unresolved how exactly Ezh2 controls osteogenesis as this process is a highly regulated multistage program of mesenchymal cell fate determination, lineage commitment, and differentiation that generates committed osteoprogenitors, mature osteoblasts, and terminally differentiated osteocytes embedded in mineral (i.e., matrix formation and mineralization) (42).

Bivalent gene regulatory domains allow rapid activation of key tissue–specific genes by switching from a silenced state (H3K4me3-positive and H3K27me3-positive) to a transcriptionally active state (H3K4me3-positive and H3K27me3-negative) (7, 43). Hence, Ezh2 inhibition, which will cause a rapid loss of the H3K27me3 marks in gene regulatory regions near H3K4me3 marks, is predicted to stimulate expression of genes with bivalent chromatin. Remarkably, the contribution of bivalent domains to osteogenesis has not yet been elucidated. In this study, we investigated how H3K27me3 reduction upon Ezh2 inhibition controls osteogenesis *via* genes regulated by bivalent chromatin.

## Results

### Tazemetostat/EPZ6438 rapidly blocks H3K27me3 levels and enhances osteogenic differentiation

**Figure 1:**
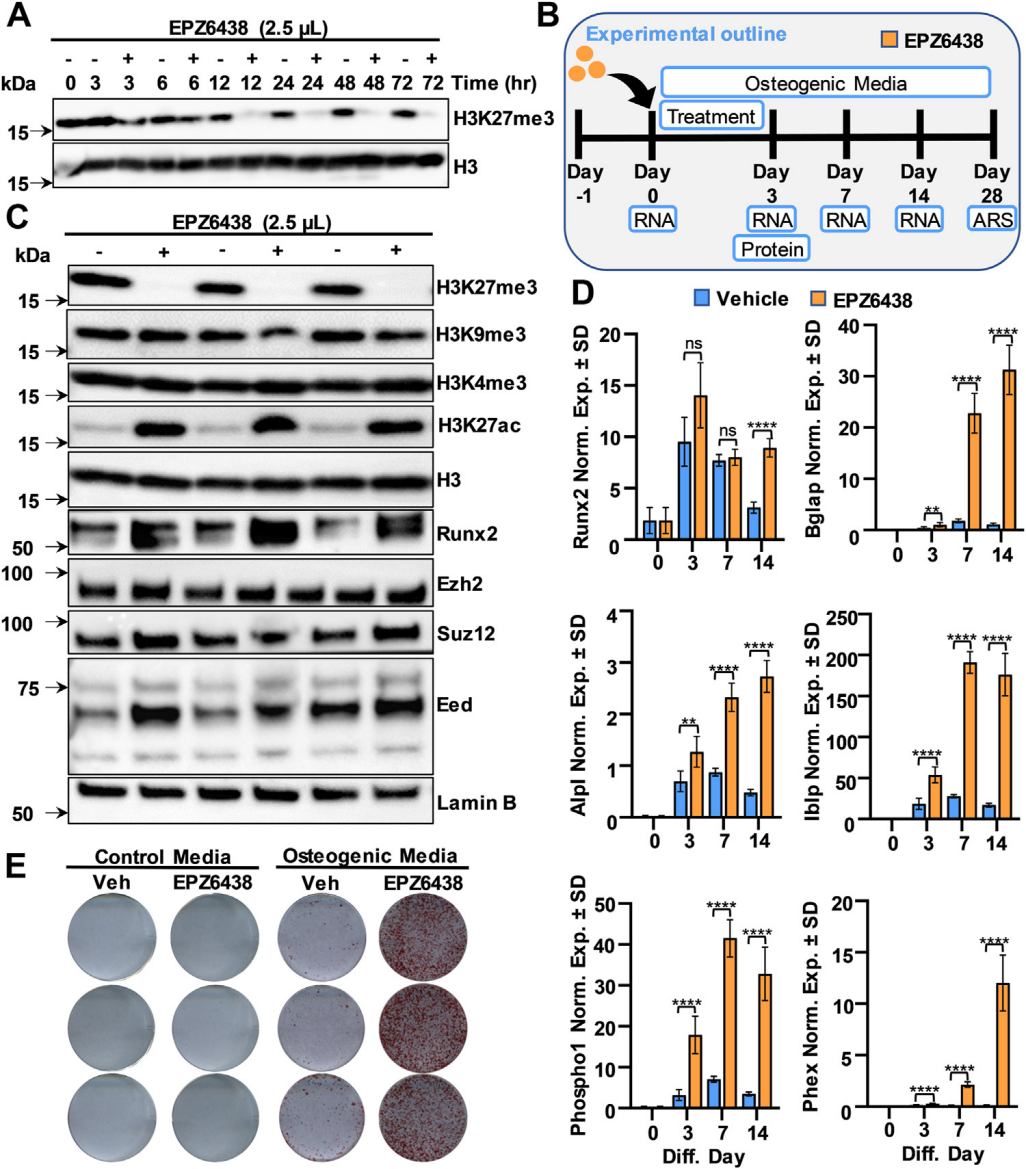
**Ezh2 inhibition by EPZ6438 enhances osteogenic differentiation of MC3T3 cells.** Cells were treated with 2.5 μM EPZ6438 1 day after plating and harvested at the specified time points and protein lysates assessed by Western blotting (*A*). Diagram of the experimental protocol for treatment of MC3T3 with vehicle (Veh) or 2.5 μM EPZ6438 shown in panels *C*–*E* (*B*). Western blotting analysis on day 3 (*C*). RT-qPCR analysis for bone-related markers relative to Gapdh (set at 100) at indicated time points (*D*). Alizarin red staining on day 28 (*E*). All error bars represent ±SD of three experimental replicates. (ns = not significant, ∗*p* ≤ 0.05, ∗∗*p* ≤ 0.01, ∗∗∗*p* ≤ 0.001, and ∗∗∗∗*p* ≤ 0.0001). RT-qPCR, real-time quantitative PCR.

Ezh2 inhibition promotes differentiation of MC3T3 preosteoblasts as evidenced by reduced H3K27me3 levels (Fig. S1*A*) and increased calcium deposition and alizarin red staining in cell cultures treated with EPZ6438 (Fig. S1*B*), consistent with previous observations (23). To understand the kinetics of Ezh2 inhibition, we performed a time course with 2.5 μM EPZ6438 in MC3T3 cells. H3K27me3 levels are reduced after 12 h of drug administration, and this effect persists for at least 72 h (Fig. 1*A*). To evaluate the impact of EPZ6438 on osteogenesis, MC3T3 cells were exposed to 2.5 μM EPZ6438 for the initial 3 days of a 28-day osteoblast differentiation protocol, with RNA and protein collected and alizarin staining performed at designated intervals (Fig. 1*B*). EPZ6438-mediated H3K27me3 reduction is associated with increases in H3K27ac and Runx2 levels, but no changes in H3K4me3, H3K9me3, Ezh2, or other PRC2 components are observed (Figs. 1*C* and S2). EPZ6438 induces mRNA expression of Runx2-p57 and several bone mineralization markers including Alpl, Bglap, Phex, Ibsp, and Phospho1 (Fig. 1*D*). Alizarin red staining revealed that EPZ6438 enhances calcium deposition in osteogenic medium, but not in growth medium lacking ascorbic acid and β-glycerol phosphate (Fig. 1*E*). Similar to immortalized MC3T3 cells, when compared to vehicle treatment, EPZ6438 (2.5 μM) reduced H3K27me3 levels and stimulated osteogenic gene expression in primary preosteoblasts derived from newborn mouse calvaria (Fig. S3). Taken together, EPZ6438-mediated reduction in H3K27me3 stimulates the levels of H3K27ac and Runx2, while accelerating osteogenic differentiation of MC3T3 cells and primary preosteoblasts.

### EPZ6438-mediated H3K27me3 reduction and activation of bivalent domains are associated with cAMP, WNT, and Hedgehog pathways

**Figure 2:**
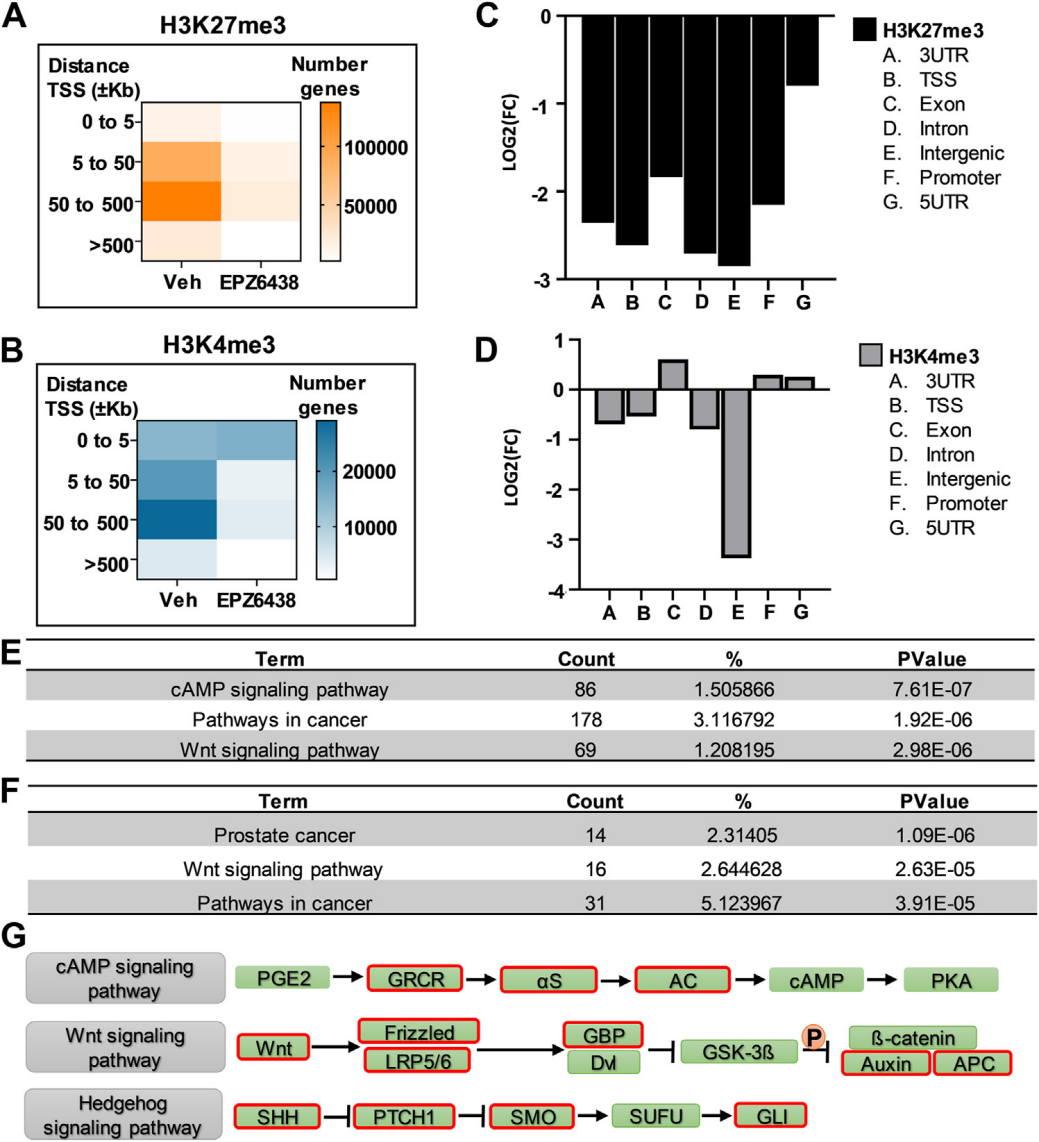
**Tazemetostat-mediated H3K27me3 reduction and activation of bivalent domains are associated with cAMP, WNT, and Hedgehog pathway.** Heatmap representation of number of genes with H3K27me3 (*A*) or H3K4me3 (*B*) enrichment in different distances away from TSS. Graphical representation of the fold change (EPZ6438 *versus* Veh) of H3K27me3 (*C*) and H3K4me3 (*D*) enrichment in relationship with genomic distribution. Gene ontology of genes with H3K27me3 reduction (*E*) and H3K4me3 (*F*) enrichment using DAVID. Schematic representation of cAMP, WNT, and Hedgehog associated with pathways in cancer (*G*). Red outline indicates genes with H3K27me3 reduction and H3K4me3 enrichment. TSS, transcriptional start site.

To explore the effects of EPZ6438 on bivalent domain–associated enhancers critical for bone formation, we assessed the genome-wide profiles for H3K4me3 and H3K27me3 modifications in differentiating MC3T3 cells in the presence or absence of EPZ6438 for 3 days. To understand the global changes in H3K4me3 and H3K27me3, we first evaluated the numbers of genes that exhibit changes in these two epigenetic marks. Robust reduction in H3K27me3 is observed in the EPZ6438-treated samples (Fig. 2*A*). However, a reduction in H3K4me3 is noted in EPZ6438-treated samples in regions located ±5 kb away from the transcriptional start site (TSS) (Fig. 2*B*). Interestingly, an increase in H3K4me3 is observed in genomic regions that are within 5 kb of the TSS (Fig. 2*B*). Analysis of the genomic distribution revealed a reduction in H3K27me3 levels in all genomic regions with EPZ6438 addition (Figs. 2*C* and S4*A*). Similarly, a reduction in H3K4me3 enrichment is observed in 3′UTR, TSS, intronic, and intergenic regions with EPZ6438 treatment (Figs. 2*D* and S4*B*). However, an increase in H3K4me3 levels is noted in exons, promoters, and 5′UTR regions after treatment with EPZ6438.

To define pathways that may be altered by EPZ6438 and be involved in osteogenic differentiation, the genes associated with alterations in H3K4me3 and H3K27me3 were assessed using gene ontology analysis with the DAVID 6.8 functional analysis tool (44). The genes that exhibit an EPZ6438-dependent reduction in H3K27me3 levels (n = 6288) are highly enriched (*i.e.*, top three categories) for cAMP signaling pathway, pathways in cancer, and the WNT signaling pathway (Fig. 2*E*). As bivalent genes are marked by H3K4me3 and H3K27me3, we next evaluated genes that exhibited a reduction in H3K27me3 but had no change or exhibited an increase in H3K4me3. This analysis revealed a set of genes (n = 616) that are associated with prostate cancer, the WNT signaling pathway, and pathways in cancer (Fig. 2*F*). A closer examination of the subset of genes associated with the “pathways in cancer” category that exhibit a reduction in H3K27me3 revealed that this group intersects with genes (outlined in red boxes) involved in cAMP, WNT, and Hedgehog (Hh) signaling (Fig. 2*G*). Thus, these results indicate that the reduction in H3K27me3 and concomitant retention or increase in H3K4me3 levels are associated with key genes involved in cAMP, WNT, and Hh signaling pathways.

Analysis of histone mark enrichment between EPZ6438 and vehicle was performed using DiffBind (45). The binding differences for H3K27me3 and H3K4me3 between vehicle and EPZ6438 are indicated by volcano plots (Fig. S5, *A* and *B*). Of note, the number of differentially regulated peaks is more robust for the H3K27me3 mark when compared to the H3K4me3 mark. Interestingly, while H3K27me3 is generally reduced with EPZ6438 (Fig. S5*C*), the levels of H3K4me3 are enriched in some genes and reduced at other loci in the presence of EPZ6438 (Fig. S5*D*). Because loss of H3K27me3 may activate pro-osteogenic and repress anti-osteogenic pathways, changes in H3K4me3 represent the indirect consequences of EZH2-related regulatory events.

**Figure 3:**
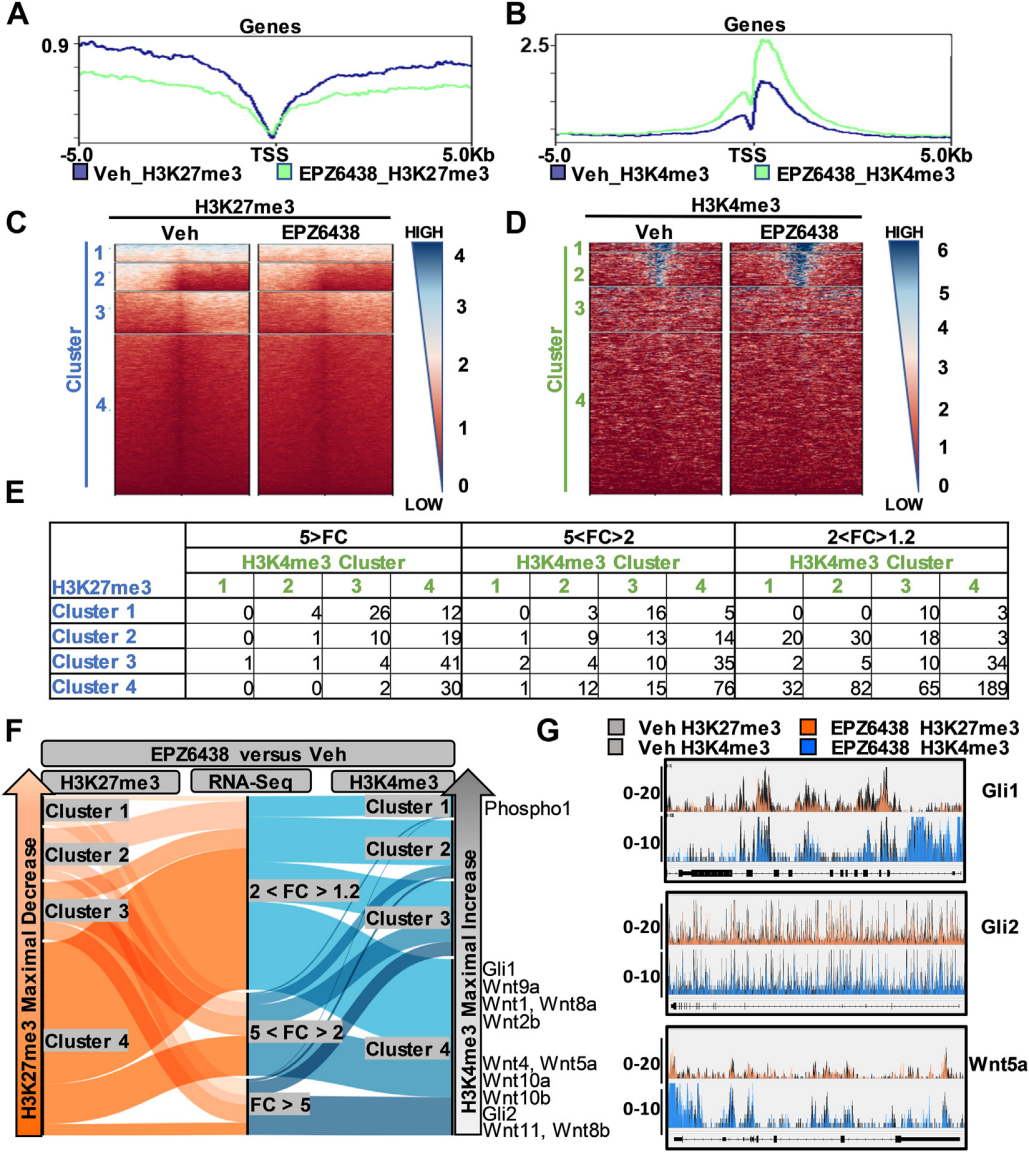
**WNT and hedgehog pathway genes associated with H3K27me3 reduction and H3K4me3 enrichment upon EPZ6438 treatment.** H3K27me3 and H3K4me3 distribution at promoters from MC3T3 cells treated with Veh or EPZ6438 for 3 days. ChIP-seq analysis was performed within a region spanning ±5 kb around TSS. TSS profile plot of the H3K27me3 (*A*) and H3K4me3 (*B*) show the average ChIP signal for Veh (*blue*) and EPZ6438 (*green*) treatment. Profile heatmap around TSS of RefSeq genes was represented with gradient *blue-to-red*, this color indicates *high-to-low counts* in the corresponding region. Consensus H3K27me3 and H3K4me3 bounds peaks are separated into four clusters by K-means clustering (*C*–*E*). The changes in H3K27me3 (*C*, *blue color*) and in K3K4me3 (*D*, *green color*) are illustrated by heatmaps. Overlay between gene expression changes by RNA-seq analysis and alterations in H3K27me3 and H3K4me3 levels upon Ezh2 inhibition by EPZ6438 (*E*). Upregulated genes (RNA-seq) were subdivided into three groups based on fold change (FC) relative to vehicle (5 > FC, 5 < FC > 2, 2 < FC > 1.2). The changes in gene induction are correlated with alterations in H3K27me3 (*blue color*) and H3K4me3 (*green color*). The correlation between gene expression (RNA-seq) and H3K27me3 and H3K4me3 levels due to EPZ6438 treatment is further highlighted by an *Alluvial plot* (*F*). Genes associated with WNT and hedgehog pathways highlighted on the *right*. Genome browser view of H3K27me3 and H3K4me3 was performed for Gli1, Gli2, and WNT5a (*G*). ChIP, chromatin immunoprecipitation; TSS, transcriptional start site.

To define bivalent genes and pathways relevant to osteogenesis that are differentially regulated upon Ezh2 inhibition in MC3T3 cells, we overlayed the osteoblast epigenome (ChIP-seq) with the osteoblast transcriptome (RNA-seq). Bivalent domains are typically located at gene promoters. Histogram data analysis revealed H3K27me3 reduction and H3K4me3 enrichment in genomic regions within 5 kb of the TSS in cells treated with EPZ6438 (Fig. 3, *A* and *B*). The Model-based Analysis of ChIP-Seq (MACS) algorithm was used to identify genes that are significantly enriched for each histone mark within regions centered at the canonical TSS (±5 kb). Using deepTool (http://deeptools.ie-freiburg.mpg.de) (46), K-means clustering revealed unique epigenetic patterns for H3K27me3 (Fig. 3*C*) and H3K4me3 (Fig. 3*D*) between vehicle- and EPZ6438-treated cells. To correlate these epigenetic changes to gene expression, we divided EPZ6438-induced genes based on expression intensity (FC < 5, 5 < FC > 2, 2 < FC > 1.2) and H3K27me3 and H3K4me3 clustering (Fig. 3*E* and Tables S1–S3). These data were also correlated with K-means clustering by an alluvial diagram (Fig. 3*F*). As anticipated, this analysis determined that many genes that exhibit a reduction in H3K27me3 following EPZ6438 treatment show robust gene expression changes (FC > 2, Fig. 3*F*). Notably, multiple genes that are involved in WNT (*e.g.*, WNT4 and WNT5a) and Hh (*e.g.*, Gli1 and Gli2) signaling pathways (also see Fig. 2) are present in this group (Fig. 3*G*). Some of these genes show a gain in H3K4me3 levels in their promoters consistent with enhanced expression. Together, integrative analysis of RNA-seq and chromatin immunoprecipitation (ChIP)-seq datasets indicate that the pro-osteogenic effects induced by EPZ6438 may involve WNT and Hh signaling pathways.

### Inhibition of the WNT pathway modulates the pro-osteogenic effects of EPZ6438

**Figure 4:**
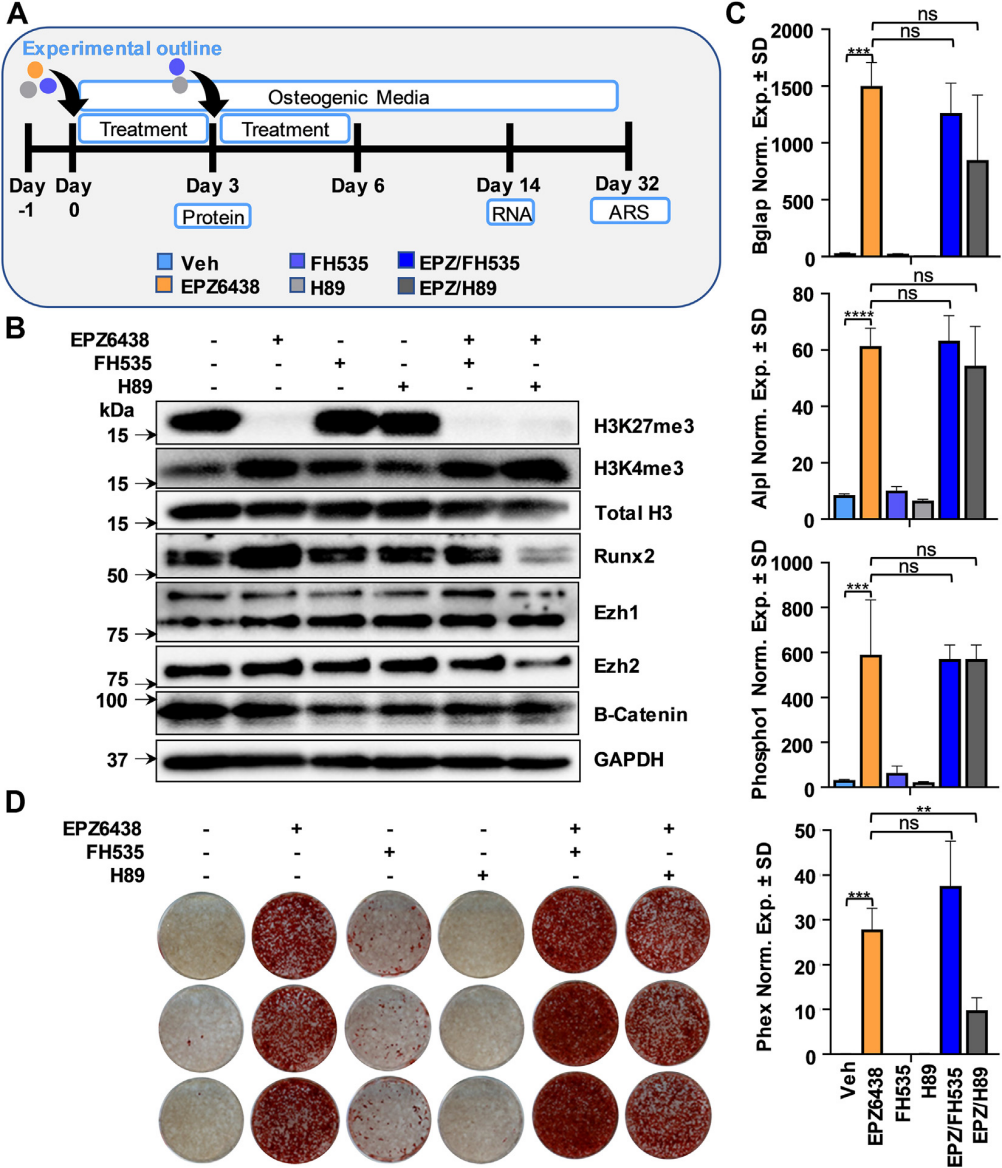
**Pro-osteogenic effects induced by Ezh2 inhibition are enhanced by disruption of WNT pathway.** Diagram of the experimental protocol for treatment of MC3T3 with vehicle (Veh) or 2.5 μM EPZ6438 with or without 5 μM FH535 or H89 shown in panels *B*–*D* (*A*). Western blotting analysis on day 3 (*B*). RT-qPCR analysis for bone-related markers relative to Gapdh (set at 100) on day 14 (*C*). Alizarin red staining on day 32 (*D*). All error bars represent ± SD of three experimental replicates (ns = not significant, ∗*p* ≤ 0.05, ∗∗*p* ≤ 0.01, ∗∗∗*p* ≤ 0.001, and ∗∗∗∗*p* ≤ 0.0001). RT-qPCR, real-time quantitative PCR.

Reduction in H3K27me3 and a concomitant increase in H3K4me3 are observed in promoters of genes involved in cAMP, WNT, and cancer pathways upon Ezh2 inhibition (see Fig. 2). These pathways are of interest because WNT signaling regulates bone formation and cooperates with parathyroid hormone and bone morphogenetic proteins (BMPs) to maintain bone density (47) and cAMP/protein kinase A (PKA) is a vital intracellular mediator of G protein coupled receptors like PTH1 receptor, which regulates osteogenic differentiation and mineralization (48). Using the WNT and cAMP signaling inhibitors FH535 and H89, we tested the involvement of these pathways on the pro-osteogenic effects induced by EPZ6438 (Fig. 4*A*). EPZ6438-mediated H3K27me3 reduction is associated with an increase in Runx2 expression (Fig. 4*B*), corroborating earlier results (see Fig. 1). As anticipated (49, 50, 51), β-catenin protein levels are reduced with FH535 and H89 (Figs. 4*B* and S6). H3K4me3, Runx2, Ezh1, and Ezh2 protein levels (Fig. 4*B*) and mRNA expression of typical osteogenic genes (Fig. 4*C*) are not affected by FH535. Nevertheless, this WNT inhibitor increased mineral deposition in combination with EPZ6438 (Figs. 4*D* and S7). H89 reduced Runx2 protein (Fig. 4*B*) and Phex mRNA expression in the presence of EPZ6438 (Fig. 4*C*), but this cAMP inhibitor did not affect matrix mineralization alone or when combined with EPZ6438 (Figs. 4*D* and S7). When compared to single drug application, the combination of FH535 and H89 did not further alter the osteogenic phenotype associated with EPZ6438 as evaluated by alizarin red staining (Fig. S8). Collectively, these results indicate that the WNT pathway that is activated by Ezh2 inhibition also modulates the response to Ezh2. The latter suggests that the WNT pathway is linked to Ezh2-mediated events *via* molecular feedback or feedforward mechanisms.

### Silencing of Gli1 increases mineralization by MC3T3 preosteoblasts

**Figure 5:**
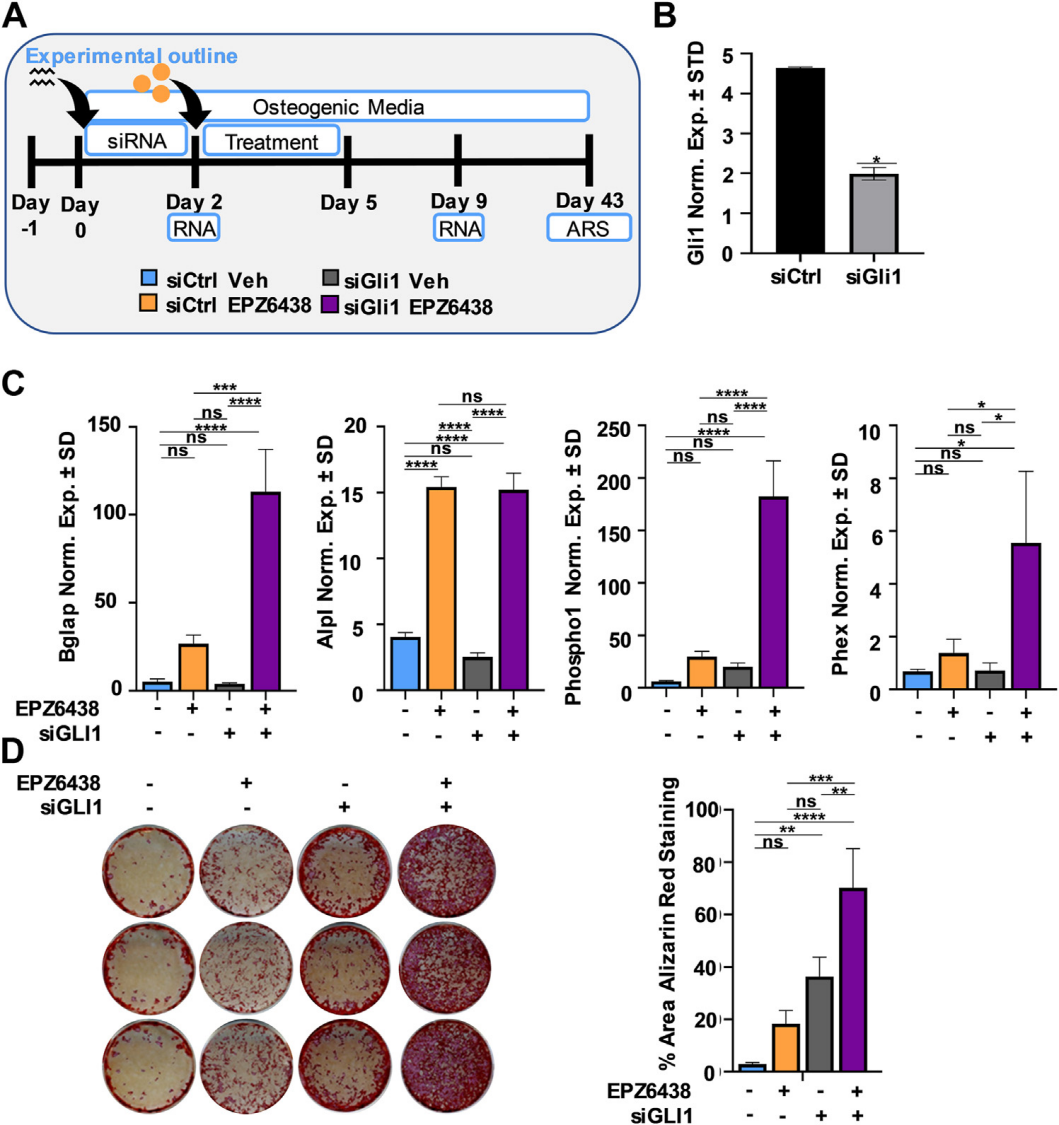
**Silencing of Gli1 increasesmineralization inMC3T3preosteoblast in the absence or presence of EPZ6438.** Diagram of the experimental protocol for treatment of MC3T3 with siCtrl or siGli1 and with vehicle (Veh) or 2.5 μM EPZ6438 shown in panels *B*–*D* (*A*). Gli1 mRNA levels as evaluated by RT-qPCR on day 2 (*B*). RT-qPCR analysis for bone-related markers relative to Gapdh (set at 100) on day 9 (*C*). Alizarin red staining on day 43 (*D*). All error bars represent ±SD of three experimental replicates. (ns = not significant, ∗*p* ≤ 0.05, ∗∗*p* ≤ 0.01, ∗∗∗*p* ≤ 0.001, and ∗∗∗∗*p* ≤ 0.0001). RT-qPCR, real-time quantitative PCR.

Like WNT and cAMP signaling, H3K27me3 reduction and H3K4me3 enrichment were also observed with genes associated with the Hh signaling pathway, including Gli1 and Gli2 (see Fig. 2). Hh ligands bind to protein patched homolog 1 (PTC), a conserved receptor that activates the GLI family of transcription factors, which are involved in development, disease, and skeletal repair (52). To evaluate the contribution of this pathway on enhanced osteogenic differentiation by EPZ6438, MC3T3 preosteoblasts were transfected with Gli1 siRNA, followed by EPZ6438 treatment 2 days later (Fig. 5*A*). The reduction in Gli1 mRNA expression just prior to EPZ6438 addition was confirmed by real-time quantitative PCR (RT-qPCR) (Fig. 5*B*). Suppression of Gli1 and concomitant treatment with EPZ6438 creates synergistic effects as demonstrated by a strong transcriptional activation of osteogenic genes like Bglap, Phospho1, and Phex (Fig. 5*C*) as well as increased matrix mineralization (Fig. 5*D*). These results indicate that loss of Gli1 increases the pro-osteogenic effects of the Ezh2 inhibitor, EPZ6438.

**Figure 6:**
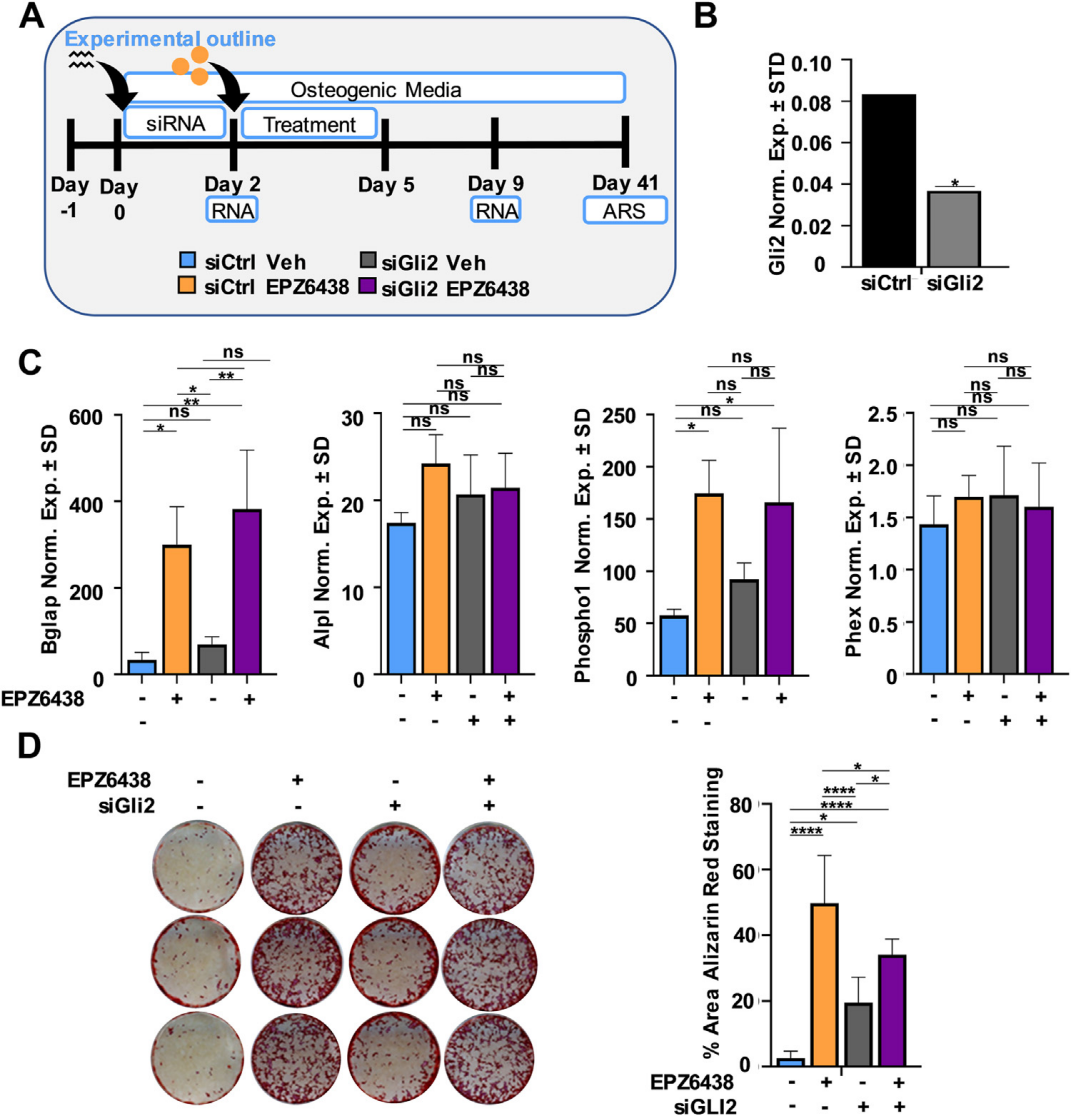
**Silencing of Gli2 inMC3T3preosteoblast does not alter EPZ6438-mediated osteogenic differentiation.** Diagram of the experimental protocol for treatment of MC3T3 with siCtrl or siGli2 and with vehicle (Veh) or 2.5 μM EPZ6438 shown in panels *B*–*D* (*A*). Gli1 mRNA levels as evaluated by RT-qPCR on day 2 (*B*). RT-qPCR analysis for bone-related markers relative to Gapdh (set at 100) on day 9 (*C*). Alizarin red staining on day 41 (*D*). All error bars represent ±SD of three experimental replicates. (ns = not significant, ∗*p* ≤ 0.05, ∗∗*p* ≤ 0.01, ∗∗∗*p* ≤ 0.001, and ∗∗∗∗*p* ≤ 0.0001). RT-qPCR, real-time quantitative PCR.

Similar to Gli1, we also silenced Gli2 just prior to the addition of EPZ6438 (Fig. 6, *A* and *B*). When compared to EPZ6438 treatment alone, combination of EPZ6438 and Gli2 depletion does not further enhance the expression of osteogenic markers (Fig. 6*C*) or mineral deposition (Fig. 6*D*). However, Gli2 depletion alone enhances mineral deposition in MC3T3 cells. These results indicated that Gli2 is not involved in EPZ6438-mediated enhancement of osteogenic differentiation.

**Figure 7:**
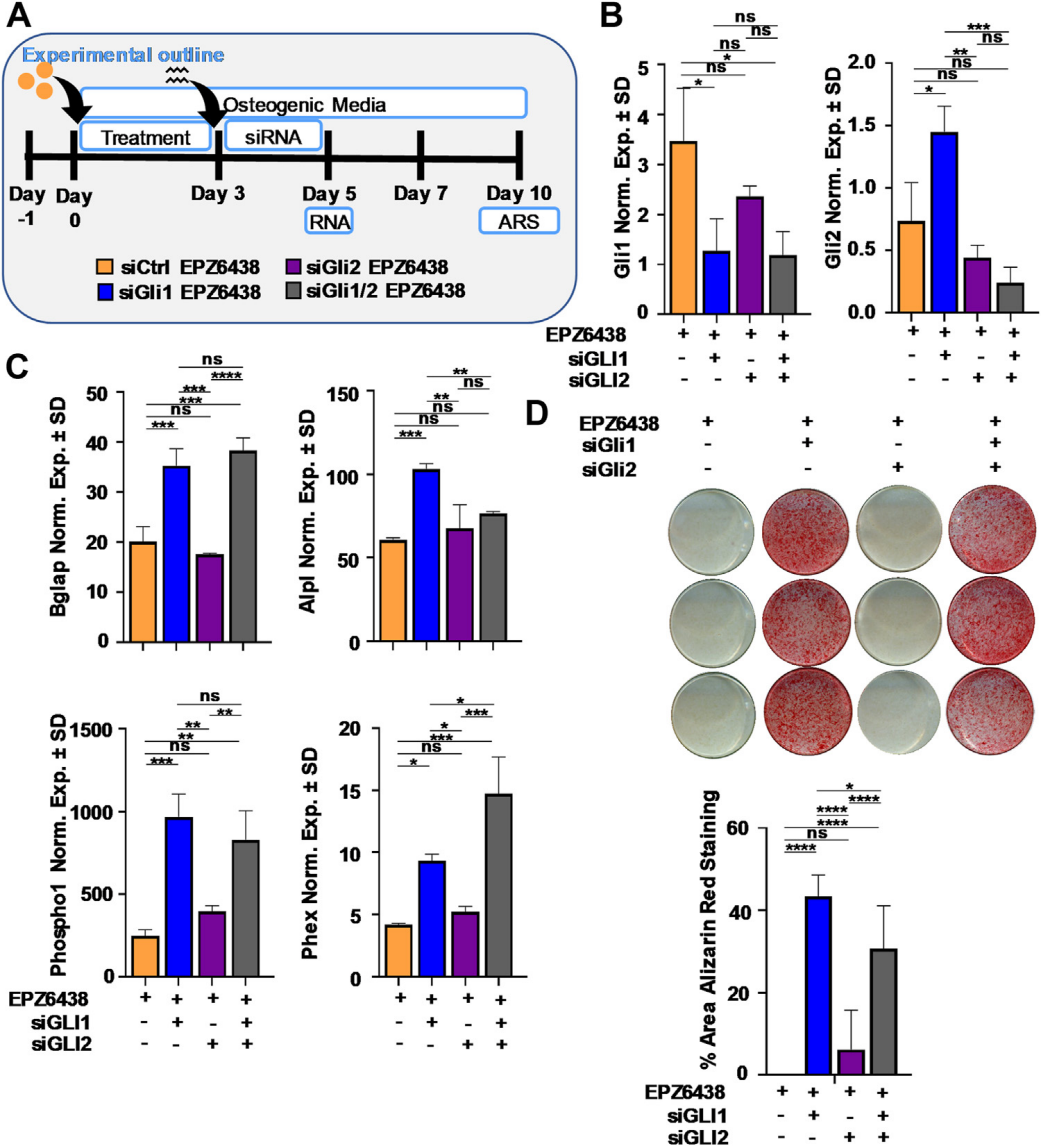
**Silencing of Gli1 and Gli2 increases the mineralization inMC3T3preosteoblast pretreated with EPZ6438.** Diagram of the experimental protocol for treatment of MC3T3 with siCtrl, siGli1, or siGli2 and with vehicle (Veh) or 2.5 μM EPZ6438 shown in panels *B*–*D* (*A*). Gli1 and Gli2 mRNA levels as evaluated by RT-qPCR on day 5 (*B*). RT-qPCR analysis for bone-related markers relative to Gapdh (set at 100) on day 7 (*C*). Alizarin red staining on day 10 (*D*). All error bars represent ±SD of three experimental replicates (ns = not significant, ∗*p* ≤ 0.05, ∗∗*p* ≤ 0.01, ∗∗∗*p* ≤ 0.001, and ∗∗∗∗*p* ≤ 0.0001). RT-qPCR, real-time quantitative PCR.

To assess the impact of Gli family members loss in cells pretreated with EPZ6438, MC3T3 preosteoblasts were treated for 3 days with EPZ6438, followed by Gli1 and Gli2 depletion (Fig. 7*A*). qRT-PCR analysis validated Gli1 and Gli2 depletion and revealed that silencing of Gli1 increases Gli2 expression (Fig. 7*B*). As observed earlier, Gli1 depletion (alone or in combination with loss of Gli2) enhances osteogenic gene expression (Fig. 7*C*) and mineral deposition (Fig. 7*D*) of MC3T3 cells pretreated with EPZ6438. Interestingly, pretreatment with EPZ6438 followed by Gli1 depletion (Fig. 7) results in robust matrix deposition on day 10, while Gli1 depletion followed by EPZ6438 treatment (Fig. 5) yields a robust matrix on day 26 (Fig. S9). Together, our results demonstrate that Gli1 is a suppressor of EPZ6438-mediated mineralization and that its activation may be a component of a negative feedback mechanism that counters the effects of unscheduled Ezh2 inactivation.

## Discussion

Bivalent chromatin domains have primarily been described in embryonic stem cells, where alterations in these regions control the expression of lineage-driving genes as cells differentiate toward a particular phenotype (7, 53). Epigenetic regulators governing bivalent promoters include histone methyltransferases such as Ezh2 (7). Studies in normal cells suggest that tissue-specific transcription factors recruit PRC2 to add H3K27me3 at gene loci to prevent aberrant gene expression (54, 55). Our present study shows that the Ezh2 inhibitor EPZ6438 enhances osteogenic differentiation through the global loss of H3K27me3 in the presence of osteogenic cues (ascorbic acid and β-glycerol phosphate). This concept is supported by previous work which revealed that progenitor cells respond rapidly to changing levels of morphogens and other intracellular signals that control the spatial and temporal patterning during development. In this regard, stem cells are maintained in a quiescent state until the microenvironment changes and sends signals to promote either selfrenewal or differentiation (56). Our present studies are also supported by studies from our group and others on the role of Ezh2 and its inhibitors on osteogenic differentiation and MSC renewal (18, 22, 23, 24, 25, 26, 27, 33, 34, 37, 41, 57).

Enhancer activation is initiated by transcription factors and is often followed by H3K4me1 deposition and H3K27 acetylation (58). Using EPZ6438, we show that, as anticipated, Ezh2 inhibition causes global loss of H3K27me3 and a dramatic increase in H3K27ac levels (23, 59). These results are also consistent with previous reports that showed that loss of PRC2 activity resulted in H3K27ac marks preferentially accumulating at promoters and distal regulatory sites (60). The global change in histone modifications in cells lacking PRC2 components such as Suz12 (61) or Eed (62) reveal that inactivation of PRC2 results in a methylation to acetylation shift. This shift correlates with transcriptional activation through the recruitment of RNA-Pol2, which is facilitated by bromodomain proteins that recognize H3K27ac (*e.g.*, BRD2, BRD4) and stimulate osteoblast differentiation (59, 63, 64).

Studies in undifferentiated cells postulated that bivalently marked lineage-specific genes are kept transcriptionally inactive by H3K27me3 (53, 65). Loss of PRC2 components results in a loss of H3K27me3 and partial activation of genes that are bivalently repressed to support pluripotency (61, 66, 67, 68). In addition, lineage-specific genes are kept transcriptionally poised by H3K4me3 to facilitate potential activation (53, 65). By combining chromatin immunoprecipitation and RNA-seq, we analyzed H3K4me3 and H3K27me3 modification patterns in MC3T3 preosteoblasts. Genomic distribution between these histone marks revealed a H3K27me3 reduction in all the genomic regions and increased levels of H3K4me3 around exon, promoter, and 5′UTR regions upon treatment with EPZ6438. This is consistent with studies showing that H3K27me3 extends over a broader range associated with gene repression, while H3K4me3 accumulates around TSS and positively correlates with increased gene expression during differentiation (69, 70). Together, our studies and existing literature indicate that at gene promoters, occupancy of polycomb complexes and their mediated covalent histone modifications are critical for maintaining an undifferentiated state by preventing stochastic transcription of lineage differentiation genes (60, 71, 72).

Osteoblasts, which originate from MSC precursors, are the cells which synthesize and mineralize a collagenous matrix to form mature bone (42). Different signaling pathways have been described to control and support this process, including bone morphometric proteins, WNT-induced signaling, insulin-like growth factor, parathyroid hormone–related peptide, and fibroblast growth factors (73). In this study, we identified signaling pathways that are involved in the enhancement of osteogenesis through Ezh2 inhibition. Genes that exhibit H3K27me3 reduction with EPZ6438 were highly enriched for the cAMP pathway, while genes that exhibited both reduced H3K27me3 and increased H3K4me3 were associated with pathways in cancer, and the WNT pathway. Integrative analysis of ChIP-seq and RNA-seq data showed that these pathways are associated with bivalent epigenomic regulatory regions. We assessed the contribution of these pathways on the enhanced osteogenic differentiation by EPZ6438 with inhibitors that target the WNT/β-catenin (FH535) and cAMP/PKA pathway (H89). Both inhibitors have been reported to reduce protein expression of β-catenin (49, 50, 51, 74), which is a part of the canonical WNT signaling pathway. Our study showed that inhibition of the WNT pathway with FH535 enhances the EPZ6438-mediated mineralization of MC3T3 osteoblasts. WNT ligands can activate four different signaling pathways: the canonical WNT/β-catenin pathway, the noncanonical planar cell polarity (PCP) pathway, the WNT-Ca2+, and the PKA pathways (75). Thus, our results establish that activation of noncanonical members of the WNT family (*i.e.*, WNT4, WNT5a, WNT7, and WNT11; see Fig. 3) supports EPZ6438-mediated osteogenic differentiation (76). The latter finding is consistent with the importance of the PCP pathway in supporting osteoblast orientation along lines of strain (77). The interaction between WNT signaling and Ezh2 was also highlighted in our previous studies showing that β-catenin activated Ezh2 activity in MSCs to maintain stemness and selfrenewal (57). Initial studies on the combined inhibition of the WNT (FH535) and cAMP pathways (H89) in the presence of EPZ6438 revealed that the combination of FH535 and H89 does not reduce the pro-osteogenic effects of EPZ6438. Therefore, a deeper mechanistic understanding of how WNT and cAMP signaling are activated by Ezh2 inhibition will require analysis of specific genes related to WNT and cAMP function rather than small molecule inhibition.

It has been challenging to reconcile the *in vivo* and *in vitro* effects of Ezh2 inhibition, which clearly are bone stimulatory (18, 22, 23, 24, 25, 26, 27, 28, 29, 30, 31, 32, 33, 34, 35, 36, 37, 38, 39, 40, 41), with conditional Ezh2 gene KO studies (22, 24, 27, 39). Conditional loss of Ezh2 in mesenchymal cells accelerates suture fusion by promoting mineralization of calvarial osteoblasts (27), but ablation of Ezh2 in osteoblastic cells results in a low trabecular bone phenotype in young mice (27). Complicating factors here are the timescales of bone formation and homeostasis and cell autonomous molecular mechanisms, as well as effects on progenitor populations, compensation by Ezh1 and permanent loss of a protein *versus* its transient enzymatic inhibition. Persistent loss of Ezh2 may interfere with cell cycle progression, and thus reduce the number of osteoblasts (25, 27), while acute and short-term inactivation of Ezh2 *via* small molecules (*e.g.*, GSK126 and EPZ6438) appears to activate the osteogenic process within progenitors, thus driving osteogenic commitment and stimulating bone formation *in vivo* (22, 23, 25). Hence, *in vivo* strategies that can harness the desired effects (*i.e.*, enhanced osteogenesis and bone formation) and reduce the potential side effects (*i.e.*, cell cycle arrest) need to be developed to harness the clinical potential of Ezh2 inhibitors such as EPZ6438.

The Hh signaling pathway was also associated with H3K27me3 reduction and H3K4me3 enrichment in our present studies. Several studies have revealed that Hh signaling is a key pathway for the development and maintenance of skeletal tissue (78, 79, 80, 81). The locus for Gli1, which is a transcription factor associated with Hh signaling, contains bivalent chromatin domains and Hh activation has been associated with a local switch from suppressive to activating epigenetic cofactors (79). Our results show that silencing of Gli1 increases osteogenic differentiation in preosteoblast in the presence of EPZ6438, suggesting that Gli1 acts as a repressor of osteogenic differentiation. Because Gli1 is activated by Ezh2 inhibition, it appears that cells may upregulate Gli1 in response to reduced Ezh2 activity to prevent precocious differentiation. Coinhibition of both Ezh2 and the Hh–Gli pathway may override this putative negative feedback mechanism and further drive osteogenic differentiation.

EPZ6438 is a pharmacological Ezh2 inhibitor with *in vitro* properties similar to a previously reported compound (GSK126) characterized in our group (22), but with superior potency and oral bioavailability (82). In this report, we have demonstrated that treatment with EPZ6438 leads to global alterations in the histone methylome, including changes in bivalent domains of key osteogenic regulators. Beyond establishing additional mechanistic insight into the role of Ezh2 on osteoblast differentiation, our present studies further confirm the pro-osteogenic properties of Ezh2 inhibitors (18, 22, 23, 24, 25, 26, 27, 28, 29, 30, 31, 32, 33, 34, 35, 36, 37, 38, 39, 40, 41). In general, short-term inhibition or loss of Ezh2 is associated with enhanced osteogenic differentiation and bone formation. However, it is important to point out that persistent Ezh2 inactivation in progenitors also stimulates expression of Cdkn2a and impairs cell cycle progression (24, 27). Thus, optimal strategies that can harness the desired effects (*i.e.*, enhanced osteogenesis and bone formation) and reduce the potential side effects (*i.e.*, cell cycle arrest) should be considered for potential clinical use of Ezh2 inhibitors. Such strategies may involve epigenetic (re)programing of progenitor cells with Ezh2 inhibitors such as EPZ6438 *ex vivo* before implantation or *via* local applications *in vivo* to achieve the desired bone anabolic effects and reduce the negative effects associated with cell cycle arrest. Indeed, we have previously shown that Ezh2 inhibition facilitates BMP2-mediated osteogenic differentiation of progenitor cells and maturation of committed osteoblasts *in vitro* (25). Follow-up studies showed that local BMP2 and Ezh2 inhibitor (GSK126) administration in conjunction with short-term systemic Ezh2 inhibition results in more consistent bone healing when compared to local BMP2 alone in a mouse calvarial defect model (25). Similarly, Ezh2 inhibition and BMP2 costimulate osteogenic differentiation of MSCs grown on 3D scaffolds *in vitro* (39). Together, these previous studies suggest that local and short-term application of Ezh2 inhibitors alone or in combination with potent bone anabolic agents (*e.g.*, BMP2) may provide a strategy to stimulate local bone formation and such strategies could provide a viable option for local bone applications (*e.g.*, fractures, nonunions, spinal fusion).

We show that EPZ6438-mediated osteogenic differentiation modulates components of the cAMP, WNT, and Hh–Gli1 pathways. WNT and Hh–Gli1 are normally suppressed by Ezh2, and the induction of these pathways upon Ezh2 inactivation may prevent accelerated osteogenesis as part of a negative feedback mechanism. The observation may provide the basis for the development of new therapeutic drug combination strategies for the treatment of low-bone density. Our integrated transcriptomic, epigenomic, and functional analyses collectively indicate that noncanonical WNT antagonists and Hh-Gli1 antagonists could be considered in synergistic therapies in combination with Ezh2 inhibitors.

## Experimental procedures

### Isolation of primary mouse preosteoblasts derived from newborn calvarial

Calvarial pre-osteoblasts were isolated from 1 day old C57/BL6 pups as previously described (27). Briefly, the calvarial tissues were removed and washed in 1× PBS buffer solution three times to deplete blood cells. All these operations were under sterile condition. TrypLE Express Enzyme (Gibco) was added to the bone chips for 25 min at 37 °C to remove fibrous tissue. Thereafter, the tissues were cut into 1 to 2 mm^3^ pieces. Bone chips were digested with 0.1% w/v Collagenase I (Sigma) for 1 h in the shaking incubator at 37 °C with shaking speed of 200 rpm. The resulting cells were collected by centrifugation for 5 min at 1000 rpm. The cell pellet was resuspended in 10 ml of α-Minimal Essential Medium (α-MEM) (Gibco) supplemented with 10% fetal bovine serum (FBS; R&D Systems) and 100 units/ml penicillin (Gibco).

### Cell maintenance media and osteogenic differentiation

MC3T3-E1 subclone 4 cells (referred to as MC3T3 cells) and primary mouse preosteoblasts were maintained and expanded in ascorbic acid–free α-MEM supplemented with 10% FBS (R&D Systems) and 100 units/ml penicillin (Gibco). Cells were plated at a density of 10,000 cells/cm^2^ on standard tissue culture plates (Corning) in maintenance medium. The following day, maintenance medium was replaced with osteogenic medium which consisted of α-MEM supplemented with 10% FBS, 100 units/ml penicillin, 50 μg/ml ascorbic acid (Sigma), and 10 mM beta-glycerol phosphate (Sigma). Treatments were performed by adding vehicle (dimethyl sulfoxide [DMSO]) or EPZ6438 at intervals described below. Osteogenic medium was replaced every 3 days.

### Alizarin red staining

At the indicated time points, medium was removed, and cells were washed twice with PBS. After washing, cells were fixed in 10% neutral-buffered formalin for 1 h. Neutral-buffered formalin was then removed, cells were washed with PBS, and stained with 2% alizarin red pH 4.2 (Thermo Fischer Scientific) for 10 min. The stain was removed, and cells were washed five times with water. Images of the wells were taken, and staining was quantified using ImageJ program (https://imagej.nih.gov/ij/index.html).

### RNA-seq and ChIP-seq sample preparation

MC3T3 passage 9 cells were collected for next-generation sequencing studies. Cells were seeded in maintenance medium at a density of 10,000 cells/cm^2^. The next day, cells were treated with vehicle (DMSO) or 2.5 μM EPZ6438 (MedChem, HY-13803) in osteogenic medium. Three days later, the cells were harvested by trypsin and analyzed by ChIP assays using H3K27me3 (Cell Signaling, #9733 Lot 8) and H3K4me3 (Abcam, ab8580 Lot GR3264490-1) antibodies. DNA fragments were isolated using QIAquick PCR Purification Kit (Qiagen) (Fig. S10*A*). The quality and reproducibility of these studies are reflected by similarities in cell numbers and extent of DNA shearing between replicates and treatment groups (Fig. S10, *B*–*E*). For quality control following the ChIP assay, we evaluated H3, H3K4me3, and H3K27me3 enrichment in vehicle- and EPZ6438-treated samples. ChIP-qPCR revealed robust changes in H3K27me3 levels at the Neuro1 promoter and an unrelated intergenic region that are enriched for this epigenetic mark (83) in the presence of EPZ6438 (Fig. S4*F*). As anticipated (83), the promoter region of Actb did not exhibit enrichment for H3K27me3 in either the vehicle- or EPZ6438-treated samples. No appreciable differences were observed in H3 and H3K4me3 enrichment when comparing vehicle- and EPZ6438-treated samples (Fig. S10*F*).

Total RNA was isolated on day 3 of osteogenic differentiation using the Direct-zol RNA kit (Zymo Research) and TRIzol reagent (Invitrogen). Representative electropherograms used for DNA fragmentation quality analysis of ChIP-seq samples and RNA quality analysis of total RNA for each RNA-seq sample on Fragment Analyzer (Figs. S11 and S12).

### Chip-seq analysis

After alignment, PCR duplicates were removed with Picard software (http://broadinstitute.github.io/picard/). ChIP-seq peaks were called using the broad-peak function in MACS2 software (https://pypi.org/project/MACS2/) (84). For basic annotation of identified peaks, the HOMER function annotatePeaks.pl was used (85). The differential peak calling was done using DiffBind tool from MACS2 (45). Heatmaps and the plot profiles show the frequency of normalized number of reads per kilobase and per million mapped reads across genomic intervals with sizes of 5 kb or 200 kb to the TSS. Heatmaps were generated from merged biological replicate pairs for DMSO or EPZ6438 for respective histone modifications. These results were obtained using deepTOOLS software (46). A schematic representation of ChIP-seq protocol (Fig. S13*A*) and the number of reads for each sample are provided (Fig. S13*B*).

### Total RNA isolation

Cells were lysed, and RNA was extracted using TRI-Reagent (Zymo Research). RNA was isolated using DirectZol RNA MiniPrep (Zymo Research). Isolated RNA was quantified, and quality was tested using NanoDrop 2000 spectrophotometer (Thermo Fischer Scientific).

### Quantitative real time reverse transcription PCR

RNA was reverse transcribed into complementary DNA using a reverse transcription kit and protocol (Promega). Gene expression was quantified using RT-qPCR; each reaction was performed with 3.3 ng of complementary DNA, 0.08 μM of primer pairs, and 1X QuantiTect SYBR Green PCR Kit (Qiagen) using the CFX384 Real-Time System (Bio-Rad). Transcript levels were analyzed by the 2^−ΔΔCt^ method and normalized to the housekeeping gene GAPDH (set at 100). The primers used are described in Table S4.

### RNA-seq analysis

Transcript abundance was determined using the HTseq software (https://pypi.org/project/HTSeq/) (86) to calculate the raw read numbers for each gene. Differential gene expression was evaluated using DESeq2 (https://www.bioconductor.org/packages/DESeq2/) (87) within SARTools R package (88). Gene ontology analyses were carried out using the DAVID 6.8 (https://david.ncifcrf.gov/) (44). A schematic representation of RNA-seq protocol steps is provided in a figure format (Fig. S14). PCA and cluster dendrogram analysis show that the samples segregate into two groups depending on their treatment (Fig. S15, *A* and *B*). Volcano plot representation of differential gene expression shows that most genes (∼92%) remained unaltered with EPZ6438 treatment (Fig. S15*C*), while ∼4.5% of all genes are induced and ∼3.5% of genes are suppressed following EPZ6438 treatment (Fig. S15*D*).

### siRNA-mediated knockdown

MC3T3 cells were seeded in maintenance medium at 10,000 cells/cm^2^. One day later, siRNA transfections with nontargeting control (siCtrl) (Dharmacon ON-TARGETplus SMARTpools D-001810-10) or mouse Gli1, Gli2 (Dharmacon ON-TARGETplus SMARTpools L-047917-02, L043977-01) were performed using Lipofectamine RNAiMAX (Thermo Fischer Scientific) following the manufacturer’s instructions. Osteogenic medium was added 6 h after transfection. Medium was changed 2 days after transfection and replaced with fresh osteogenic medium containing vehicle (DMSO) or EPZ6438 (2.5 μM). Three days later, culture media for each of the treatment groups were removed and fresh osteogenic media were added. Subsequently, media were changed every 3 days.

### Western blotting

At the indicated time points, cells were washed with PBS and lysed with radioimmunoprecipitation buffer (150 mM NaCl, 50 mM Tris (pH 7.4), 1% sodium deoxycholate, 0.1% SDS, 1% NP-40) supplemented with protease inhibitor mixture (Sigma-Aldrich) and PMSF (Sigma-Aldrich). Lysates were cleared by centrifugation and stored at −80 °C. Protein concentrations were quantified using the detergent compatible protein assay kit (Bio-Rad) following the manufacturer’s protocol. Western blotting was performed as previously described (24). The antibodies used are described in Table S5.

### Statistical analysis

Experiments were performed using biological triplicates from cultures of MC3T3 cells. The results of the experiments are presented as mean ± SD. Statistical analysis was performed using one-way ANOVA or two-way ANOVA and Tukey’s or Dunnett’s test for multiple comparisons. Statistical analysis was performed using GraphPad Prism Software version 8.4.2 (https://www.graphpad.com/). Significance is noted in the figures when applicable (∗*p* < 0.05, ∗∗*p* < 0.01, ∗∗∗*p* < 0.001, and ∗∗∗∗*p* < 0.0001).

## Data availability

The data that support the findings of this study are available in Gene Expression Omnibus database at https://www.ncbi.nlm.nih.gov/geo/, Accessions #GSE196583.

## Supporting information

This article contains supporting information.

## Conflict of interest

The authors declare that they have no conflicts of interest with the contents of this article.
