# Supplementary material for: Inhibition of Ezh2 redistributes bivalent domains within transcriptional regulators associated with WNT and Hedgehog pathways in osteoblasts
Source: J Biol Chem. 2023 Aug 11;299(9):105155. doi: 10.1016/j.jbc.2023.105155 (PMC10506106; doi:10.1016/j.jbc.2023.105155)
Supplement: Supporting information [file mmc1.pdf]

Inhibition of the epigenetic regulator Ezh2 redistributes bivalent domains within transcriptional regulators associated with WNT and Hedgehog pathways in osteoblasts

Margarita E. Carrasco[1], Roman Thaler[1], Gino Nardocci[2], Amel Dudakovic[1,3#], Andre J van Wijnen[4#]

[1]Department of Orthopedic Surgery, Mayo Clinic, Rochester, MN, USA
[2]Universidad de los Andes, Santiago, Chile
[3]Department of Biochemistry and Molecular Biology, Mayo Clinic, Rochester, MN, USA
[4]Department of Biochemistry, University of Vermont, Burlington, VT, USA

Figure S1: Doses-response of Ezh2 inhibition by EPZ6438 in MC3T3 pre-osteoblasts.
Figure S2: Western Blot quantification of Ezh2 inhibition by EPZ6438 in MC3T3 cells.
Figure S3: Ezh2 inhibition by EPZ6438 enhances osteogenic differentiation of primary mouse pre-osteoblast cells.
Figure S4: Differential binding analysis of H3K27me3 and H3K4me3 ChIP-seq peak data.
Figure S5: Graphical representation of the fold change of H3K27me3 and H3K4me3 enrichment in relationship with the genomic distribution.
Figure S6: Western Blot quantification of Ezh2 inhibition and disruption of WNT and cAMP pathway.
Figure S7: Alizarin Red quantification of pro-osteogenic effects induced by Ezh2 inhibition are enhanced by disruption of WNT and cAMP pathway.
Figure S8: Osteogenic effects induced by Ezh2 inhibition are not changed by simultaneous disruption of WNT and cAMP pathway.
Figure S9: Silencing of Gli1 increases mineralization of MC3T3 pre-osteoblast.
Figure S10: H3K27me3 and H3K4me3 ChIP-seq.
Figure S11: Representative electropherograms used for DNA fragmentation quality analysis of ChIP-seq samples on Fragment Analyzer.
Figure S12: Representative electropherograms used for RNA quality analysis of total RNA for each RNA-seq sample on Fragment Analyzer.
Figure S13: Schematic representation of ChIP-seq protocol.
Figure S14: Schematic representation of RNA-seq protocol steps.
Figure S15: RNA-seq differential gene expression analysis using DESeq2.
Table S1: EPZ6438-induced genes listed in figure 3E that exhibited change in gene expression (FC > 5) by RNA-seq that correlated with a reduction in H3K27me3 and presence or increase in H3K4me3 by K-means clustering.
Table S2: EPZ6438-induced genes listed in figure 3E that exhibited change in gene expression (5 < FC > 2) by RNA-seq that correlated with a reduction in H3K27me3 and presence or increase in H3K4me3 by K-means clustering.
Table S3: EPZ6438-induced genes listed in figure 3E that exhibited change in gene expression (2 < FC > 1.2) by RNA-seq that correlated with a reduction in H3K27me3 and presence or increase in H3K4me3 by K-means clustering.
Table S4: List of primers used in this study.
Table S5: List of antibodies used in this study.

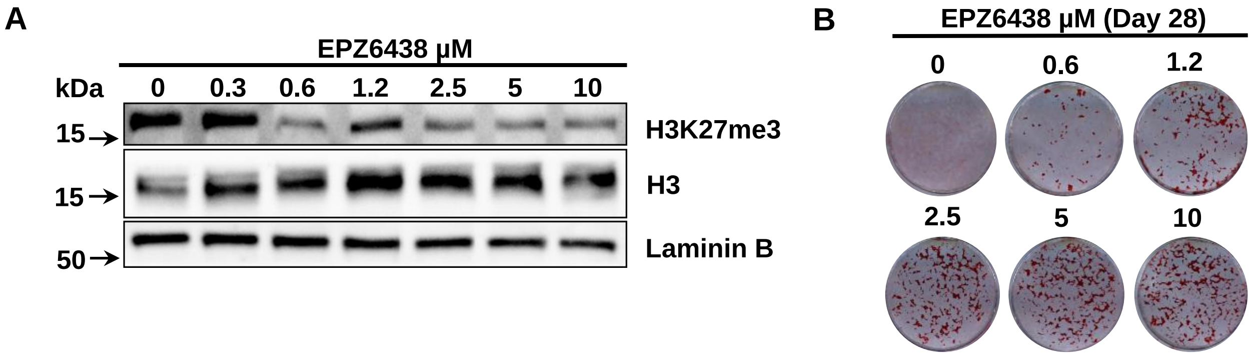

Figure S1. Dose-response of Ezh2 inhibition by EPZ6438 in MC3T3 pre-osteoblasts. Osteogenic differentiation was induced one day after plating. Vehicle and various EPZ6438 concentrations were added on the first day and removed on the third of differentiation. Western blot analysis on day three (A) and Alizarin red staining on day 28 (B).

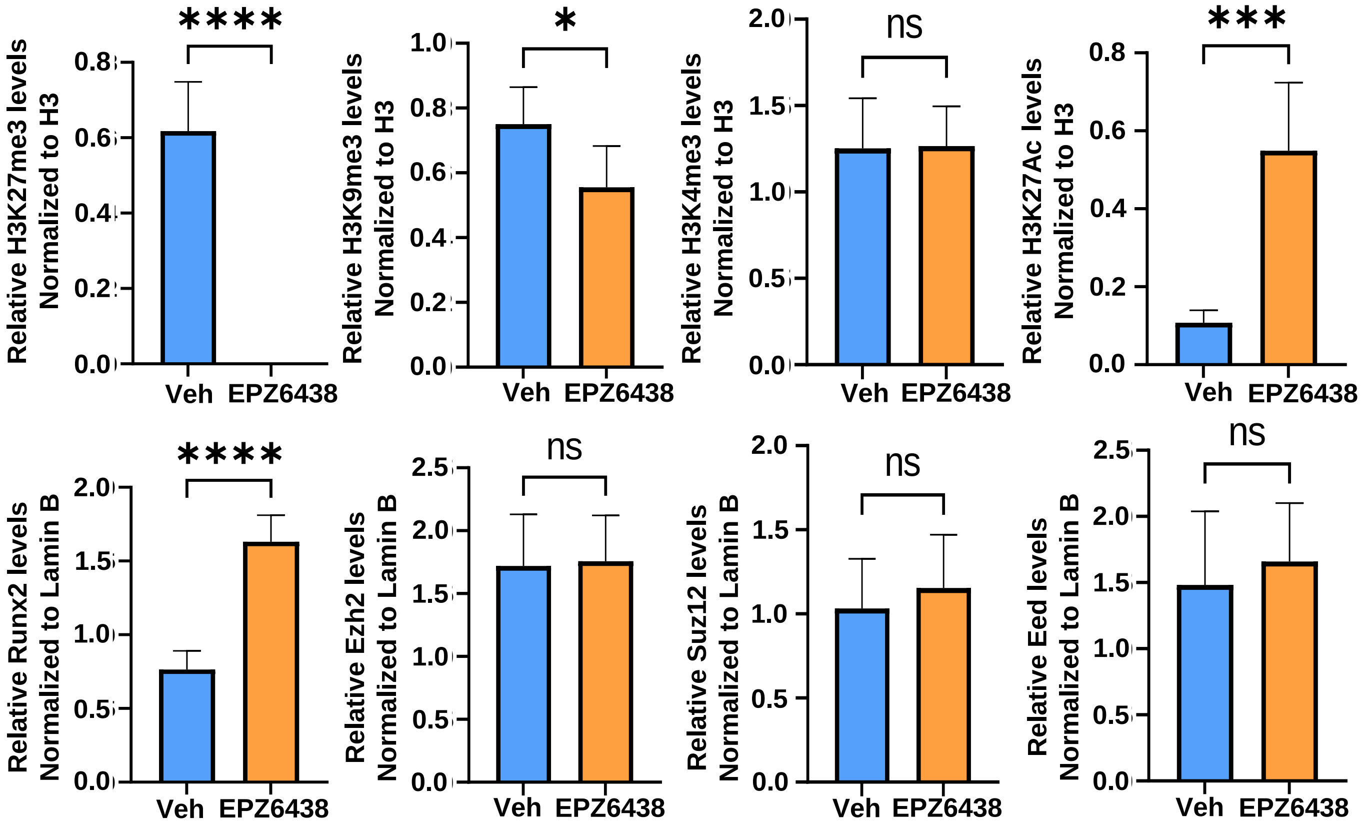

Figure S2. Western Blot quantification of Ezh2 inhibition by EPZ6438 in MC3T3 cells. Quantification and statistical analysis of protein bands shown in Figure 1C. All error bars represent mean ± SD of three experimental replicates. (ns = not significant, *= $p \leq 0.05$, **= $p \leq 0.01$, ***= $p \leq 0.001$, ****= $p \leq 0.0001$).

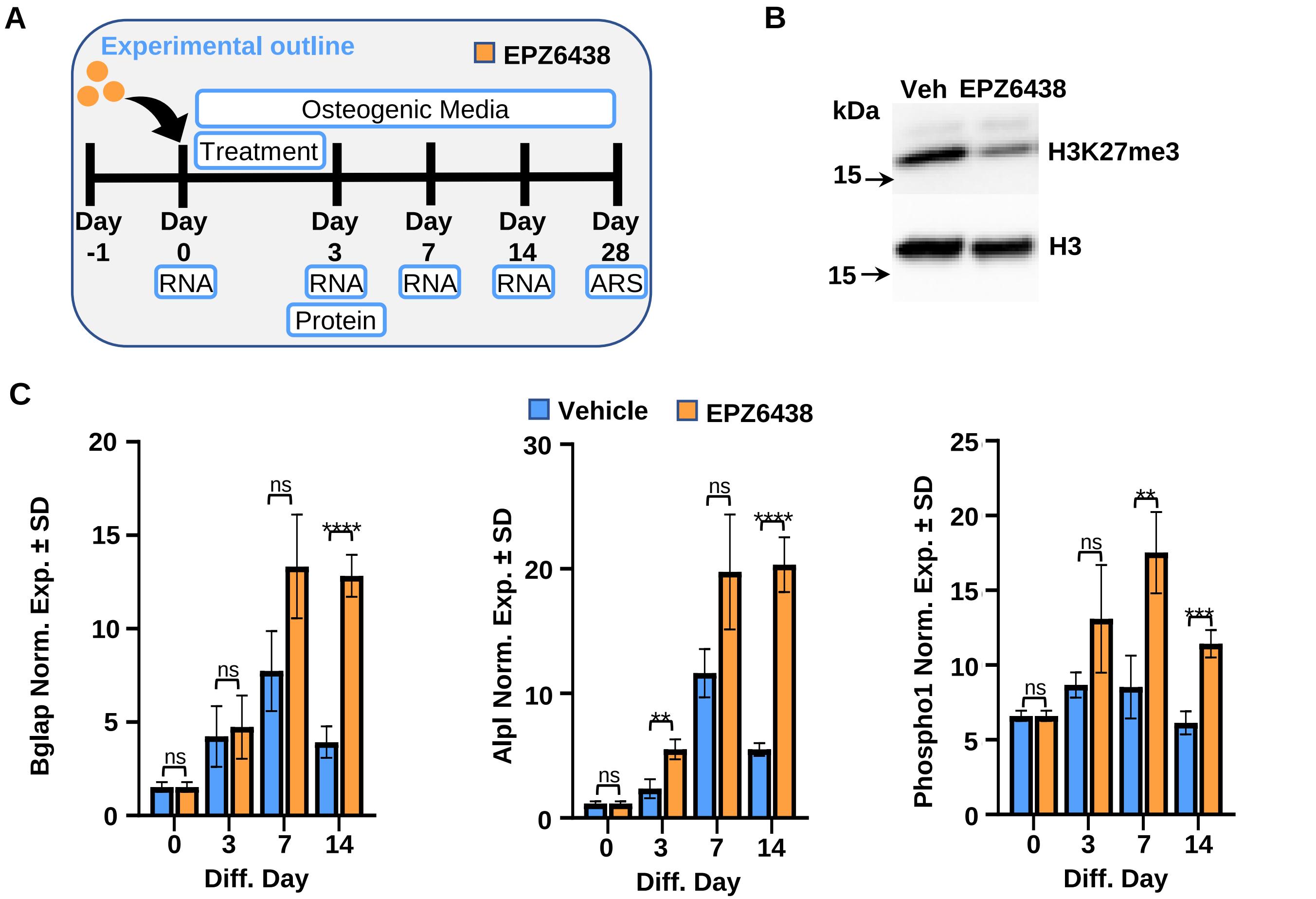

Figure S3. Ezh2 inhibition by EPZ6438 enhances osteogenic differentiation of primary mouse pre-osteoblast cells. Cells were treated with 2.5 μM EPZ6438 one day after plating and evaluated by Western blot and RT-qPCR analysis as shown (A). Western blotting analysis on day 3 (B). RT-qPCR analysis for bone-related markers relative to Gapdh (set at 100) as indicated time points (n = 3) (C). All error bars represent ± SD of three experimental replicates. (ns = not significant, *= p ≤ 0.05, **= p ≤ 0.01, ***= p ≤ 0.001, ****= p ≤ 0.0001).

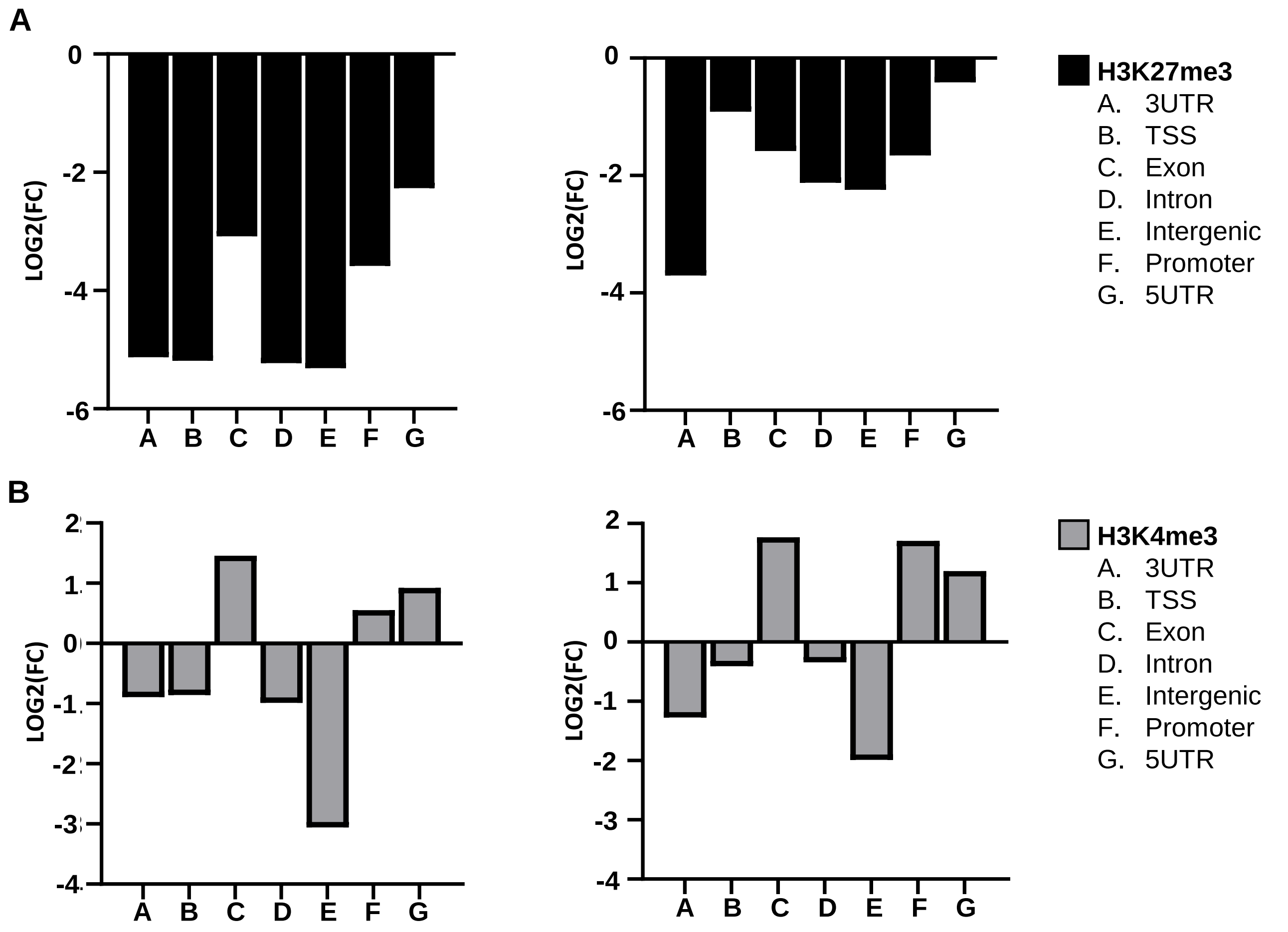

Figure S4. Graphical representation of the fold change (EPZ6438 versus Veh) of H3K27me3 (A) and H3K4me3 enrichment (B) in relationship with the genomic distribution. ChIP-Seq replicates for each modification are shown.

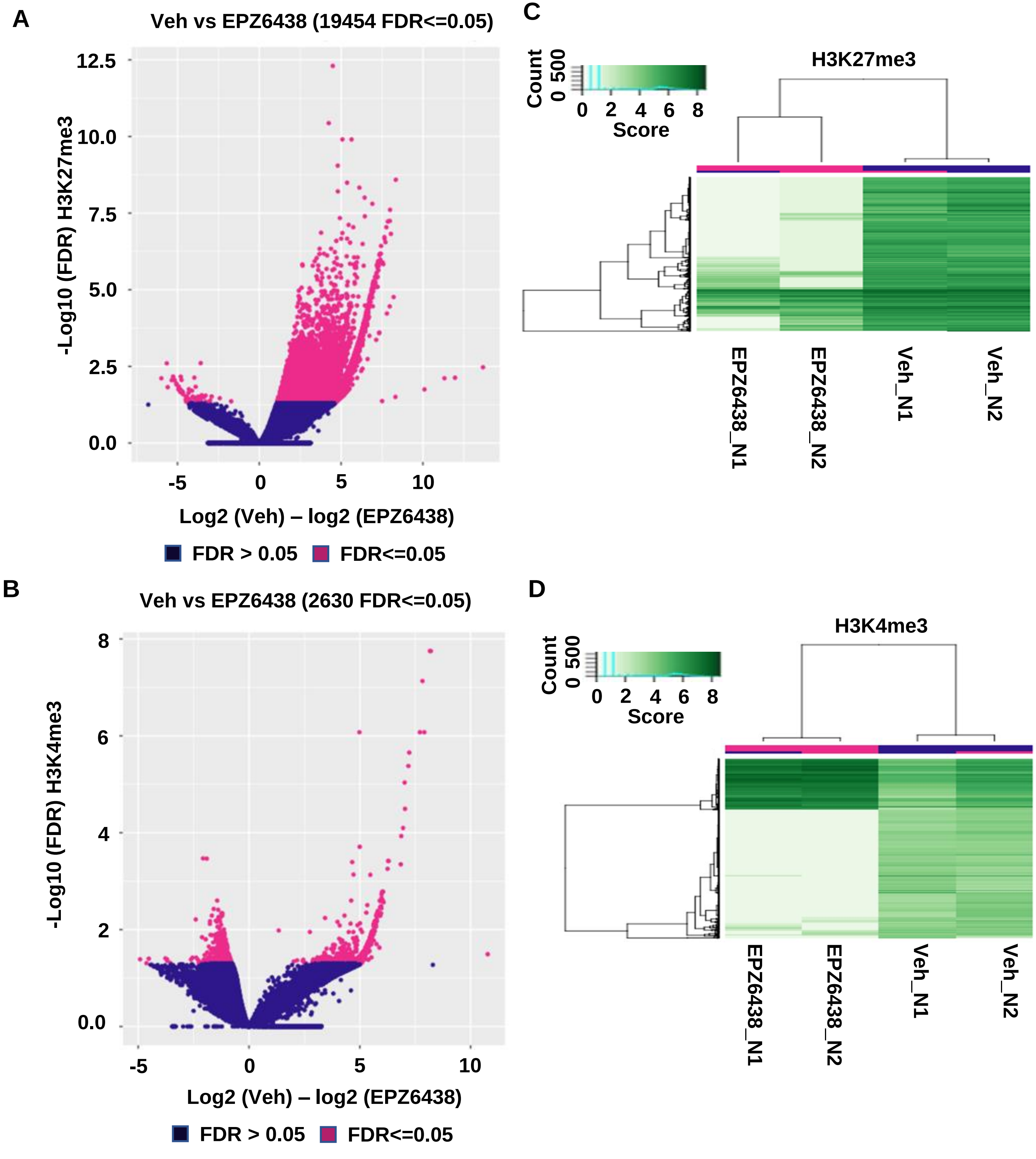
A
Veh vs EPZ6438 (19454 FDR<=0.05)
-Log10 (FDR) H3K27me3
Log2 (Veh) – log2 (EPZ6438)
FDR > 0.05
FDR<=0.05
B
Veh vs EPZ6438 (2630 FDR<=0.05)
-Log10 (FDR) H3K4me3
Log2 (Veh) – log2 (EPZ6438)
FDR > 0.05
FDR<=0.05
C
H3K27me3
Count
Score
EPZ6438_N1
EPZ6438_N2
Veh_N1
Veh_N2
D
H3K4me3
Count
Score
EPZ6438_N1
EPZ6438_N2
Veh_N1
Veh_N2

Figure S5. Differential binding analysis of H3K27me3 and H3K4me3 ChIP-seq peak data. Volcano plots highlight significantly differentially bound sites and show their fold changes and the confidence statistic (FDR or p-value) is shown on a negative log scale, helping visualize the relationship between the magnitude of fold changes and the confidence that sites are differentially bound for H3K27me3 (A) and H3K4me3 (B). Binding affinity heatmap showing affinities for differentially bound H3K27me3 (C) and H3K4me3 (D) sites. The figure shows the affinities and clustering of the differentially bound sites (rows), as well as the sample clustering (columns).

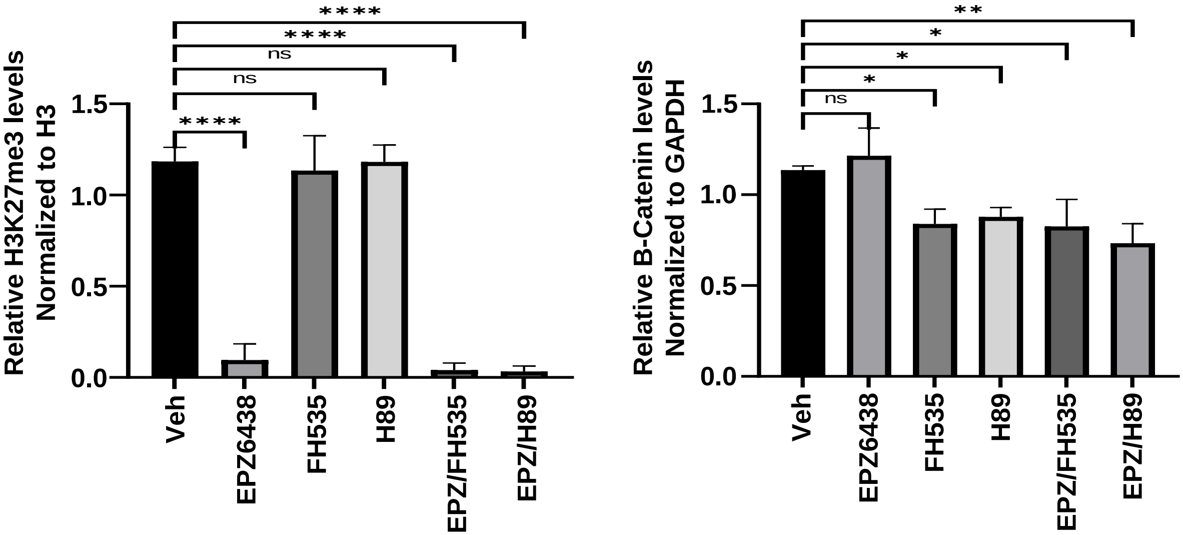

Figure S6. Western blot quantification of Ezh2 inhibition and disruption of WNT and cAMP pathway. Quantification and statistical analysis of relevant protein bands (H3K27me3 and B-catenin) shown in Figure 4B. All error bars represent ± SD of three experimental replicates. (ns = not significant, *= p ≤ 0.05, **= p ≤ 0.01, ***= p ≤ 0.001, ****= p ≤ 0.0001).

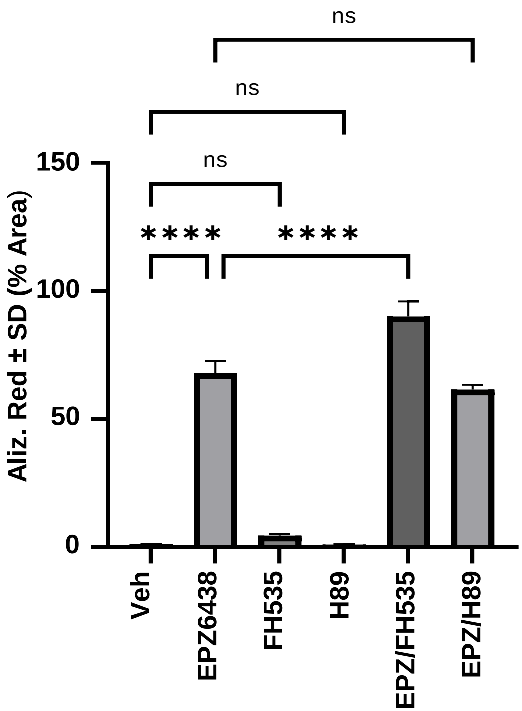

Figure S7. Alizarin Red quantification of pro-osteogenic effects induced by Ezh2 inhibition are enhanced by disruption of the WNT pathway. Quantification and statistical analysis of Alizarin red staining on day 32 (shown in Figure 4D). All error bars represent ± SD of three experimental replicates. (ns = not significant, *= p ≤ 0.05, **= p ≤ 0.01, ***= p ≤ 0.001, ****= p ≤ 0.0001).

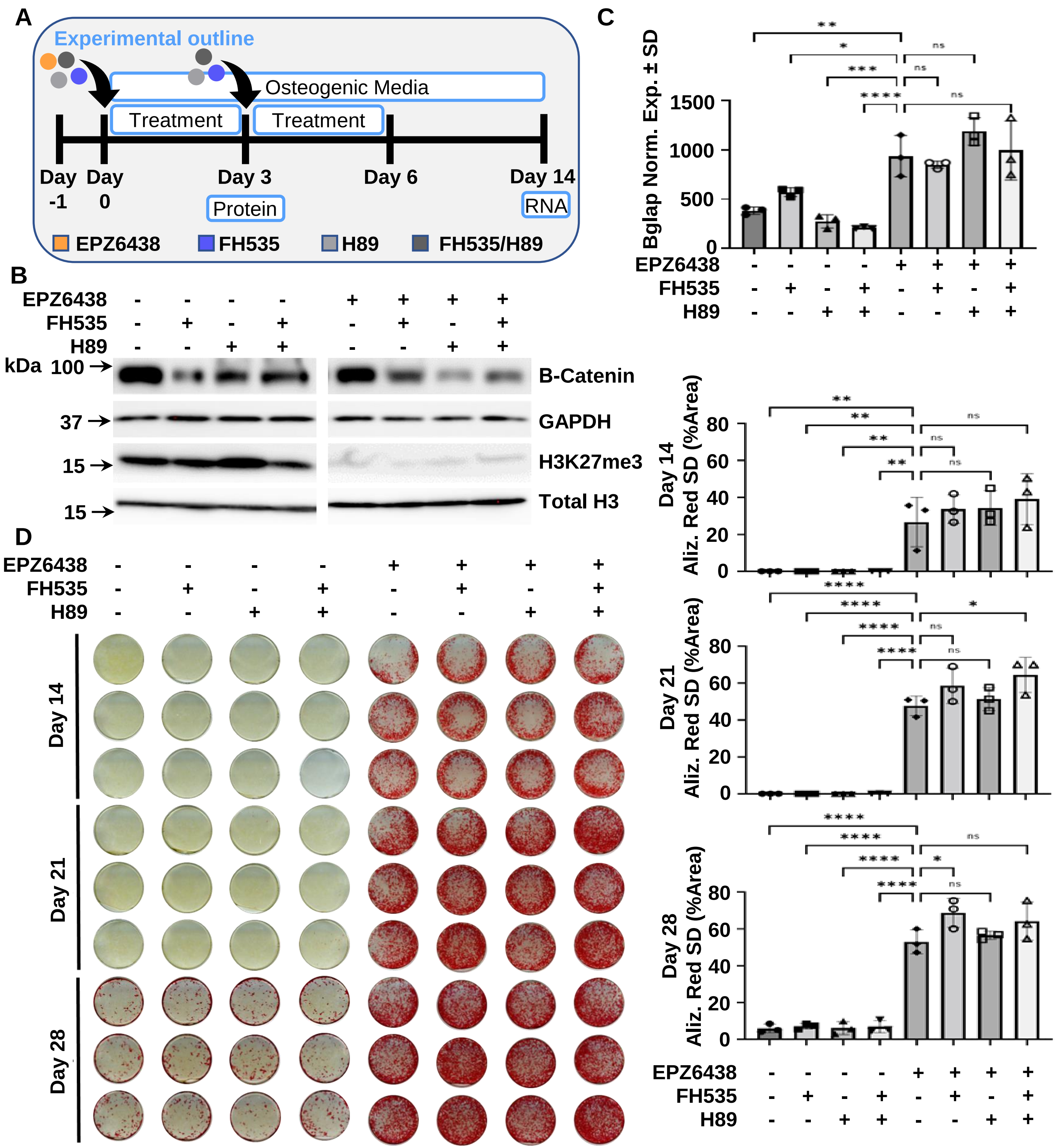

A
Experimental outline
Osteogenic Media
Treatment
Treatment
Day -1
Day 0
Day 3
Day 6
Day 14
Protein
RNA
EPZ6438
FH535
H89
FH535/H89
B
EPZ6438
FH535
H89
kDa 100
37
15
15
B-Catenin
GAPDH
H3K27me3
Total H3
C
Bglap Norm. Exp. ± SD
1500
1000
500
0
**
*
***
****
ns
ns
ns
D
EPZ6438
FH535
H89
Day 14
Day 21
Day 28
Day 14 Aliz. Red SD (%Area)
Day 21 Aliz. Red SD (%Area)
Day 28 Aliz. Red SD (%Area)
80
60
40
20
0

Figure S8. Osteogenic effects induced by Ezh2 inhibition are not changed by simultaneous disruption of WNT and cAMP pathway. Diagram of the experimental protocol for treatment of MC3T3 with vehicle (Veh) or 2.5 μM EPZ6438 with or without 5 μM FH535 or H89 shown in panels B-D (A). Western blotting analysis on day 3 (B). RT-qPCR analysis for bone-related markers relative to Gapdh (set at 100) on day 14 (C). Alizarin red staining on day 14, 21, 28 (D). All error bars represent ± SD of three experimental replicates. (ns = not significant, *= $p \leq 0.05$, **= $p \leq 0.01$, ***= $p \leq 0.001$, ****= $p \leq 0.0001$).

A

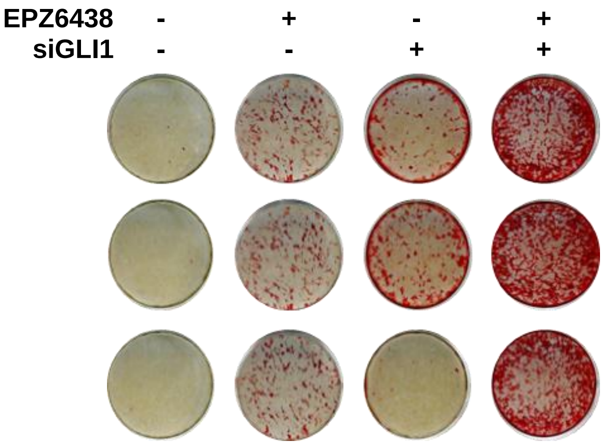

Figure S9. Silencing of Gli1 increases mineralization of MC3T3 pre-osteoblast. Diagram of the experimental protocol for treatment of MC3T3 siCtrl or siGli1 with vehicle (Veh) or 2.5 µM EPZ6438 is shown in Fig 5A. Alizarin red staining on day 26. All error bars represent ± SD of three experimental replicates. (ns = not significant, *= p ≤ 0.05, **= p ≤ 0.01, ***= p ≤ 0.001, ****= p ≤ 0.0001).

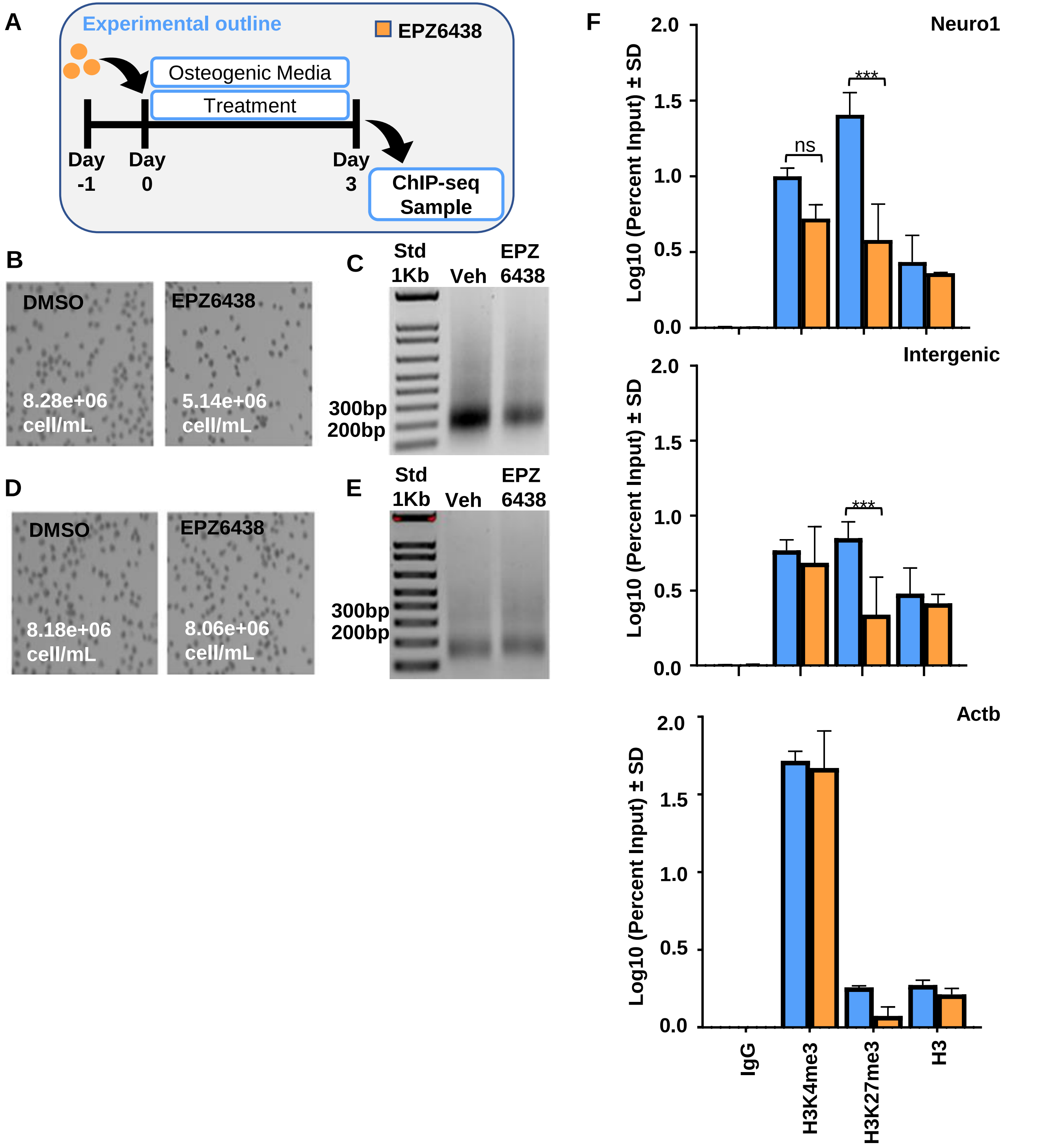

A
Experimental outline
EPZ6438
Osteogenic Media
Treatment
Day -1
Day 0
Day 3
ChIP-seq Sample
B
DMSO
8.28e+06 cell/mL
EPZ6438
5.14e+06 cell/mL
C
Std 1Kb
Veh
EPZ 6438
300bp
200bp
D
DMSO
8.18e+06 cell/mL
EPZ6438
8.06e+06 cell/mL
E
Std 1Kb
Veh
EPZ 6438
300bp
200bp
F
Neuro1
Intergenic
Actb
Log10 (Percent Input) ± SD
ns
***
IgG
H3K4me3
H3K27me3
H3

Figure S10. H3K27me3 and H3K4me3 ChIP-seq. Diagram of the experimental protocol for treatment of MC3T3 with vehicle (Veh) or 2.5 μM EPZ6438 shown in panel B-F (A). Cell numbers for the endpoint analysis and electrophoresis in 1% agarose gel to visualize chromatin shearing after sonication in first (B and C) and second (D and E) replicates. ChIP-qPCR analysis of Neuro1 promoter, an intragenic region, and Actb relative to Input (F). All error bars represent ± SD of two experimental replicates. (ns = not significant, *= $p \leq 0.05$, **= $p \leq 0.01$, ***= $p \leq 0.001$, ****= $p \leq 0.0001$).

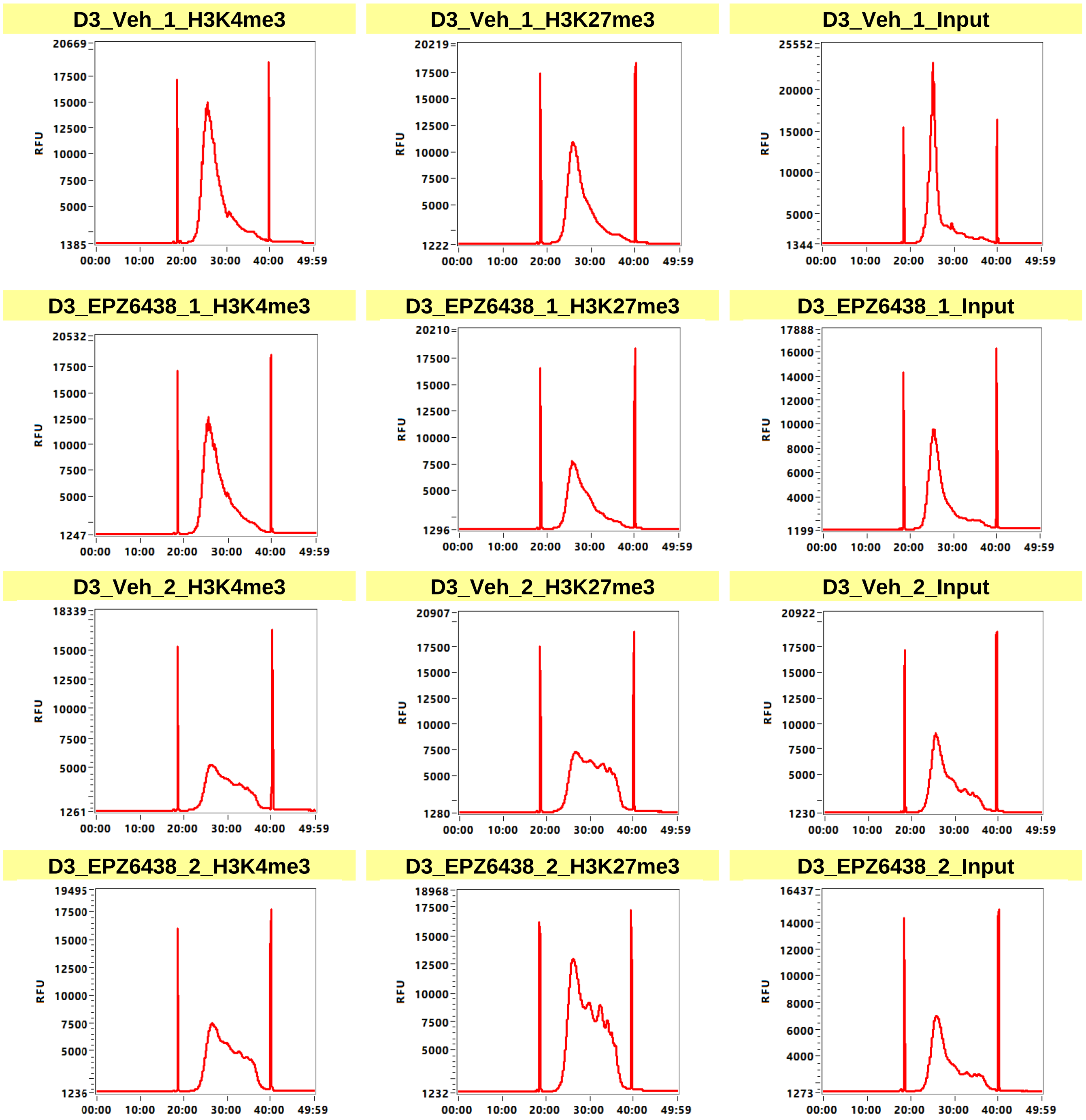

Figure S11. Representative electropherograms used for DNA fragmentation quality analysis of ChIP-seq samples on Fragment Analyzer.

A

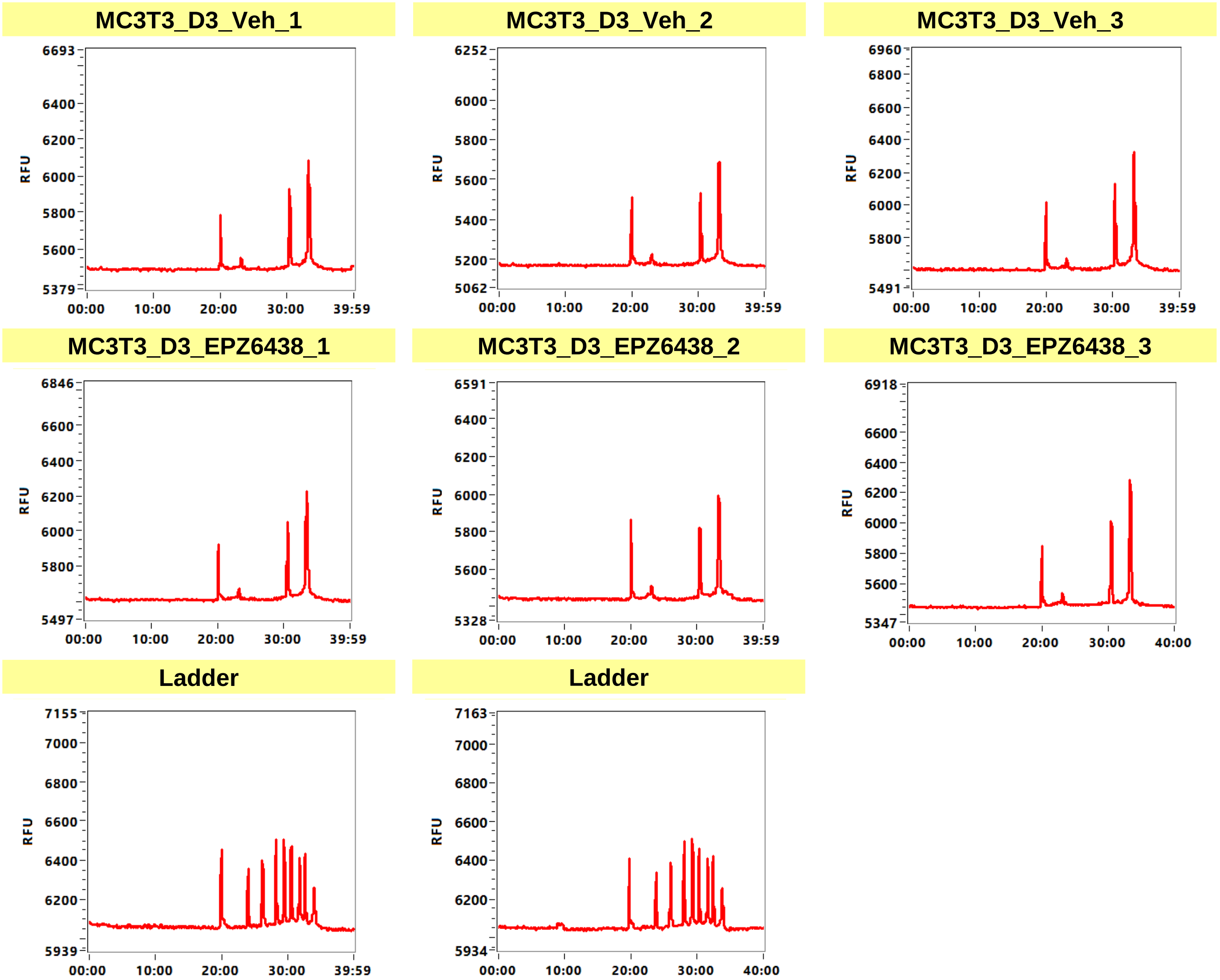

B

	Veh_1	Veh_2	Veh_3	EPZ6438_1	EPZ6438_2	EPZ6438_3
TIC	57.3434	38.3278	61.6541	51.7872	34.2948	54.5146
TIM	111.3040	81.0125	126.2136	111.0399	89.7076	130.1400
Conc.	72.3576	50.9668	88.9587	76.1101	48.5064	83.0863
28S/18S	1.9	1.9	1.8	1.8	1.8	1.9
RQN	10.0	10.0	9.8	9.7	10.0	9.2

Figure S12. Representative electropherograms used for RNA quality analysis of total RNA for each RNA-seq sample on Fragment Analyzer. The LM at 1 bp and UM at 6,000 bp are internal markers that are added to ensure proper sizing of samples. TIC: Total Integrated Concentration in ng/μL. Concentration of all detected peaks. TIM: Total Integrated Molarity in nmole/L. Molarity of all detected peaks. Total conc.: total concentration in ng/μL of sample. The RQN assists in assessing the presence or absence of degradation. RQN considers the entire electropherogram with scoring from 1 to 10, where 10 indicates the highest possible RNA quality.

A

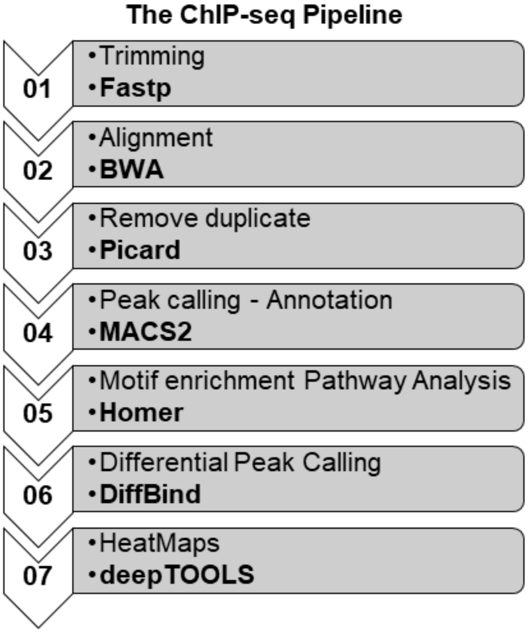

B

		# of ChIP-seq Reads		
**Treatment**	**Replicate**	**Input**	**H3K4me3**	**H3K27me3**
**DMSO**	#1	60,724,722	71,359,676	64,012,591
**DMSO**	#2	48,669,870	66,088,717	53,592,704
**EPZ6438**	#1	70,540,229	60,255,417	59,096,653
**EPZ6438**	#2	67,704,031	62,113,823	68,267,493

Figure S13. Schematic representation of ChIP-seq protocol (A). Number of ChIP-Seq reads for each antibody (H3K4me3 and H3K27me3) and input from two treatment groups (DMSO and 2.5 μM EPZ6438) and two biological replicates for each treatment group (B).

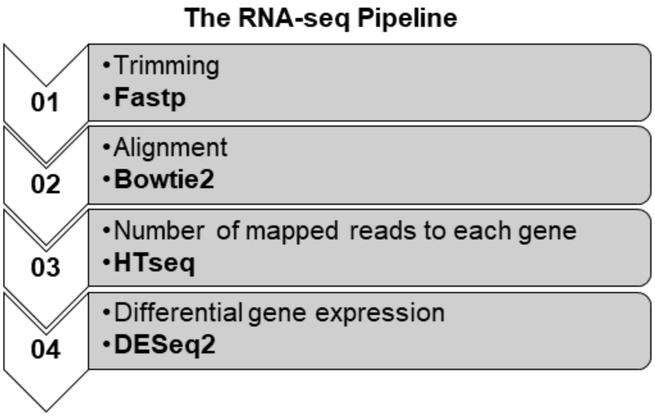

Figure S14. Schematic representation of RNA-seq protocol steps.

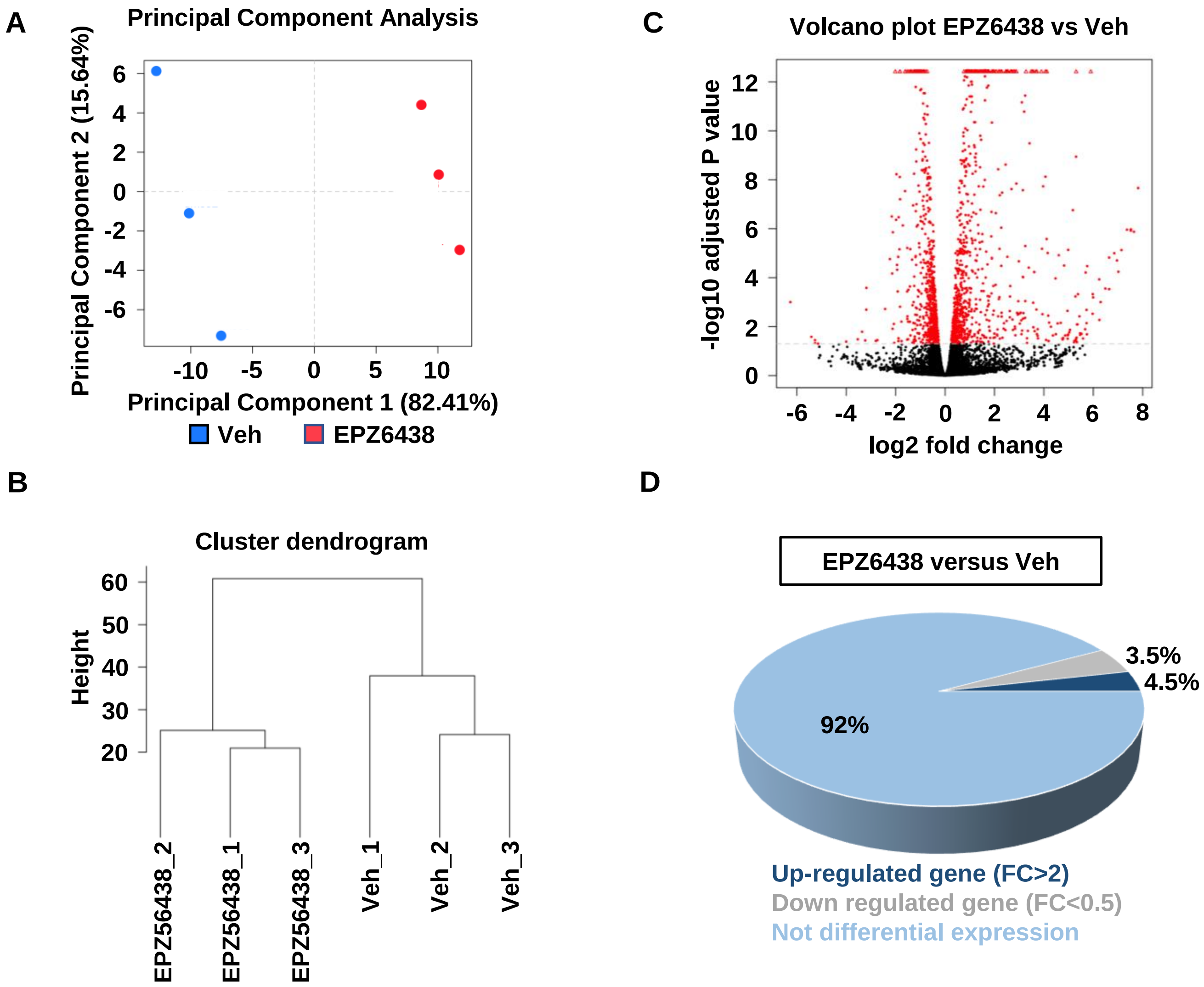

Figure S15. RNA-seq differential gene expression analysis using DESeq2. Principal component analysis (PCA) (A). Dendrogram of the RNA-seq samples (B). Volcano plot showing similar numbers of up and down-regulated genes (C). Graphic representation of the percentage of up and down regulated genes (D).

Table S1: EPZ6438-induced genes listed in figure 3E that exhibited change in gene expression (FC > 5) by RNA-seq that correlated with a reduction in H3K27me3 and presence or increase in H3K4me3 by K-means clustering.

FC>5										
FC>5, H3K27me3 cluster 1										
FC>5, H3K27me3 cluster 1, H3K4me3 cluster 2										
**Id**	**RNAseq**	**H3K27me3**	**H3K4me3**	**norm.dmso1**	**norm.dmso2**	**norm.dmso3**	**norm.epz1**	**norm.epz2**	**norm.epz3**	**FoldChange**
Colgalt2	FC > 5	cluster_1	cluster_2	0	0	0	45	21	35	183.249
Frat1	FC > 5	cluster_1	cluster_2	0	0	2	18	25	32	38.796
Lhx2	FC > 5	cluster_1	cluster_2	7	1	1	21	36	22	9.45
Wnt11	FC > 5	cluster_1	cluster_2	11	4	6	32	64	72	7.92
FC>5, H3K27me3 cluster 1, H3K4me3 cluster 3										
**Id**	**RNAseq**	**H3K27me3**	**H3K4me3**	**norm.dmso1**	**norm.dmso2**	**norm.dmso3**	**norm.epz1**	**norm.epz2**	**norm.epz3**	**FoldChange**
Adgrd1	FC > 5	cluster_1	cluster_3	418	331	400	2088	2114	2118	5.504
Asb4	FC > 5	cluster_1	cluster_3	1	4	0	24	43	54	24.287
Chn2	FC > 5	cluster_1	cluster_3	0	0	1	69	99	73	225.356
Cnksr1	FC > 5	cluster_1	cluster_3	1	1	0	17	16	3	18.271
Cryba2	FC > 5	cluster_1	cluster_3	0	2	2	13	23	23	15.356
Cyp17a1	FC > 5	cluster_1	cluster_3	1	0	0	6	28	10	41.151
E330013P04R	FC > 5	cluster_1	cluster_3	1	0	0	43	22	59	115.276
Enpp5	FC > 5	cluster_1	cluster_3	0	0	0	24	6	25	99.873
Epas1	FC > 5	cluster_1	cluster_3	5	23	17	130	138	162	9.462
Frmpd1	FC > 5	cluster_1	cluster_3	0	0	0	7	7	8	40.084
Hoxb13	FC > 5	cluster_1	cluster_3	3	4	1	96	108	111	39.577
Hpse	FC > 5	cluster_1	cluster_3	68	70	68	920	763	777	11.915
Ifnlr1	FC > 5	cluster_1	cluster_3	7	3	18	39	77	55	6.184
Ikzf4	FC > 5	cluster_1	cluster_3	0	0	0	21	13	15	89.724
Nsg2	FC > 5	cluster_1	cluster_3	0	0	0	6	13	5	44.202
Ooep	FC > 5	cluster_1	cluster_3	1	0	0	10	36	22	63.745
Prkch	FC > 5	cluster_1	cluster_3	7	13	1	87	41	51	8.89
Rufy4	FC > 5	cluster_1	cluster_3	0	0	0	10	1	15	47.952
Scn1a	FC > 5	cluster_1	cluster_3	0	0	0	24	21	24	124.3
Slc36a2	FC > 5	cluster_1	cluster_3	8	5	10	73	100	68	10.701
Slc6a4	FC > 5	cluster_1	cluster_3	22	22	15	265	230	261	13.102
Smtnl2	FC > 5	cluster_1	cluster_3	1	0	4	59	32	16	22.172
Steap3	FC > 5	cluster_1	cluster_3	12	31	45	349	358	300	11.164
Tbx4	FC > 5	cluster_1	cluster_3	2	1	4	37	39	42	17.294
Tlx1	FC > 5	cluster_1	cluster_3	5	1	4	25	22	38	9.489
Wfdc6a	FC > 5	cluster_1	cluster_3	1	0	1	14	11	9	17.044
FC>5, H3K27me3 cluster 1, H3K4me3 cluster 4										
**Id**	**RNAseq**	**H3K27me3**	**H3K4me3**	**norm.dmso1**	**norm.dmso2**	**norm.dmso3**	**norm.epz1**	**norm.epz2**	**norm.epz3**	**FoldChange**
5730559C18R	FC > 5	cluster_1	cluster_4	2	2	1	55	85	55	39.503
Car12	FC > 5	cluster_1	cluster_4	5	4	4	30	15	25	5.436
Dbh	FC > 5	cluster_1	cluster_4	3	5	3	43	37	11	8.334
Eln	FC > 5	cluster_1	cluster_4	3	34	22	320	503	245	17.813
Fam167a	FC > 5	cluster_1	cluster_4	6	27	8	205	218	267	16.732
Gng2	FC > 5	cluster_1	cluster_4	11	23	8	125	119	115	8.631
Matn1	FC > 5	cluster_1	cluster_4	0	1	0	17	12	4	31.39

Scrg1	FC > 5	cluster_1	cluster_4	14	0	9	35	39	37	5.011
Smoc2	FC > 5	cluster_1	cluster_4	32	9	11	125	103	150	7.395
Tgm5	FC > 5	cluster_1	cluster_4	0	0	0	9	19	1	53.429
Trp53i11	FC > 5	cluster_1	cluster_4	0	1	0	17	32	17	63.045
Zdhhc2	FC > 5	cluster_1	cluster_4	0	0	0	10	9	10	52.932
**FC>5, H3K27me3 cluster 2**										
**FC>5, H3K27me3 cluster 2, H3K4me3 cluster 2**										
**Id**	**RNAseq**	**H3K27me3**	**H3K4me3**	**norm.dmso1**	**norm.dmso2**	**norm.dmso3**	**norm.epz1**	**norm.epz2**	**norm.epz3**	**FoldChange**
Wif1	FC > 5	cluster_2	cluster_2	215	218	227	1672	1499	1512	7.087
**FC>5, H3K27me3 cluster 2, H3K4me3 cluster 3**										
**Id**	**RNAseq**	**H3K27me3**	**H3K4me3**	**norm.dmso1**	**norm.dmso2**	**norm.dmso3**	**norm.epz1**	**norm.epz2**	**norm.epz3**	**FoldChange**
Csrnp3	FC > 5	cluster_2	cluster_3	0	0	1	25	22	34	75.561
Ptgdr2	FC > 5	cluster_2	cluster_3	5	1	0	7	34	11	9.963
Ramp1	FC > 5	cluster_2	cluster_3	282	290	374	1673	1853	1717	5.536
Slc9a2	FC > 5	cluster_2	cluster_3	0	0	4	38	45	25	28.189
Vnn1	FC > 5	cluster_2	cluster_3	5	6	1	16	40	33	8.063
**FC>5, H3K27me3 cluster 2, H3K4me3 cluster 4**										
**Id**	**RNAseq**	**H3K27me3**	**H3K4me3**	**norm.dmso1**	**norm.dmso2**	**norm.dmso3**	**norm.epz1**	**norm.epz2**	**norm.epz3**	**FoldChange**
Acsm4	FC > 5	cluster_2	cluster_4	0	0	0	7	21	15	78.656
Adamtsl2	FC > 5	cluster_2	cluster_4	2	1	7	18	25	27	6.708
Agtr1a	FC > 5	cluster_2	cluster_4	2	1	1	20	15	13	11.91
Alpk3	FC > 5	cluster_2	cluster_4	0	1	0	11	20	2	31.235
B4galnt3	FC > 5	cluster_2	cluster_4	0	1	1	38	40	27	53.873
Bcan	FC > 5	cluster_2	cluster_4	1	12	8	29	61	40	6.152
Btla	FC > 5	cluster_2	cluster_4	0	0	1	12	8	15	33.288
Cabp1	FC > 5	cluster_2	cluster_4	1	6	4	33	29	29	7.799
Camk1g	FC > 5	cluster_2	cluster_4	6	0	0	24	29	13	12.324
Ccdc88c	FC > 5	cluster_2	cluster_4	1	2	11	53	44	61	10.465
Col22a1	FC > 5	cluster_2	cluster_4	1	2	1	27	40	4	18.08
Fam81a	FC > 5	cluster_2	cluster_4	20	3	1	112	139	139	16.743
Hhatl	FC > 5	cluster_2	cluster_4	0	0	0	1	13	27	75.925
Itga4	FC > 5	cluster_2	cluster_4	0	0	0	7	9	5	39.459
Klhl40	FC > 5	cluster_2	cluster_4	0	0	0	14	5	5	45.923
Ldb2	FC > 5	cluster_2	cluster_4	0	0	1	10	11	8	26.859
Myt1	FC > 5	cluster_2	cluster_4	0	0	0	2	13	7	40.331
Oprl1	FC > 5	cluster_2	cluster_4	9	0	1	38	29	46	11.853
Rab15	FC > 5	cluster_2	cluster_4	0	1	1	12	18	30	31.048
**FC>5, H3K27me3 cluster 3**										
**FC>5, H3K27me3 cluster 3, H3K4me3 cluster 1**										
**Id**	**RNAseq**	**H3K27me3**	**H3K4me3**	**norm.dmso1**	**norm.dmso2**	**norm.dmso3**	**norm.epz1**	**norm.epz2**	**norm.epz3**	**FoldChange**
Phospho1	FC > 5	cluster_3	cluster_1	368	353	459	2643	3045	3065	7.404
**FC>5, H3K27me3 cluster 3, H3K4me3 cluster 2**										
**Id**	**RNAseq**	**H3K27me3**	**H3K4me3**	**norm.dmso1**	**norm.dmso2**	**norm.dmso3**	**norm.epz1**	**norm.epz2**	**norm.epz3**	**FoldChange**
Ostn	FC > 5	cluster_3	cluster_2	0	0	0	32	46	23	183.567
**FC>5, H3K27me3 cluster 3, H3K4me3 cluster 3**										
**Id**	**RNAseq**	**H3K27me3**	**H3K4me3**	**norm.dmso1**	**norm.dmso2**	**norm.dmso3**	**norm.epz1**	**norm.epz2**	**norm.epz3**	**FoldChange**
Abi3	FC > 5	cluster_3	cluster_3	49	80	74	386	406	403	5.852
Mtus2	FC > 5	cluster_3	cluster_3	7	16	17	90	47	91	5.741
Prokr1	FC > 5	cluster_3	cluster_3	0	8	4	49	20	14	7.197
Rxfp3	FC > 5	cluster_3	cluster_3	0	2	9	43	53	41	12.159

FC>5, H3K27me3 cluster 3, H3K4me3 cluster 4										
**Id**	**RNAseq**	**H3K27me3**	**H3K4me3**	**norm.dmso1**	**norm.dmso2**	**norm.dmso3**	**norm.epz1**	**norm.epz2**	**norm.epz3**	**FoldChange**
4930594C11R	FC > 5	cluster_3	cluster_4	0	0	0	9	6	9	44.133
Acoxl	FC > 5	cluster_3	cluster_4	5	4	4	18	32	28	6.157
Adamts18	FC > 5	cluster_3	cluster_4	0	0	1	35	31	41	99.868
Adgrf4	FC > 5	cluster_3	cluster_4	0	0	0	13	8	13	62.785
Alx3	FC > 5	cluster_3	cluster_4	2	1	1	39	56	49	36.06
Aox3	FC > 5	cluster_3	cluster_4	0	0	4	25	17	9	13.417
Apcdd1	FC > 5	cluster_3	cluster_4	167	130	136	868	809	691	5.489
Atp1b2	FC > 5	cluster_3	cluster_4	0	0	1	26	24	7	52.957
Cbln1	FC > 5	cluster_3	cluster_4	1	0	0	5	11	13	27.108
Cd200	FC > 5	cluster_3	cluster_4	15	28	19	312	275	337	14.9
Cd300lb	FC > 5	cluster_3	cluster_4	0	0	1	6	8	20	31.579
Col2a1	FC > 5	cluster_3	cluster_4	2479	3655	4548	18933	26948	21675	6.323
Col9a1	FC > 5	cluster_3	cluster_4	0	0	4	39	52	30	31.672
Dio2	FC > 5	cluster_3	cluster_4	0	0	2	16	13	20	25.682
Fras1	FC > 5	cluster_3	cluster_4	0	0	4	16	13	21	13.157
Frem1	FC > 5	cluster_3	cluster_4	28	7	26	275	241	259	12.805
Frzb	FC > 5	cluster_3	cluster_4	0	0	1	26	93	57	164.674
Gap43	FC > 5	cluster_3	cluster_4	5	2	1	22	7	33	8.537
Gli2	FC > 5	cluster_3	cluster_4	0	0	0	14	54	41	200.062
Grhl3	FC > 5	cluster_3	cluster_4	8	18	11	86	142	107	9.211
Grrp1	FC > 5	cluster_3	cluster_4	0	0	1	18	18	10	43.349
H19	FC > 5	cluster_3	cluster_4	15	6	2	146	109	118	17.386
Hfe2	FC > 5	cluster_3	cluster_4	0	6	0	23	19	17	9.95
Klk14	FC > 5	cluster_3	cluster_4	5	2	4	21	31	24	7.55
Lama1	FC > 5	cluster_3	cluster_4	0	3	1	27	17	9	13.443
Lcp1	FC > 5	cluster_3	cluster_4	2	8	0	41	58	52	15.17
Lingo1	FC > 5	cluster_3	cluster_4	6	0	4	9	42	27	8.685
Lipg	FC > 5	cluster_3	cluster_4	1	3	7	69	53	60	15.659
Myzap	FC > 5	cluster_3	cluster_4	40	42	30	230	240	232	6.299
Penk	FC > 5	cluster_3	cluster_4	0	10	6	41	54	51	8.934
Prss16	FC > 5	cluster_3	cluster_4	6	1	5	47	13	24	7.098
Ptprr	FC > 5	cluster_3	cluster_4	0	0	0	5	16	7	50.905
Ptpru	FC > 5	cluster_3	cluster_4	1	5	2	26	22	23	9.018
Rab11fip4	FC > 5	cluster_3	cluster_4	5	5	5	32	58	50	9.494
Rapgef4	FC > 5	cluster_3	cluster_4	1	0	1	26	50	26	51.615
Slc8a3	FC > 5	cluster_3	cluster_4	182	174	158	1918	2108	1917	11.596
Srgap1	FC > 5	cluster_3	cluster_4	12	1	1	35	55	23	8.145
Stox2	FC > 5	cluster_3	cluster_4	0	2	0	23	16	5	22.404
Tppp	FC > 5	cluster_3	cluster_4	5	4	7	17	31	39	5.625
Tspan12	FC > 5	cluster_3	cluster_4	3	3	2	29	29	40	12.229
Wdr72	FC > 5	cluster_3	cluster_4	0	1	1	30	39	12	41.669
**FC>5, H3K27me3 cluster 4**										
**FC>5, H3K27me3 cluster 4, H3K4me3 cluster 3**										
**Id**	**RNAseq**	**H3K27me3**	**H3K4me3**	**norm.dmso1**	**norm.dmso2**	**norm.dmso3**	**norm.epz1**	**norm.epz2**	**norm.epz3**	**FoldChange**
Col11a2	FC > 5	cluster_4	cluster_3	101	212	296	1232	2555	1634	8.882
Rcsd1	FC > 5	cluster_4	cluster_3	7	6	0	18	41	28	7.125

FC>5, H3K27me3 cluster 4, H3K4me3 cluster 4										
**Id**	**RNAseq**	**H3K27me3**	**H3K4me3**	**norm.dmso1**	**norm.dmso2**	**norm.dmso3**	**norm.epz1**	**norm.epz2**	**norm.epz3**	**FoldChange**
1700017G19R	FC > 5	cluster_4	cluster_4	1	0	0	8	8	13	27.432
2610027K06R	FC > 5	cluster_4	cluster_4	1	4	3	12	26	32	8.942
C1qtnf3	FC > 5	cluster_4	cluster_4	316	195	220	1682	1548	1480	6.471
Car3	FC > 5	cluster_4	cluster_4	225	265	264	1698	1634	1747	6.725
Crybb1	FC > 5	cluster_4	cluster_4	0	0	1	6	26	11	40.471
D030025E07R	FC > 5	cluster_4	cluster_4	0	0	0	22	30	26	141.044
Fibin	FC > 5	cluster_4	cluster_4	0	0	0	5	9	7	37.689
Galnt3	FC > 5	cluster_4	cluster_4	24	11	52	183	201	179	6.447
Galnt5	FC > 5	cluster_4	cluster_4	0	0	1	12	9	10	29.093
Hdac9	FC > 5	cluster_4	cluster_4	14	3	8	40	48	63	6.231
Hecw2	FC > 5	cluster_4	cluster_4	47	17	43	202	229	149	5.467
Ifitm5	FC > 5	cluster_4	cluster_4	166	182	236	1595	2158	1914	9.677
Klhl30	FC > 5	cluster_4	cluster_4	51	84	89	404	449	415	5.618
Krt33b	FC > 5	cluster_4	cluster_4	1	12	8	45	36	29	5.219
Lhx4	FC > 5	cluster_4	cluster_4	0	0	1	18	10	7	32.671
Lhx9	FC > 5	cluster_4	cluster_4	7	4	8	13	38	46	5.171
Ncf1	FC > 5	cluster_4	cluster_4	56	55	56	311	349	287	5.71
Nrgn	FC > 5	cluster_4	cluster_4	1	0	1	15	17	14	23.498
Pdzd2	FC > 5	cluster_4	cluster_4	117	141	183	1045	1069	1027	7.086
Prex1	FC > 5	cluster_4	cluster_4	3	1	4	128	231	170	59.75
Prss35	FC > 5	cluster_4	cluster_4	67	104	102	476	497	535	5.495
Rorc	FC > 5	cluster_4	cluster_4	0	1	2	15	9	29	18.514
Shroom2	FC > 5	cluster_4	cluster_4	0	0	0	9	4	9	39.174
Slc6a15	FC > 5	cluster_4	cluster_4	1	0	4	20	32	23	12.976
Smpd3	FC > 5	cluster_4	cluster_4	1897	2561	3045	15884	19095	17678	7.015
Syt8	FC > 5	cluster_4	cluster_4	3	4	1	14	28	26	8.49
Tmem74	FC > 5	cluster_4	cluster_4	1	4	5	30	17	35	7.677
Tpd52	FC > 5	cluster_4	cluster_4	19	23	46	212	214	197	7.026
Trpm1	FC > 5	cluster_4	cluster_4	1	0	2	14	19	7	13.575
Trpm5	FC > 5	cluster_4	cluster_4	0	7	7	30	32	33	6.624

Table S2: EPZ6438-induced genes listed in figure 3E that exhibited change in gene expression (5 < FC > 2) by RNA-seq that correlated with a reduction in H3K27me3 and presence or increase in H3K4me3 by K-means clustering.

5<FC>2										
**5<FC>2, H3K27me3 cluster 1**										
**5<FC>2, H3K27me3 cluster 1, H3K4me3 cluster 2**										
**Id**	**RNAseq**	**H3K27me3**	**H3K4me3**	**norm.dmso1**	**norm.dmso2**	**norm.dmso3**	**norm.epz1**	**norm.epz2**	**norm.epz3**	**FoldChange**
Dlk2	5 < FC > 2	cluster_1	cluster_2	64	109	133	176	291	232	2.278
Neurl1a	5 < FC > 2	cluster_1	cluster_2	191	236	292	546	779	656	2.751
Scube3	5 < FC > 2	cluster_1	cluster_2	114	147	177	316	451	340	2.526
**5<FC>2, H3K27me3 cluster 1, H3K4me3 cluster 3**										
**Id**	**RNAseq**	**H3K27me3**	**H3K4me3**	**norm.dmso1**	**norm.dmso2**	**norm.dmso3**	**norm.epz1**	**norm.epz2**	**norm.epz3**	**FoldChange**
Aldh1l1	5 < FC > 2	cluster_1	cluster_3	26	26	32	108	90	80	3.289
Cracr2a	5 < FC > 2	cluster_1	cluster_3	86	63	108	170	172	271	2.377
Dync1i1	5 < FC > 2	cluster_1	cluster_3	22	13	15	64	54	40	3.222
Gata2	5 < FC > 2	cluster_1	cluster_3	36	49	106	135	159	146	2.289
Ggt7	5 < FC > 2	cluster_1	cluster_3	70	133	178	255	276	289	2.14
Notum	5 < FC > 2	cluster_1	cluster_3	72	104	135	250	311	262	2.645
Pacsin1	5 < FC > 2	cluster_1	cluster_3	18	9	17	53	37	61	3.456
Pcp4l1	5 < FC > 2	cluster_1	cluster_3	3	18	10	50	34	67	4.883
Plcg2	5 < FC > 2	cluster_1	cluster_3	72	92	111	190	255	234	2.462
Ppfia4	5 < FC > 2	cluster_1	cluster_3	1	9	16	38	35	30	3.927
Ptprv	5 < FC > 2	cluster_1	cluster_3	1544	2026	2429	4139	5311	4310	2.293
Scn4a	5 < FC > 2	cluster_1	cluster_3	43	54	56	176	70	103	2.28
Slc25a23	5 < FC > 2	cluster_1	cluster_3	82	76	88	192	174	214	2.352
Slc35g1	5 < FC > 2	cluster_1	cluster_3	45	51	62	155	138	159	2.846
Sstr2	5 < FC > 2	cluster_1	cluster_3	8	13	5	34	34	47	4.43
Tnfrsf21	5 < FC > 2	cluster_1	cluster_3	100	93	115	170	232	227	2.047
**5<FC>2, H3K27me3 cluster 1, H3K4me3 cluster 4**										
**Id**	**RNAseq**	**H3K27me3**	**H3K4me3**	**norm.dmso1**	**norm.dmso2**	**norm.dmso3**	**norm.epz1**	**norm.epz2**	**norm.epz3**	**FoldChange**
Atp2a3	5 < FC > 2	cluster_1	cluster_4	28	22	44	111	146	93	3.716
Cdh22	5 < FC > 2	cluster_1	cluster_4	3	13	20	30	97	33	4.33
Myl10	5 < FC > 2	cluster_1	cluster_4	84	87	55	174	170	228	2.541
Rhbdf2	5 < FC > 2	cluster_1	cluster_4	41	45	69	107	126	134	2.36
Tspan18	5 < FC > 2	cluster_1	cluster_4	241	314	312	771	937	846	2.942
**5<FC>2, H3K27me3 cluster 2**										
**5<FC>2, H3K27me3 cluster 2, H3K4me3 cluster 1**										
**Id**	**RNAseq**	**H3K27me3**	**H3K4me3**	**norm.dmso1**	**norm.dmso2**	**norm.dmso3**	**norm.epz1**	**norm.epz2**	**norm.epz3**	**FoldChange**
Chd7	5 < FC > 2	cluster_2	cluster_1	435	506	541	1024	1019	1090	2.111
**5<FC>2, H3K27me3 cluster 2, H3K4me3 cluster 2**										
**Id**	**RNAseq**	**H3K27me3**	**H3K4me3**	**norm.dmso1**	**norm.dmso2**	**norm.dmso3**	**norm.epz1**	**norm.epz2**	**norm.epz3**	**FoldChange**
Ak4	5 < FC > 2	cluster_2	cluster_2	203	132	177	435	317	339	2.131
Chst11	5 < FC > 2	cluster_2	cluster_2	984	845	687	2489	2212	2317	2.791
Cxcl12	5 < FC > 2	cluster_2	cluster_2	1502	1270	1388	2847	2931	2946	2.098
Lims2	5 < FC > 2	cluster_2	cluster_2	7	21	21	38	52	58	3.009
Rhou	5 < FC > 2	cluster_2	cluster_2	647	536	565	1333	1124	1150	2.064
Rhpn2	5 < FC > 2	cluster_2	cluster_2	127	95	89	366	332	360	3.415
Sgk3	5 < FC > 2	cluster_2	cluster_2	143	114	92	268	273	271	2.333

Tbx2	5 < FC > 2	cluster_2	cluster_2	127	91	161	462	657	617	4.568
Wnt6	5 < FC > 2	cluster_2	cluster_2	352	375	438	779	1002	857	2.263
**5<FC>2, H3K27me3 cluster 2, H3K4me3 cluster 3**										
**Id**	**RNAseq**	**H3K27me3**	**H3K4me3**	**norm.dmso1**	**norm.dmso2**	**norm.dmso3**	**norm.epz1**	**norm.epz2**	**norm.epz3**	**FoldChange**
1110017D15R	5 < FC > 2	cluster_2	cluster_3	15	11	25	41	42	53	2.698
4833417C18R	5 < FC > 2	cluster_2	cluster_3	41	28	16	82	57	64	2.401
Aif1l	5 < FC > 2	cluster_2	cluster_3	188	175	144	342	310	367	2.013
Dlx3	5 < FC > 2	cluster_2	cluster_3	87	74	95	337	327	328	3.858
Itga9	5 < FC > 2	cluster_2	cluster_3	559	667	653	1333	1300	1324	2.104
Mfap4	5 < FC > 2	cluster_2	cluster_3	91	52	97	270	312	311	3.726
Myom1	5 < FC > 2	cluster_2	cluster_3	563	568	571	1781	1634	1662	2.98
Nlrc5	5 < FC > 2	cluster_2	cluster_3	92	107	98	199	309	208	2.415
Osr2	5 < FC > 2	cluster_2	cluster_3	33	50	56	66	120	103	2.069
Pde5a	5 < FC > 2	cluster_2	cluster_3	16	30	15	39	57	70	2.709
Pgm2l1	5 < FC > 2	cluster_2	cluster_3	207	256	168	454	397	509	2.154
Plagl1	5 < FC > 2	cluster_2	cluster_3	5823	6564	6223	13321	13501	13104	2.145
Wnt4	5 < FC > 2	cluster_2	cluster_3	136	161	240	335	530	395	2.338
**5<FC>2, H3K27me3 cluster 2, H3K4me3 cluster 4**										
**Id**	**RNAseq**	**H3K27me3**	**H3K4me3**	**norm.dmso1**	**norm.dmso2**	**norm.dmso3**	**norm.epz1**	**norm.epz2**	**norm.epz3**	**FoldChange**
C4b	5 < FC > 2	cluster_2	cluster_4	9	13	13	44	31	28	2.921
Camk4	5 < FC > 2	cluster_2	cluster_4	106	80	107	346	276	325	3.237
Extl1	5 < FC > 2	cluster_2	cluster_4	6	13	21	48	59	77	4.591
Greb1	5 < FC > 2	cluster_2	cluster_4	9	6	7	44	19	33	4.356
Igf2	5 < FC > 2	cluster_2	cluster_4	212	200	217	407	396	522	2.105
Inhbb	5 < FC > 2	cluster_2	cluster_4	76	53	101	121	187	163	2.046
Lif	5 < FC > 2	cluster_2	cluster_4	186	113	145	284	321	286	2.016
Mab21l3	5 < FC > 2	cluster_2	cluster_4	61	69	69	165	181	164	2.556
Map3k13	5 < FC > 2	cluster_2	cluster_4	26	24	22	109	69	91	3.711
Nhsl1	5 < FC > 2	cluster_2	cluster_4	1627	1674	1727	4489	4760	4896	2.812
Pcsk6	5 < FC > 2	cluster_2	cluster_4	694	624	786	2008	2375	2155	3.106
Twist2	5 < FC > 2	cluster_2	cluster_4	18	23	25	61	61	40	2.47
Vipr2	5 < FC > 2	cluster_2	cluster_4	22	13	4	41	48	43	3.552
Wnt7b	5 < FC > 2	cluster_2	cluster_4	41	24	30	87	92	117	3.13
**5<FC>2, H3K27me3 cluster 3**										
**5<FC>2, H3K27me3 cluster 3, H3K4me3 cluster 1**										
**Id**	**RNAseq**	**H3K27me3**	**H3K4me3**	**norm.dmso1**	**norm.dmso2**	**norm.dmso3**	**norm.epz1**	**norm.epz2**	**norm.epz3**	**FoldChange**
Nr4a3	5 < FC > 2	cluster_3	cluster_1	121	92	77	305	336	330	3.363
Zfp618	5 < FC > 2	cluster_3	cluster_1	41	71	74	117	219	207	2.9
**5<FC>2, H3K27me3 cluster 3, H3K4me3 cluster 2**										
**Id**	**RNAseq**	**H3K27me3**	**H3K4me3**	**norm.dmso1**	**norm.dmso2**	**norm.dmso3**	**norm.epz1**	**norm.epz2**	**norm.epz3**	**FoldChange**
Hectd2	5 < FC > 2	cluster_3	cluster_2	271	238	189	544	519	562	2.333
Itga6	5 < FC > 2	cluster_3	cluster_2	10	34	11	74	42	76	3.479
Pde1b	5 < FC > 2	cluster_3	cluster_2	174	176	158	382	362	427	2.308
Tmod1	5 < FC > 2	cluster_3	cluster_2	192	160	232	526	566	546	2.799
**5<FC>2, H3K27me3 cluster 3, H3K4me3 cluster 3**										
**Id**	**RNAseq**	**H3K27me3**	**H3K4me3**	**norm.dmso1**	**norm.dmso2**	**norm.dmso3**	**norm.epz1**	**norm.epz2**	**norm.epz3**	**FoldChange**
Cd244	5 < FC > 2	cluster_3	cluster_3	25	18	27	60	64	60	2.616
Ckmt1	5 < FC > 2	cluster_3	cluster_3	81	41	67	132	120	148	2.126
Creb3l1	5 < FC > 2	cluster_3	cluster_3	4352	5987	7196	11504	13788	12683	2.165
Dock8	5 < FC > 2	cluster_3	cluster_3	137	80	180	344	351	369	2.671

Efna3	5 < FC > 2	cluster_3	cluster_3	61	75	81	154	206	259	2.834
Mob3b	5 < FC > 2	cluster_3	cluster_3	377	386	333	825	745	840	2.198
Osbpl10	5 < FC > 2	cluster_3	cluster_3	20	42	38	82	83	111	2.724
Plekhh1	5 < FC > 2	cluster_3	cluster_3	14	42	35	165	149	141	4.96
Rasgrp2	5 < FC > 2	cluster_3	cluster_3	11	24	20	57	77	33	2.972
Sema4a	5 < FC > 2	cluster_3	cluster_3	11	3	4	17	33	30	4.656

5<FC>2, H3K27me3 cluster 3, H3K4me3 cluster 4

Id	RNAseq	H3K27me3	H3K4me3	norm.dmso1	norm.dmso2	norm.dmso3	norm.epz1	norm.epz2	norm.epz3	FoldChange
3425401B19R	5 < FC > 2	cluster_3	cluster_4	284	377	347	1139	1112	947	3.172
Acot1	5 < FC > 2	cluster_3	cluster_4	17	20	22	84	49	65	3.372
Adamts17	5 < FC > 2	cluster_3	cluster_4	2	15	10	25	36	55	4.295
Arl4c	5 < FC > 2	cluster_3	cluster_4	450	360	265	1603	1471	1402	4.174
Chst3	5 < FC > 2	cluster_3	cluster_4	175	197	197	451	362	399	2.128
Cited1	5 < FC > 2	cluster_3	cluster_4	47	67	48	88	165	91	2.136
Clstn3	5 < FC > 2	cluster_3	cluster_4	19	12	22	47	29	76	2.852
Cmya5	5 < FC > 2	cluster_3	cluster_4	93	84	93	257	226	240	2.67
Col23a1	5 < FC > 2	cluster_3	cluster_4	6	37	50	100	161	165	4.526
Coro2b	5 < FC > 2	cluster_3	cluster_4	59	84	67	203	196	216	2.924
Cpxm1	5 < FC > 2	cluster_3	cluster_4	30	39	20	66	98	60	2.522
Cyp26b1	5 < FC > 2	cluster_3	cluster_4	1567	1930	1935	5522	5861	5823	3.166
Dlg2	5 < FC > 2	cluster_3	cluster_4	26	23	27	78	74	76	2.957
Dusp27	5 < FC > 2	cluster_3	cluster_4	508	497	579	1038	1194	1045	2.068
Efr3b	5 < FC > 2	cluster_3	cluster_4	17	34	18	77	94	58	3.32
Fgd3	5 < FC > 2	cluster_3	cluster_4	357	430	436	1120	1299	1222	2.974
Gbgt1	5 < FC > 2	cluster_3	cluster_4	16	18	24	60	46	39	2.507
Gimap6	5 < FC > 2	cluster_3	cluster_4	26	10	18	48	53	50	2.845
Itpk1	5 < FC > 2	cluster_3	cluster_4	72	77	117	160	212	185	2.085
Khdrbs3	5 < FC > 2	cluster_3	cluster_4	56	22	57	99	94	102	2.194
Lax1	5 < FC > 2	cluster_3	cluster_4	18	26	9	45	39	67	2.838
Mdga1	5 < FC > 2	cluster_3	cluster_4	647	723	901	1459	1574	1587	2.031
Mmel1	5 < FC > 2	cluster_3	cluster_4	12	29	16	76	84	64	3.877
Mmp15	5 < FC > 2	cluster_3	cluster_4	18	33	50	69	107	85	2.541
Ncald	5 < FC > 2	cluster_3	cluster_4	70	31	73	139	178	150	2.668
Padi2	5 < FC > 2	cluster_3	cluster_4	198	155	242	952	815	966	4.596
Pde1a	5 < FC > 2	cluster_3	cluster_4	42	84	45	145	141	132	2.435
Phf19	5 < FC > 2	cluster_3	cluster_4	45	72	70	146	163	154	2.461
Rap1gap2	5 < FC > 2	cluster_3	cluster_4	18	10	10	37	29	53	3.182
Sox13	5 < FC > 2	cluster_3	cluster_4	30	30	48	114	166	118	3.66
Tec	5 < FC > 2	cluster_3	cluster_4	3	7	18	41	28	42	3.911
Tmem229b	5 < FC > 2	cluster_3	cluster_4	261	337	329	1061	943	1135	3.381
Tmem56	5 < FC > 2	cluster_3	cluster_4	250	260	182	865	672	684	3.216
Tns1	5 < FC > 2	cluster_3	cluster_4	2320	2879	3103	5645	6502	5752	2.155
Zfp467	5 < FC > 2	cluster_3	cluster_4	12	15	34	101	70	87	4.131

5<FC>2, H3K27me3 cluster 4

5<FC>2, H3K27me3 cluster 4, H3K4me3 cluster 1

Id	RNAseq	H3K27me3	H3K4me3	norm.dmso1	norm.dmso2	norm.dmso3	norm.epz1	norm.epz2	norm.epz3	FoldChange
Rcor2	5 < FC > 2	cluster_4	cluster_1	1164	1427	1650	3908	4957	4320	3.108

5<FC>2, H3K27me3 cluster 4, H3K4me3 cluster 2

Id	RNAseq	H3K27me3	H3K4me3	norm.dmso1	norm.dmso2	norm.dmso3	norm.epz1	norm.epz2	norm.epz3	FoldChange
Asxl3	5 < FC > 2	cluster_4	cluster_2	141	177	116	466	459	403	3.063

Bmper	5 < FC > 2	cluster_4	cluster_2	191	273	166	677	683	719	3.303
Cdk5r1	5 < FC > 2	cluster_4	cluster_2	517	505	567	1102	1168	1064	2.099
Ctgf	5 < FC > 2	cluster_4	cluster_2	643	689	623	1493	1286	1365	2.121
Ell2	5 < FC > 2	cluster_4	cluster_2	285	315	226	542	536	581	2.01
Fam102a	5 < FC > 2	cluster_4	cluster_2	1515	1937	2251	5424	5984	5855	3.025
Fhod3	5 < FC > 2	cluster_4	cluster_2	941	847	877	2053	2122	2048	2.335
Gpc1	5 < FC > 2	cluster_4	cluster_2	15633	21696	26841	37655	49599	43126	2.032
Plxnb1	5 < FC > 2	cluster_4	cluster_2	811	961	1071	1783	2577	2248	2.324
Rnd1	5 < FC > 2	cluster_4	cluster_2	40	56	54	84	114	104	2.021
Sema5a	5 < FC > 2	cluster_4	cluster_2	68	57	40	271	239	276	4.806
Sh3bp4	5 < FC > 2	cluster_4	cluster_2	865	1090	1228	2292	2619	2406	2.297

5<FC>2, H3K27me3 cluster 4, H3K4me3 cluster 3

Id	RNAseq	H3K27me3	H3K4me3	norm.dmso1	norm.dmso2	norm.dmso3	norm.epz1	norm.epz2	norm.epz3	FoldChange
Bambi	5 < FC > 2	cluster_4	cluster_3	335	227	265	831	902	798	3.066
Cspg4	5 < FC > 2	cluster_4	cluster_3	1962	3095	3862	8338	11229	9887	3.302
Eepd1	5 < FC > 2	cluster_4	cluster_3	571	590	675	1434	1590	1497	2.461
Hrc	5 < FC > 2	cluster_4	cluster_3	104	69	102	451	486	313	4.532
Hspb7	5 < FC > 2	cluster_4	cluster_3	20	37	84	141	190	164	3.473
Ibsp	5 < FC > 2	cluster_4	cluster_3	17867	15599	15329	50914	50066	49261	3.079
Insc	5 < FC > 2	cluster_4	cluster_3	521	825	809	2236	2589	2573	3.43
Jam2	5 < FC > 2	cluster_4	cluster_3	67	41	32	94	107	132	2.395
Myo1d	5 < FC > 2	cluster_4	cluster_3	3266	3446	3354	7326	7312	6985	2.148
Myrf	5 < FC > 2	cluster_4	cluster_3	31	7	33	44	115	87	3.512
Panx3	5 < FC > 2	cluster_4	cluster_3	6656	6491	5679	19668	18083	18794	3.004
Srpx2	5 < FC > 2	cluster_4	cluster_3	3143	2971	2767	6959	6245	6772	2.25
Tbc1d8	5 < FC > 2	cluster_4	cluster_3	12	15	19	63	80	70	4.613
Tmem119	5 < FC > 2	cluster_4	cluster_3	4964	6229	7776	17045	21358	19374	3.045
Tubb3	5 < FC > 2	cluster_4	cluster_3	529	637	723	2362	3033	2558	4.206

5<FC>2, H3K27me3 cluster 4, H3K4me3 cluster 4

Id	RNAseq	H3K27me3	H3K4me3	norm.dmso1	norm.dmso2	norm.dmso3	norm.epz1	norm.epz2	norm.epz3	FoldChange
5730508B09R	5 < FC > 2	cluster_4	cluster_4	43	36	34	111	100	109	2.837
Abca5	5 < FC > 2	cluster_4	cluster_4	315	215	215	593	533	521	2.216
Adam12	5 < FC > 2	cluster_4	cluster_4	208	189	224	632	664	589	3.037
Ahrr	5 < FC > 2	cluster_4	cluster_4	467	412	408	916	846	909	2.077
Alpl	5 < FC > 2	cluster_4	cluster_4	862	967	958	1930	2259	2178	2.283
Als2cl	5 < FC > 2	cluster_4	cluster_4	425	448	677	980	1248	1046	2.11
Angpt1	5 < FC > 2	cluster_4	cluster_4	43	56	30	232	199	139	4.439
Ano3	5 < FC > 2	cluster_4	cluster_4	83	138	109	406	325	277	3.048
Armcx4	5 < FC > 2	cluster_4	cluster_4	894	899	973	2598	2566	2461	2.755
Bglap	5 < FC > 2	cluster_4	cluster_4	142	179	163	582	586	476	3.392
Bglap2	5 < FC > 2	cluster_4	cluster_4	401	441	567	1232	2083	1431	3.366
C4a	5 < FC > 2	cluster_4	cluster_4	17	21	20	52	46	55	2.644
Camk2b	5 < FC > 2	cluster_4	cluster_4	263	208	239	840	885	702	3.419
Cgref1	5 < FC > 2	cluster_4	cluster_4	1520	1366	1576	4287	4680	4499	3.018
Chn1	5 < FC > 2	cluster_4	cluster_4	266	220	203	609	475	550	2.377
Cobll1	5 < FC > 2	cluster_4	cluster_4	93	156	141	267	231	287	2.008
Cpe	5 < FC > 2	cluster_4	cluster_4	4456	3789	3434	8347	7575	7763	2.029
Creb3l3	5 < FC > 2	cluster_4	cluster_4	67	120	106	381	544	404	4.519
Cys1	5 < FC > 2	cluster_4	cluster_4	8	17	16	35	42	63	3.429
Dmxl2	5 < FC > 2	cluster_4	cluster_4	200	232	176	427	455	342	2.016

Dock10	5 < FC > 2	cluster_4	cluster_4	174	198	161	475	404	374	2.353
F8a	5 < FC > 2	cluster_4	cluster_4	41	27	46	73	83	86	2.112
Fam20a	5 < FC > 2	cluster_4	cluster_4	431	588	582	1289	1389	1249	2.449
Fxyd5	5 < FC > 2	cluster_4	cluster_4	350	315	362	713	699	759	2.115
Gngt2	5 < FC > 2	cluster_4	cluster_4	12	21	16	71	83	84	4.844
Gykl1	5 < FC > 2	cluster_4	cluster_4	24	20	12	75	59	54	3.41
Hapln1	5 < FC > 2	cluster_4	cluster_4	689	685	657	1659	1610	1495	2.346
Hr	5 < FC > 2	cluster_4	cluster_4	59	106	51	148	191	261	2.777
Iqgap2	5 < FC > 2	cluster_4	cluster_4	47	46	16	84	149	100	3.095
Kcnf1	5 < FC > 2	cluster_4	cluster_4	50	45	42	112	88	128	2.39
Lrrc30	5 < FC > 2	cluster_4	cluster_4	15	9	14	71	60	51	4.842
Macc1	5 < FC > 2	cluster_4	cluster_4	16	12	20	71	45	29	3.02
Matn3	5 < FC > 2	cluster_4	cluster_4	61	71	34	171	245	192	3.68
Me2	5 < FC > 2	cluster_4	cluster_4	1683	1770	1690	3531	3380	3534	2.031
Mef2c	5 < FC > 2	cluster_4	cluster_4	2294	1970	1697	4795	4152	4920	2.328
Megf10	5 < FC > 2	cluster_4	cluster_4	150	207	139	530	569	423	3.065
Morc4	5 < FC > 2	cluster_4	cluster_4	595	588	427	1407	1356	1418	2.6
Nrip3	5 < FC > 2	cluster_4	cluster_4	9	20	11	67	42	42	3.842
Olfm2	5 < FC > 2	cluster_4	cluster_4	94	143	101	237	260	238	2.177
Pdgfa	5 < FC > 2	cluster_4	cluster_4	1812	2194	2673	6012	7480	6592	3.006
Plag1	5 < FC > 2	cluster_4	cluster_4	233	188	175	526	466	423	2.382
Ppfibp2	5 < FC > 2	cluster_4	cluster_4	274	260	256	908	754	709	3.005
Ppm1e	5 < FC > 2	cluster_4	cluster_4	156	174	112	344	343	364	2.383
Prickle1	5 < FC > 2	cluster_4	cluster_4	359	330	439	863	944	743	2.26
Prkaa2	5 < FC > 2	cluster_4	cluster_4	75	141	123	604	536	532	4.906
Proser2	5 < FC > 2	cluster_4	cluster_4	99	87	123	253	279	196	2.356
Prss50	5 < FC > 2	cluster_4	cluster_4	3	6	15	41	37	43	4.893
Ptgfrn	5 < FC > 2	cluster_4	cluster_4	1057	1113	1285	2766	3007	2867	2.499
Pth1r	5 < FC > 2	cluster_4	cluster_4	3693	5094	6068	11845	15725	14102	2.805
Ptprj	5 < FC > 2	cluster_4	cluster_4	772	972	843	2069	1632	1815	2.132
Rgcc	5 < FC > 2	cluster_4	cluster_4	43	16	29	81	98	110	3.304
Rhbdl2	5 < FC > 2	cluster_4	cluster_4	154	127	121	370	281	385	2.576
Rnf43	5 < FC > 2	cluster_4	cluster_4	466	589	686	1485	1599	1593	2.683
Robo2	5 < FC > 2	cluster_4	cluster_4	514	369	360	1695	1466	1470	3.732
Scn3a	5 < FC > 2	cluster_4	cluster_4	1477	1319	1164	3323	3054	3012	2.372
Sema6a	5 < FC > 2	cluster_4	cluster_4	581	656	645	2497	2402	2338	3.844
Sgms2	5 < FC > 2	cluster_4	cluster_4	1495	1346	1374	5005	4505	4750	3.384
Shb	5 < FC > 2	cluster_4	cluster_4	184	163	174	311	452	359	2.156
Slc16a4	5 < FC > 2	cluster_4	cluster_4	325	343	208	858	752	740	2.687
Slc22a23	5 < FC > 2	cluster_4	cluster_4	2	37	16	81	62	79	3.989
Slc40a1	5 < FC > 2	cluster_4	cluster_4	141	108	131	201	288	297	2.076
Slc7a11	5 < FC > 2	cluster_4	cluster_4	22	41	47	228	130	155	4.654
Smim5	5 < FC > 2	cluster_4	cluster_4	20	4	11	38	34	48	3.38
Sncaip	5 < FC > 2	cluster_4	cluster_4	42	24	47	201	118	152	4.154
Sorcs2	5 < FC > 2	cluster_4	cluster_4	2782	3271	4008	9352	11018	9616	2.98
Sort1	5 < FC > 2	cluster_4	cluster_4	148	193	166	368	357	427	2.274
Spns2	5 < FC > 2	cluster_4	cluster_4	404	455	445	1305	1248	1251	2.913
St3gal1	5 < FC > 2	cluster_4	cluster_4	648	579	515	1271	1138	1290	2.124
Stab1	5 < FC > 2	cluster_4	cluster_4	115	218	217	780	750	664	3.978
Ston2	5 < FC > 2	cluster_4	cluster_4	27	21	31	114	93	47	3.23

Tcf7	5 < FC > 2	cluster_4	cluster_4	1483	1429	1505	5429	5436	5587	3.724
Tmie	5 < FC > 2	cluster_4	cluster_4	195	204	257	538	562	452	2.361
Tnfrsf11b	5 < FC > 2	cluster_4	cluster_4	5064	4402	4152	17730	16027	17233	3.745
Tppp3	5 < FC > 2	cluster_4	cluster_4	25	20	104	164	266	157	3.923
Tspan7	5 < FC > 2	cluster_4	cluster_4	118	112	75	242	292	283	2.694
Xlr	5 < FC > 2	cluster_4	cluster_4	17	16	17	59	75	48	3.684

Table S3: EPZ6438-induced genes listed in figure 3E that exhibited change in gene expression (2< FC > 1.2) by RNA-seq that correlated with a reduction in H3K27me3 and presence or increase in H3K4me3 by K-means clustering.

2<FC>1.2										
**2<FC>1.2, H3K27me3 cluster 1**										
**2<FC>1.2, H3K27me3 cluster 1, H3K4me3 cluster 3**										
**Id**	**RNAseq**	**H3K27me3**	**H3K4me3**	**norm.dmso1**	**norm.dmso2**	**norm.dmso3**	**norm.epz1**	**norm.epz2**	**norm.epz3**	**FoldChange**
Adamts14	2 < FC > 1.2	cluster_1	cluster_3	334	474	601	814	963	854	1.865
Cacna1c	2 < FC > 1.2	cluster_1	cluster_3	1184	1276	1368	2071	1894	2171	1.602
Fbln7	2 < FC > 1.2	cluster_1	cluster_3	50	65	55	98	130	103	1.956
Hoxb9	2 < FC > 1.2	cluster_1	cluster_3	201	176	161	271	253	278	1.492
Ndrg4	2 < FC > 1.2	cluster_1	cluster_3	2588	2814	2704	3921	4016	3780	1.445
Nipsnap1	2 < FC > 1.2	cluster_1	cluster_3	72	72	78	131	129	113	1.679
Pax6	2 < FC > 1.2	cluster_1	cluster_3	160	194	220	365	270	291	1.61
Pdk1	2 < FC > 1.2	cluster_1	cluster_3	605	487	552	924	1015	912	1.735
Rerg	2 < FC > 1.2	cluster_1	cluster_3	1207	1029	1235	2019	2042	1961	1.735
Specc1	2 < FC > 1.2	cluster_1	cluster_3	3546	3582	3759	5522	5577	5239	1.501
**2<FC>1.2, H3K27me3 cluster 1, H3K4me3 cluster 4**										
**Id**	**RNAseq**	**H3K27me3**	**H3K4me3**	**norm.dmso1**	**norm.dmso2**	**norm.dmso3**	**norm.epz1**	**norm.epz2**	**norm.epz3**	**FoldChange**
Gli1	2 < FC > 1.2	cluster_1	cluster_4	56	116	92	117	146	90	1.339
Dab2ip	2 < FC > 1.2	cluster_1	cluster_4	1681	2201	2935	3504	4232	3767	1.687
Olfm1	2 < FC > 1.2	cluster_1	cluster_4	252	198	227	433	371	411	1.798
Svop	2 < FC > 1.2	cluster_1	cluster_4	79	80	156	177	262	190	1.986
**2<FC>1.2, H3K27me3 cluster 2**										
**2<FC>1.2, H3K27me3 cluster 2, H3K4me3 cluster 1**										
**Id**	**RNAseq**	**H3K27me3**	**H3K4me3**	**norm.dmso1**	**norm.dmso2**	**norm.dmso3**	**norm.epz1**	**norm.epz2**	**norm.epz3**	**FoldChange**
1810055G02R	2 < FC > 1.2	cluster_2	cluster_1	3010	3450	3499	4170	4684	4417	1.332
Acvr1b	2 < FC > 1.2	cluster_2	cluster_1	152	208	201	339	340	258	1.665
Akap9	2 < FC > 1.2	cluster_2	cluster_1	2916	3186	2946	4518	3937	4106	1.388
Axin2	2 < FC > 1.2	cluster_2	cluster_1	753	974	1173	1392	1805	1486	1.614
Bahd1	2 < FC > 1.2	cluster_2	cluster_1	729	1029	1252	1409	1696	1581	1.556
Chac1	2 < FC > 1.2	cluster_2	cluster_1	104	206	242	284	396	332	1.823
Dnmt3a	2 < FC > 1.2	cluster_2	cluster_1	2135	2138	2527	3466	3988	3798	1.654
Fam212b	2 < FC > 1.2	cluster_2	cluster_1	989	1039	1004	1508	1684	1507	1.55
Gars	2 < FC > 1.2	cluster_2	cluster_1	13390	12439	10929	16262	14408	15674	1.261
Hivep2	2 < FC > 1.2	cluster_2	cluster_1	404	489	413	753	730	741	1.703
Jak2	2 < FC > 1.2	cluster_2	cluster_1	3006	2605	2483	4448	3919	4066	1.536
Klf2	2 < FC > 1.2	cluster_2	cluster_1	208	203	288	391	515	450	1.938
Mpp6	2 < FC > 1.2	cluster_2	cluster_1	1604	1518	1407	2773	2471	2473	1.704
Nbas	2 < FC > 1.2	cluster_2	cluster_1	3376	3656	3577	4456	4392	4350	1.244
Pla2g12a	2 < FC > 1.2	cluster_2	cluster_1	240	196	277	378	376	422	1.649
Rbpms2	2 < FC > 1.2	cluster_2	cluster_1	61	98	79	143	149	153	1.86
Rcl1	2 < FC > 1.2	cluster_2	cluster_1	2076	1949	2103	2472	2657	2598	1.261
Reck	2 < FC > 1.2	cluster_2	cluster_1	2733	2827	2481	5341	5144	5149	1.944
Smyd5	2 < FC > 1.2	cluster_2	cluster_1	3263	3209	3378	4424	4232	4239	1.309
Tiam2	2 < FC > 1.2	cluster_2	cluster_1	3828	3994	3800	5040	5151	5157	1.321

2<FC>1.2, H3K27me3 cluster 2, H3K4me3 cluster 2

Id	RNAseq	H3K27me3	H3K4me3	norm.dmso1	norm.dmso2	norm.dmso3	norm.epz1	norm.epz2	norm.epz3	FoldChange
Acp6	2 < FC > 1.2	cluster_2	cluster_2	324	371	383	655	615	576	1.712
Adam19	2 < FC > 1.2	cluster_2	cluster_2	430	572	534	1038	965	1065	1.994
Arfgef2	2 < FC > 1.2	cluster_2	cluster_2	5282	5791	5789	7082	6849	6553	1.215
Bcl7a	2 < FC > 1.2	cluster_2	cluster_2	165	172	184	235	305	325	1.659
Cab39	2 < FC > 1.2	cluster_2	cluster_2	5449	4863	4644	6940	6326	6509	1.322
Cacna2d1	2 < FC > 1.2	cluster_2	cluster_2	931	846	765	1273	1021	1132	1.348
Ccdc134	2 < FC > 1.2	cluster_2	cluster_2	677	793	888	1034	1257	1128	1.45
Chchd7	2 < FC > 1.2	cluster_2	cluster_2	516	593	556	1011	776	952	1.645
Clint1	2 < FC > 1.2	cluster_2	cluster_2	7055	7415	7427	9664	9926	9604	1.333
Cpox	2 < FC > 1.2	cluster_2	cluster_2	1292	1126	1019	1608	1676	1622	1.428
Ddx39	2 < FC > 1.2	cluster_2	cluster_2	3664	3777	3760	4752	5141	5061	1.335
Dnmt3b	2 < FC > 1.2	cluster_2	cluster_2	254	264	263	418	397	454	1.622
Ebna1bp2	2 < FC > 1.2	cluster_2	cluster_2	2714	2553	2736	3154	3754	3462	1.296
Foxo1	2 < FC > 1.2	cluster_2	cluster_2	492	508	419	734	611	632	1.394
Gls2	2 < FC > 1.2	cluster_2	cluster_2	177	210	243	334	343	301	1.547
Nmnat2	2 < FC > 1.2	cluster_2	cluster_2	752	837	701	997	1065	976	1.328
P4ha2	2 < FC > 1.2	cluster_2	cluster_2	2369	2914	3135	4411	4758	4350	1.606
Pi4k2b	2 < FC > 1.2	cluster_2	cluster_2	3790	3071	2961	4962	4281	4241	1.373
Pkdcc	2 < FC > 1.2	cluster_2	cluster_2	370	540	702	902	1260	1038	1.983
Plrg1	2 < FC > 1.2	cluster_2	cluster_2	3871	3574	3232	4555	4444	4575	1.272
Ptar1	2 < FC > 1.2	cluster_2	cluster_2	537	489	402	758	765	828	1.648
Rrp1b	2 < FC > 1.2	cluster_2	cluster_2	1550	1716	1753	2132	2305	2163	1.315
Sh3rf1	2 < FC > 1.2	cluster_2	cluster_2	553	622	604	899	934	994	1.588
Slc36a1	2 < FC > 1.2	cluster_2	cluster_2	1150	1436	1364	1975	2085	1950	1.521
Snrpa1	2 < FC > 1.2	cluster_2	cluster_2	1615	1427	1508	2012	1914	1880	1.276
St7	2 < FC > 1.2	cluster_2	cluster_2	1824	1479	1476	2506	2027	2256	1.421
Tead2	2 < FC > 1.2	cluster_2	cluster_2	1668	1835	2212	2675	3043	3080	1.539
Tfrc	2 < FC > 1.2	cluster_2	cluster_2	2345	2120	1813	2939	2612	2927	1.351
Zbtb39	2 < FC > 1.2	cluster_2	cluster_2	787	922	917	1130	1089	1137	1.277
Zbtb7c	2 < FC > 1.2	cluster_2	cluster_2	1507	1358	1714	2063	2035	1860	1.301

2<FC>1.2, H3K27me3 cluster 2, H3K4me3 cluster 3

Id	RNAseq	H3K27me3	H3K4me3	norm.dmso1	norm.dmso2	norm.dmso3	norm.epz1	norm.epz2	norm.epz3	FoldChange
2700038G22R	2 < FC > 1.2	cluster_2	cluster_3	470	487	446	578	622	676	1.337
Casc4	2 < FC > 1.2	cluster_2	cluster_3	1306	1081	944	1808	1439	1523	1.433
Ccdc8	2 < FC > 1.2	cluster_2	cluster_3	253	407	502	495	755	714	1.688
Cdkn1a	2 < FC > 1.2	cluster_2	cluster_3	9105	8839	10000	16685	18155	17770	1.883
Cercam	2 < FC > 1.2	cluster_2	cluster_3	1098	1129	1496	1879	2363	2052	1.69
Dennd2c	2 < FC > 1.2	cluster_2	cluster_3	259	227	226	483	436	454	1.933
Ehd3	2 < FC > 1.2	cluster_2	cluster_3	1294	1533	1477	2762	2571	2566	1.834
Gorasp2	2 < FC > 1.2	cluster_2	cluster_3	8813	8916	8852	10696	10505	11103	1.215
Kdelr3	2 < FC > 1.2	cluster_2	cluster_3	8173	7100	6759	10692	9481	10109	1.375
Lrrc20	2 < FC > 1.2	cluster_2	cluster_3	759	896	927	1044	1178	1187	1.32
Mthfd2	2 < FC > 1.2	cluster_2	cluster_3	5370	5141	5112	7251	6933	6978	1.355
Pabpc4	2 < FC > 1.2	cluster_2	cluster_3	1826	2286	2839	3238	4097	3638	1.578
Ptp4a3	2 < FC > 1.2	cluster_2	cluster_3	718	1058	1224	1399	1835	1701	1.645
Shroom1	2 < FC > 1.2	cluster_2	cluster_3	60	93	84	131	171	125	1.803
Taf4b	2 < FC > 1.2	cluster_2	cluster_3	459	559	429	647	631	695	1.363
Tcn2	2 < FC > 1.2	cluster_2	cluster_3	358	412	421	714	668	646	1.703

Tspan11	2 < FC > 1.2	cluster_2	cluster_3	1067	1269	1438	1644	1800	1663	1.352
Vash1	2 < FC > 1.2	cluster_2	cluster_3	150	130	189	253	262	334	1.806
**2<FC>1.2, H3K27me3 cluster 2, H3K4me3 cluster 4**										
**Id**	**RNAseq**	**H3K27me3**	**H3K4me3**	**norm.dmso1**	**norm.dmso2**	**norm.dmso3**	**norm.epz1**	**norm.epz2**	**norm.epz3**	**FoldChange**
Arhgap29	2 < FC > 1.2	cluster_2	cluster_4	1977	1823	1653	2610	2383	2807	1.431
Frmd4a	2 < FC > 1.2	cluster_2	cluster_4	1713	1471	1673	2247	2039	2102	1.315
Klf8	2 < FC > 1.2	cluster_2	cluster_4	141	108	139	240	195	235	1.726
**2<FC>1.2, H3K27me3 cluster 3**										
**2<FC>1.2, H3K27me3 cluster 3, H3K4me3 cluster 1**										
**Id**	**RNAseq**	**H3K27me3**	**H3K4me3**	**norm.dmso1**	**norm.dmso2**	**norm.dmso3**	**norm.epz1**	**norm.epz2**	**norm.epz3**	**FoldChange**
Apba1	2 < FC > 1.2	cluster_3	cluster_1	466	485	375	685	687	783	1.626
Pla2g16	2 < FC > 1.2	cluster_3	cluster_1	5223	5075	5332	7575	7939	7616	1.48
**2<FC>1.2, H3K27me3 cluster 3, H3K4me3 cluster 2**										
**Id**	**RNAseq**	**H3K27me3**	**H3K4me3**	**norm.dmso1**	**norm.dmso2**	**norm.dmso3**	**norm.epz1**	**norm.epz2**	**norm.epz3**	**FoldChange**
Col15a1	2 < FC > 1.2	cluster_3	cluster_2	3098	3408	4205	5159	6303	5403	1.574
Gprc5c	2 < FC > 1.2	cluster_3	cluster_2	1372	1449	1531	2397	2463	2441	1.677
Hapln4	2 < FC > 1.2	cluster_3	cluster_2	1148	1096	1106	1663	1391	1397	1.329
Neurl1b	2 < FC > 1.2	cluster_3	cluster_2	271	299	304	409	414	435	1.436
Plcl1	2 < FC > 1.2	cluster_3	cluster_2	125	144	135	241	237	252	1.807
**2<FC>1.2, H3K27me3 cluster 3, H3K4me3 cluster 3**										
**Id**	**RNAseq**	**H3K27me3**	**H3K4me3**	**norm.dmso1**	**norm.dmso2**	**norm.dmso3**	**norm.epz1**	**norm.epz2**	**norm.epz3**	**FoldChange**
Cd1d1	2 < FC > 1.2	cluster_3	cluster_3	203	130	153	269	280	259	1.663
Cenpv	2 < FC > 1.2	cluster_3	cluster_3	173	159	210	241	277	337	1.576
Dennd5b	2 < FC > 1.2	cluster_3	cluster_3	506	454	466	689	730	692	1.482
Dusp23	2 < FC > 1.2	cluster_3	cluster_3	77	59	101	126	149	151	1.799
Ephb2	2 < FC > 1.2	cluster_3	cluster_3	611	740	721	1139	1502	1295	1.899
Fxyd1	2 < FC > 1.2	cluster_3	cluster_3	417	574	697	800	1031	856	1.589
Kirrel3	2 < FC > 1.2	cluster_3	cluster_3	83	76	101	159	169	191	1.987
Pdlim1	2 < FC > 1.2	cluster_3	cluster_3	948	963	1035	1763	1889	1987	1.914
Tes	2 < FC > 1.2	cluster_3	cluster_3	86	144	126	234	232	209	1.89
Wnt10a	2 < FC > 1.2	cluster_3	cluster_3	478	578	712	1073	1091	1086	1.836
**2<FC>1.2, H3K27me3 cluster 3, H3K4me3 cluster 4**										
**Id**	**RNAseq**	**H3K27me3**	**H3K4me3**	**norm.dmso1**	**norm.dmso2**	**norm.dmso3**	**norm.epz1**	**norm.epz2**	**norm.epz3**	**FoldChange**
5031414D18R	2 < FC > 1.2	cluster_3	cluster_4	74	82	77	206	121	120	1.919
Adarb1	2 < FC > 1.2	cluster_3	cluster_4	579	545	633	829	876	898	1.481
Cdk18	2 < FC > 1.2	cluster_3	cluster_4	215	292	353	491	559	465	1.76
Chchd4	2 < FC > 1.2	cluster_3	cluster_4	954	1036	958	1197	1325	1257	1.282
Des	2 < FC > 1.2	cluster_3	cluster_4	94	101	148	201	255	209	1.932
Epdr1	2 < FC > 1.2	cluster_3	cluster_4	1619	1589	1576	2449	2351	2349	1.495
Etl4	2 < FC > 1.2	cluster_3	cluster_4	4393	4854	4616	5842	5428	5889	1.238
Fam180a	2 < FC > 1.2	cluster_3	cluster_4	211	148	169	267	264	302	1.579
Iffo2	2 < FC > 1.2	cluster_3	cluster_4	1272	1283	1269	1627	1634	1642	1.282
Igf2bp2	2 < FC > 1.2	cluster_3	cluster_4	652	648	675	1203	1071	1298	1.808
Igf2r	2 < FC > 1.2	cluster_3	cluster_4	14303	15637	15194	18952	18675	18743	1.249
Larp1b	2 < FC > 1.2	cluster_3	cluster_4	221	197	190	360	371	390	1.849
Lman1	2 < FC > 1.2	cluster_3	cluster_4	8331	7875	7316	10570	9495	9925	1.275
Mreg	2 < FC > 1.2	cluster_3	cluster_4	107	96	146	225	192	194	1.744
Ncam1	2 < FC > 1.2	cluster_3	cluster_4	2140	2452	2573	2967	2989	3110	1.265
Nr4a2	2 < FC > 1.2	cluster_3	cluster_4	332	239	280	472	424	489	1.63
Ogfrl1	2 < FC > 1.2	cluster_3	cluster_4	384	322	353	453	490	561	1.421

Pag1	2 < FC > 1.2	cluster_3	cluster_4	471	442	362	599	610	569	1.397
Pde4dip	2 < FC > 1.2	cluster_3	cluster_4	3723	3820	3789	5610	5469	5657	1.477
Pgbd5	2 < FC > 1.2	cluster_3	cluster_4	100	162	119	233	253	271	1.981
Phc2	2 < FC > 1.2	cluster_3	cluster_4	1476	1992	2246	2398	2867	2771	1.406
Plce1	2 < FC > 1.2	cluster_3	cluster_4	151	101	97	233	246	197	1.945
Pmepa1	2 < FC > 1.2	cluster_3	cluster_4	734	805	914	1370	1634	1365	1.781
Ppp1r3b	2 < FC > 1.2	cluster_3	cluster_4	570	565	595	1178	1053	1124	1.939
Prr5l	2 < FC > 1.2	cluster_3	cluster_4	678	702	801	1227	1347	1298	1.775
Qsox2	2 < FC > 1.2	cluster_3	cluster_4	1865	1866	1933	2320	2408	2418	1.262
Rap1gap	2 < FC > 1.2	cluster_3	cluster_4	885	1080	1460	1491	2137	1799	1.584
Rcan1	2 < FC > 1.2	cluster_3	cluster_4	1067	1044	966	1349	1310	1290	1.284
Shc2	2 < FC > 1.2	cluster_3	cluster_4	637	796	940	1089	1236	1176	1.474
Slc7a1	2 < FC > 1.2	cluster_3	cluster_4	4482	5023	4867	6601	6708	6791	1.398
Tbc1d16	2 < FC > 1.2	cluster_3	cluster_4	718	815	906	1628	1688	1552	1.994
Tet1	2 < FC > 1.2	cluster_3	cluster_4	746	748	630	991	848	1007	1.34
Tigar	2 < FC > 1.2	cluster_3	cluster_4	335	277	272	445	451	464	1.543
Unc5b	2 < FC > 1.2	cluster_3	cluster_4	4929	6154	7221	9621	12228	10554	1.77

2<FC>1.2, H3K27me3 cluster 4

2<FC>1.2, H3K27me3 cluster 4, H3K4me3 cluster 1

Id	RNAseq	H3K27me3	H3K4me3	norm.dmso1	norm.dmso2	norm.dmso3	norm.epz1	norm.epz2	norm.epz3	FoldChange
Abl2	2 < FC > 1.2	cluster_4	cluster_1	2186	2173	2206	2715	2494	3089	1.264
Adamts12	2 < FC > 1.2	cluster_4	cluster_1	2873	2842	2569	3403	3438	3273	1.221
Arfgap1	2 < FC > 1.2	cluster_4	cluster_1	7351	8306	8960	10334	11897	11467	1.369
Arhgap24	2 < FC > 1.2	cluster_4	cluster_1	1185	1262	1185	1823	1600	1531	1.363
Bmp2k	2 < FC > 1.2	cluster_4	cluster_1	2048	1916	1793	2899	2497	2654	1.398
Ccdc6	2 < FC > 1.2	cluster_4	cluster_1	3669	3548	3338	4533	4035	4485	1.237
Cryab	2 < FC > 1.2	cluster_4	cluster_1	5649	7147	7628	8661	10213	9323	1.38
Dusp7	2 < FC > 1.2	cluster_4	cluster_1	808	1005	1265	1358	1625	1493	1.453
Dysf	2 < FC > 1.2	cluster_4	cluster_1	4044	4539	4657	6907	7356	7519	1.645
Emx2	2 < FC > 1.2	cluster_4	cluster_1	293	301	361	510	447	450	1.471
Fgfr3	2 < FC > 1.2	cluster_4	cluster_1	1578	2082	2537	3001	3815	3223	1.62
Glcci1	2 < FC > 1.2	cluster_4	cluster_1	654	642	604	928	770	902	1.368
Herpud1	2 < FC > 1.2	cluster_4	cluster_1	2659	2653	2680	3635	3630	3508	1.348
Iscu	2 < FC > 1.2	cluster_4	cluster_1	1224	1200	1271	1520	1540	1519	1.239
Klf9	2 < FC > 1.2	cluster_4	cluster_1	931	841	951	1191	1096	1244	1.296
Lrrc59	2 < FC > 1.2	cluster_4	cluster_1	14854	14266	14854	20339	21225	21003	1.423
Metrnl	2 < FC > 1.2	cluster_4	cluster_1	970	1219	1389	1745	1892	1945	1.559
Nrp2	2 < FC > 1.2	cluster_4	cluster_1	28917	29396	28511	54690	52494	53896	1.855
Phlda3	2 < FC > 1.2	cluster_4	cluster_1	3798	4316	4822	5443	5935	5162	1.278
Plaur	2 < FC > 1.2	cluster_4	cluster_1	502	568	700	686	1085	916	1.517
Prrc1	2 < FC > 1.2	cluster_4	cluster_1	3860	4197	4429	4974	5403	5345	1.259
Rreb1	2 < FC > 1.2	cluster_4	cluster_1	1187	1415	1481	1734	1875	1793	1.322
Sfr1	2 < FC > 1.2	cluster_4	cluster_1	14444	13782	13093	17216	17146	16831	1.239
Sik3	2 < FC > 1.2	cluster_4	cluster_1	4005	4296	5124	5664	5917	5941	1.305
Slc2a10	2 < FC > 1.2	cluster_4	cluster_1	2306	2177	2171	2762	2700	2847	1.249
Slc7a6	2 < FC > 1.2	cluster_4	cluster_1	5004	4934	4236	6082	5714	5840	1.244
Smg5	2 < FC > 1.2	cluster_4	cluster_1	4098	5002	5363	5958	6763	6118	1.302
Tgfb1	2 < FC > 1.2	cluster_4	cluster_1	946	1111	1296	1426	1685	1656	1.42
Tob1	2 < FC > 1.2	cluster_4	cluster_1	1950	1908	1942	2420	2402	2372	1.241
Tpst2	2 < FC > 1.2	cluster_4	cluster_1	619	763	994	967	1366	1183	1.479

Usp6nl	2 < FC > 1.2	cluster_4	cluster_1	1582	1563	1711	2381	2204	2337	1.425
Vldlr	2 < FC > 1.2	cluster_4	cluster_1	7615	6631	6091	13386	11276	11709	1.789

2<FC>1.2, H3K27me3 cluster 4, H3K4me3 cluster 2

Id	RNAseq	H3K27me3	H3K4me3	norm.dmso1	norm.dmso2	norm.dmso3	norm.epz1	norm.epz2	norm.epz3	FoldChange
Aen	2 < FC > 1.2	cluster_4	cluster_2	1611	2294	2534	2782	3571	3137	1.473
Agap1	2 < FC > 1.2	cluster_4	cluster_2	1235	1359	1504	2182	2476	2394	1.72
Amacr	2 < FC > 1.2	cluster_4	cluster_2	1112	1002	1132	2159	2104	2121	1.967
Ano6	2 < FC > 1.2	cluster_4	cluster_2	7900	8260	7578	12905	11210	11630	1.506
Ap1s2	2 < FC > 1.2	cluster_4	cluster_2	904	683	534	1357	1046	1221	1.71
Armcx3	2 < FC > 1.2	cluster_4	cluster_2	572	531	552	773	975	781	1.529
Bcorl1	2 < FC > 1.2	cluster_4	cluster_2	476	676	587	772	961	903	1.515
Bend3	2 < FC > 1.2	cluster_4	cluster_2	956	992	1060	1247	1298	1315	1.283
Bhlhe40	2 < FC > 1.2	cluster_4	cluster_2	3276	3372	3374	4617	4261	4265	1.311
Chsy1	2 < FC > 1.2	cluster_4	cluster_2	2980	3118	2902	4117	3956	4018	1.344
Col11a1	2 < FC > 1.2	cluster_4	cluster_2	26632	30592	32502	43125	50357	43714	1.529
D11Wsu47e	2 < FC > 1.2	cluster_4	cluster_2	772	709	750	1087	1112	960	1.416
Dact1	2 < FC > 1.2	cluster_4	cluster_2	1366	1283	1250	2002	1692	1784	1.405
Dot1l	2 < FC > 1.2	cluster_4	cluster_2	1352	1938	2695	2573	3569	3041	1.534
Ece1	2 < FC > 1.2	cluster_4	cluster_2	4519	5700	6348	7137	8381	7445	1.386
Eef2k	2 < FC > 1.2	cluster_4	cluster_2	3544	4150	4105	5383	6105	5761	1.462
Eif2ak3	2 < FC > 1.2	cluster_4	cluster_2	2515	2700	2837	3917	3935	3590	1.421
Eif4ebp1	2 < FC > 1.2	cluster_4	cluster_2	1358	1484	1765	1966	2453	2155	1.426
Enox1	2 < FC > 1.2	cluster_4	cluster_2	298	377	371	512	532	581	1.552
Fam131a	2 < FC > 1.2	cluster_4	cluster_2	463	633	777	948	1319	939	1.711
Farp2	2 < FC > 1.2	cluster_4	cluster_2	3224	3386	3208	4597	4334	4562	1.374
Fbxw7	2 < FC > 1.2	cluster_4	cluster_2	1099	1019	930	1485	1508	1295	1.408
Fosl2	2 < FC > 1.2	cluster_4	cluster_2	5475	5667	5492	7182	6784	6366	1.222
Gab2	2 < FC > 1.2	cluster_4	cluster_2	250	229	207	468	405	366	1.809
Gale	2 < FC > 1.2	cluster_4	cluster_2	720	1035	1418	1507	2004	1583	1.604
Gja1	2 < FC > 1.2	cluster_4	cluster_2	7714	7404	6917	9648	8848	8797	1.239
Gmppb	2 < FC > 1.2	cluster_4	cluster_2	1151	1292	1567	1816	2229	1908	1.484
Golga3	2 < FC > 1.2	cluster_4	cluster_2	4484	4980	4880	6282	6317	5806	1.283
Greb1l	2 < FC > 1.2	cluster_4	cluster_2	858	775	606	1220	1051	1243	1.57
Gsn	2 < FC > 1.2	cluster_4	cluster_2	22683	28337	34856	37685	48573	41931	1.493
Hyou1	2 < FC > 1.2	cluster_4	cluster_2	5224	5678	5420	6868	7253	6812	1.282
Ift20	2 < FC > 1.2	cluster_4	cluster_2	1450	1536	1592	1959	2062	2014	1.318
Inpp4a	2 < FC > 1.2	cluster_4	cluster_2	1641	1811	1740	2142	2233	2408	1.306
Inpp5a	2 < FC > 1.2	cluster_4	cluster_2	1446	1443	1485	2493	2475	2682	1.749
Isoc1	2 < FC > 1.2	cluster_4	cluster_2	1277	1165	1092	1476	1582	1517	1.295
Jarid2	2 < FC > 1.2	cluster_4	cluster_2	704	653	709	1009	968	916	1.401
Klhl25	2 < FC > 1.2	cluster_4	cluster_2	1063	1184	1335	1449	1892	1675	1.4
Lbh	2 < FC > 1.2	cluster_4	cluster_2	1759	1739	1931	2977	2958	3153	1.673
Lef1	2 < FC > 1.2	cluster_4	cluster_2	139	113	194	257	333	287	1.966
Lhfpl2	2 < FC > 1.2	cluster_4	cluster_2	3228	3420	3173	6181	6124	5775	1.841
Lifr	2 < FC > 1.2	cluster_4	cluster_2	6973	7062	5751	10662	8428	9529	1.446
Lrba	2 < FC > 1.2	cluster_4	cluster_2	714	701	704	1046	822	910	1.311
Lrp4	2 < FC > 1.2	cluster_4	cluster_2	8883	9507	10205	15454	16104	15706	1.653
Lrrfip1	2 < FC > 1.2	cluster_4	cluster_2	5129	4439	4576	8064	7930	7858	1.687
Mettl1	2 < FC > 1.2	cluster_4	cluster_2	994	1184	1361	1440	1663	1688	1.353
Mgat5	2 < FC > 1.2	cluster_4	cluster_2	424	435	496	805	725	723	1.661

Mon1b	2 < FC > 1.2	cluster_4	cluster_2	1360	1246	1363	1624	1729	1715	1.277
Mrpl38	2 < FC > 1.2	cluster_4	cluster_2	916	1232	1307	1551	1558	1583	1.357
Mybbp1a	2 < FC > 1.2	cluster_4	cluster_2	14121	16146	17487	20348	22085	20698	1.322
Myo19	2 < FC > 1.2	cluster_4	cluster_2	2085	2260	2461	3213	3351	2958	1.399
Nhp2	2 < FC > 1.2	cluster_4	cluster_2	2598	2926	3397	3512	4364	3843	1.313
Nr4a1	2 < FC > 1.2	cluster_4	cluster_2	814	986	1202	1500	1975	1697	1.722
Nt5e	2 < FC > 1.2	cluster_4	cluster_2	1640	1189	1315	2715	2634	2568	1.912
Nupr1	2 < FC > 1.2	cluster_4	cluster_2	1779	1726	1972	3240	3692	3324	1.873
P3h1	2 < FC > 1.2	cluster_4	cluster_2	5078	6165	6484	8393	8550	8162	1.416
Pard3b	2 < FC > 1.2	cluster_4	cluster_2	500	454	407	735	684	710	1.566
Plcl2	2 < FC > 1.2	cluster_4	cluster_2	1891	1555	1499	2509	2502	2656	1.551
Plekha1	2 < FC > 1.2	cluster_4	cluster_2	1687	1313	1466	2178	1913	2014	1.367
Rnf144b	2 < FC > 1.2	cluster_4	cluster_2	1976	2035	1793	2727	2383	2690	1.344
Sec16b	2 < FC > 1.2	cluster_4	cluster_2	4991	5085	5056	6853	6355	6132	1.278
Sec24d	2 < FC > 1.2	cluster_4	cluster_2	9162	9955	10005	12203	12166	12262	1.258
Sipa1l1	2 < FC > 1.2	cluster_4	cluster_2	2864	3068	3224	3940	3760	3592	1.233
Slit2	2 < FC > 1.2	cluster_4	cluster_2	8219	8246	7823	10471	10083	10729	1.288
Snora68	2 < FC > 1.2	cluster_4	cluster_2	160	131	135	226	248	211	1.612
Spata13	2 < FC > 1.2	cluster_4	cluster_2	1157	1324	1308	2263	2468	2374	1.875
Srm	2 < FC > 1.2	cluster_4	cluster_2	2425	3153	3607	4474	4727	4686	1.511
Srpr	2 < FC > 1.2	cluster_4	cluster_2	8000	7817	7431	9913	9250	9403	1.229
Ssfa2	2 < FC > 1.2	cluster_4	cluster_2	2829	2774	2653	3904	3889	3806	1.405
Stbd1	2 < FC > 1.2	cluster_4	cluster_2	409	438	390	667	743	683	1.693
Stk10	2 < FC > 1.2	cluster_4	cluster_2	234	316	348	416	506	487	1.566
Sun1	2 < FC > 1.2	cluster_4	cluster_2	5765	5775	6020	7401	7592	7028	1.254
Tbc1d8b	2 < FC > 1.2	cluster_4	cluster_2	3402	3372	2696	5059	4083	4530	1.444
Thbd	2 < FC > 1.2	cluster_4	cluster_2	7929	9013	9751	12359	13728	12839	1.458
Tmem43	2 < FC > 1.2	cluster_4	cluster_2	11958	12258	12333	15322	15428	14469	1.237
Trabd2b	2 < FC > 1.2	cluster_4	cluster_2	1266	1063	1153	1639	1591	1459	1.347
Trim16	2 < FC > 1.2	cluster_4	cluster_2	1707	1727	1587	2230	2213	2075	1.299
Uaca	2 < FC > 1.2	cluster_4	cluster_2	1217	1518	1434	1698	2041	1823	1.334
Ube2f	2 < FC > 1.2	cluster_4	cluster_2	1488	1363	1257	1834	1786	1763	1.311
Ube2g2	2 < FC > 1.2	cluster_4	cluster_2	2787	2961	3046	3745	4013	3704	1.303
Wisp1	2 < FC > 1.2	cluster_4	cluster_2	3763	3555	3266	5016	4640	4814	1.367
Xbp1	2 < FC > 1.2	cluster_4	cluster_2	4540	4060	4019	7352	6437	7140	1.659
Zpr1	2 < FC > 1.2	cluster_4	cluster_2	1829	1751	1944	2363	2290	2162	1.233
**2<FC>1.2, H3K27me3 cluster 4, H3K4me3 cluster 3**										
**Id**	**RNAseq**	**H3K27me3**	**H3K4me3**	**norm.dmso1**	**norm.dmso2**	**norm.dmso3**	**norm.epz1**	**norm.epz2**	**norm.epz3**	**FoldChange**
4931406P16R	2 < FC > 1.2	cluster_4	cluster_3	1855	2088	2066	2580	2618	2593	1.296
Abcb6	2 < FC > 1.2	cluster_4	cluster_3	929	1084	981	1392	1619	1409	1.476
Abhd15	2 < FC > 1.2	cluster_4	cluster_3	76	75	98	124	148	147	1.677
Amigo3	2 < FC > 1.2	cluster_4	cluster_3	128	127	125	224	208	183	1.615
Aqp1	2 < FC > 1.2	cluster_4	cluster_3	8809	8970	9494	16894	17887	17399	1.913
Banf1	2 < FC > 1.2	cluster_4	cluster_3	8063	8293	8882	9747	10981	10142	1.223
Bet1l	2 < FC > 1.2	cluster_4	cluster_3	1767	1819	2065	2306	2534	2303	1.264
Bmp4	2 < FC > 1.2	cluster_4	cluster_3	1955	2432	2618	3161	3817	3624	1.513
Btg2	2 < FC > 1.2	cluster_4	cluster_3	1267	1328	1276	1831	2015	1864	1.475
Bysl	2 < FC > 1.2	cluster_4	cluster_3	1943	2368	2448	2883	3312	2930	1.35
Ccdc112	2 < FC > 1.2	cluster_4	cluster_3	134	175	150	310	228	333	1.892
Cdc20	2 < FC > 1.2	cluster_4	cluster_3	3251	3524	3980	4441	4539	4466	1.25

Cux1	2 < FC > 1.2	cluster_4	cluster_3	2823	3131	3251	4030	4155	4137	1.338
Dlx5	2 < FC > 1.2	cluster_4	cluster_3	553	657	939	961	1394	1300	1.699
Dzip1l	2 < FC > 1.2	cluster_4	cluster_3	1339	1472	1700	1989	2011	2025	1.335
Eno3	2 < FC > 1.2	cluster_4	cluster_3	2496	2959	3318	4130	4174	3884	1.389
Fat1	2 < FC > 1.2	cluster_4	cluster_3	14429	16847	16669	20200	19666	20621	1.262
Fkbp11	2 < FC > 1.2	cluster_4	cluster_3	1425	1487	1891	2432	3085	2744	1.719
Fkbp5	2 < FC > 1.2	cluster_4	cluster_3	1474	1542	1552	1971	2106	2014	1.333
Fst	2 < FC > 1.2	cluster_4	cluster_3	5819	5675	5095	7481	6648	6982	1.273
Gps2	2 < FC > 1.2	cluster_4	cluster_3	1083	1322	1308	1524	1655	1640	1.298
Hes1	2 < FC > 1.2	cluster_4	cluster_3	52	109	83	139	158	160	1.866
Inhba	2 < FC > 1.2	cluster_4	cluster_3	1952	2064	2308	3615	3851	3745	1.772
Itga10	2 < FC > 1.2	cluster_4	cluster_3	4997	5327	5340	8372	8182	7751	1.551
Itga11	2 < FC > 1.2	cluster_4	cluster_3	3177	4067	4262	5552	6757	5974	1.589
Itpripl1	2 < FC > 1.2	cluster_4	cluster_3	787	799	818	1077	1115	1064	1.354
Kctd14	2 < FC > 1.2	cluster_4	cluster_3	311	291	337	428	525	498	1.546
Klf4	2 < FC > 1.2	cluster_4	cluster_3	3643	3634	3829	6713	7169	7222	1.9
Lyn	2 < FC > 1.2	cluster_4	cluster_3	1383	1291	1131	1854	1788	1855	1.445
Map6	2 < FC > 1.2	cluster_4	cluster_3	982	1048	1106	1399	1535	1474	1.405
Mapk12	2 < FC > 1.2	cluster_4	cluster_3	900	775	885	1178	1183	1106	1.354
Mef2d	2 < FC > 1.2	cluster_4	cluster_3	1011	1274	1491	1516	1863	1934	1.406
Mir17hg	2 < FC > 1.2	cluster_4	cluster_3	165	110	151	247	266	209	1.701
Mybl2	2 < FC > 1.2	cluster_4	cluster_3	546	596	712	826	1123	939	1.557
Nav1	2 < FC > 1.2	cluster_4	cluster_3	1779	1790	2052	3681	3339	3383	1.85
Pcolce2	2 < FC > 1.2	cluster_4	cluster_3	1587	1603	1769	2003	2126	2237	1.283
Pfkfb4	2 < FC > 1.2	cluster_4	cluster_3	774	894	953	1345	1472	1386	1.602
Pif1	2 < FC > 1.2	cluster_4	cluster_3	687	924	902	1119	1422	1258	1.511
Plp2	2 < FC > 1.2	cluster_4	cluster_3	2281	2516	2381	3228	2990	3091	1.297
Pogk	2 < FC > 1.2	cluster_4	cluster_3	1330	1391	1423	1829	1777	1837	1.313
Ptgs1	2 < FC > 1.2	cluster_4	cluster_3	3503	3425	3210	4804	4220	4321	1.316
Ptpn4	2 < FC > 1.2	cluster_4	cluster_3	836	846	677	1544	1329	1432	1.825
Pus1	2 < FC > 1.2	cluster_4	cluster_3	910	1149	1339	1555	1812	1567	1.451
Pycr1	2 < FC > 1.2	cluster_4	cluster_3	743	926	1130	1414	1888	1489	1.711
Pygm	2 < FC > 1.2	cluster_4	cluster_3	133	76	131	177	217	183	1.699
Rasl11a	2 < FC > 1.2	cluster_4	cluster_3	359	404	369	618	652	628	1.676
Rcc1	2 < FC > 1.2	cluster_4	cluster_3	2202	2447	2617	2980	3477	3164	1.324
Runx3	2 < FC > 1.2	cluster_4	cluster_3	1365	1448	1357	2040	2086	2248	1.528
Secisbp2	2 < FC > 1.2	cluster_4	cluster_3	681	703	846	945	1061	943	1.321
Sgca	2 < FC > 1.2	cluster_4	cluster_3	276	259	288	379	476	425	1.555
Slc6a9	2 < FC > 1.2	cluster_4	cluster_3	849	1001	1145	1233	1419	1349	1.335
Smpdl3b	2 < FC > 1.2	cluster_4	cluster_3	1517	1669	1632	2351	2616	2532	1.556
Snhg18	2 < FC > 1.2	cluster_4	cluster_3	3181	2946	2815	4368	3710	4020	1.353
Snhg7	2 < FC > 1.2	cluster_4	cluster_3	598	558	532	740	774	787	1.364
Snta1	2 < FC > 1.2	cluster_4	cluster_3	1096	1678	1972	2426	3419	2977	1.858
Sp7	2 < FC > 1.2	cluster_4	cluster_3	4456	4943	5401	8898	10374	9465	1.942
Surf4	2 < FC > 1.2	cluster_4	cluster_3	22664	23738	24357	28870	28497	27955	1.206
Thg1l	2 < FC > 1.2	cluster_4	cluster_3	547	582	612	789	817	819	1.392
Tmem214	2 < FC > 1.2	cluster_4	cluster_3	4263	5308	6180	6890	8203	7544	1.437
Tmem97	2 < FC > 1.2	cluster_4	cluster_3	3555	3217	3292	4928	4754	4532	1.413
Trmt61a	2 < FC > 1.2	cluster_4	cluster_3	438	558	720	724	1020	833	1.501
Wnt10b	2 < FC > 1.2	cluster_4	cluster_3	902	964	1060	1462	1595	1493	1.554

Xaf1	2 < FC > 1.2	cluster_4	cluster_3	815	981	738	1706	1605	1635	1.952
Zbtb18	2 < FC > 1.2	cluster_4	cluster_3	595	588	588	904	894	874	1.509
Zfp593	2 < FC > 1.2	cluster_4	cluster_3	225	285	283	336	516	491	1.693
**2<FC>1.2, H3K27me3 cluster 4, H3K4me3 cluster 4**										
**Id**	**RNAseq**	**H3K27me3**	**H3K4me3**	**norm.dmso1**	**norm.dmso2**	**norm.dmso3**	**norm.epz1**	**norm.epz2**	**norm.epz3**	**FoldChange**
2010204K13R	2 < FC > 1.2	cluster_4	cluster_4	79	104	102	146	184	152	1.686
2900026A02R	2 < FC > 1.2	cluster_4	cluster_4	2785	3058	3053	3381	3890	3742	1.238
3830403N18R	2 < FC > 1.2	cluster_4	cluster_4	79	110	78	213	118	175	1.898
Aaas	2 < FC > 1.2	cluster_4	cluster_4	3041	3030	3567	3943	4125	4098	1.262
Abr	2 < FC > 1.2	cluster_4	cluster_4	3714	4070	4166	5123	4981	5336	1.292
Akap6	2 < FC > 1.2	cluster_4	cluster_4	1206	1316	1063	1917	1827	1829	1.555
Akirin1	2 < FC > 1.2	cluster_4	cluster_4	1946	1838	1682	2391	2117	2448	1.273
Aldh1l2	2 < FC > 1.2	cluster_4	cluster_4	5042	4687	4399	7202	6432	6519	1.427
Angptl2	2 < FC > 1.2	cluster_4	cluster_4	4485	5010	5449	7166	7444	6897	1.439
Ank2	2 < FC > 1.2	cluster_4	cluster_4	958	898	874	1309	1208	1425	1.444
Anxa8	2 < FC > 1.2	cluster_4	cluster_4	2072	1818	1910	2445	2736	2480	1.321
Arfgap3	2 < FC > 1.2	cluster_4	cluster_4	3702	4012	4068	4997	5263	4769	1.276
Arhgap32	2 < FC > 1.2	cluster_4	cluster_4	637	643	555	1008	831	920	1.504
Arhgap44	2 < FC > 1.2	cluster_4	cluster_4	226	249	298	491	451	450	1.798
Arhgef28	2 < FC > 1.2	cluster_4	cluster_4	891	827	921	1128	1107	1134	1.277
Arl4a	2 < FC > 1.2	cluster_4	cluster_4	157	90	126	192	222	235	1.745
Armcx6	2 < FC > 1.2	cluster_4	cluster_4	144	112	120	192	255	292	1.97
Asns	2 < FC > 1.2	cluster_4	cluster_4	6044	5520	5030	8924	7816	8696	1.533
Atp1a1	2 < FC > 1.2	cluster_4	cluster_4	8063	9319	9880	12884	13566	12918	1.444
Atp2b4	2 < FC > 1.2	cluster_4	cluster_4	2692	2665	2862	4965	4781	5070	1.802
Aven	2 < FC > 1.2	cluster_4	cluster_4	568	618	605	925	813	708	1.365
BC017158	2 < FC > 1.2	cluster_4	cluster_4	493	705	780	830	1042	1099	1.5
Bcat1	2 < FC > 1.2	cluster_4	cluster_4	5653	5621	5277	7142	6639	6827	1.245
Blzf1	2 < FC > 1.2	cluster_4	cluster_4	1845	1604	1440	2256	1880	2312	1.319
Bmp1	2 < FC > 1.2	cluster_4	cluster_4	3354	4297	5515	6345	7562	7040	1.591
Camk2a	2 < FC > 1.2	cluster_4	cluster_4	259	317	298	499	492	410	1.601
Capn6	2 < FC > 1.2	cluster_4	cluster_4	16023	12019	11324	19664	17001	17731	1.382
Car13	2 < FC > 1.2	cluster_4	cluster_4	1304	1134	1079	1527	1501	1643	1.329
Ccnd1	2 < FC > 1.2	cluster_4	cluster_4	15285	16978	17886	23546	23755	24115	1.424
Cd276	2 < FC > 1.2	cluster_4	cluster_4	3087	3387	3572	4270	4492	4132	1.283
Cdc42ep3	2 < FC > 1.2	cluster_4	cluster_4	4774	4071	3851	6770	6350	6477	1.544
Cdh2	2 < FC > 1.2	cluster_4	cluster_4	5562	5248	4836	6442	6561	6148	1.224
Cdr2	2 < FC > 1.2	cluster_4	cluster_4	2403	2136	2082	3533	3387	3392	1.558
Cemip	2 < FC > 1.2	cluster_4	cluster_4	8388	8104	7541	11102	9487	9866	1.267
Cerk	2 < FC > 1.2	cluster_4	cluster_4	1003	1128	1120	1411	1458	1498	1.343
Cfh	2 < FC > 1.2	cluster_4	cluster_4	2249	1907	1541	3308	2721	2922	1.572
Chst15	2 < FC > 1.2	cluster_4	cluster_4	3977	4162	3670	5503	4839	4882	1.289
Cmtm4	2 < FC > 1.2	cluster_4	cluster_4	8231	7524	7600	10191	8835	9784	1.234
Cnppd1	2 < FC > 1.2	cluster_4	cluster_4	1926	2016	2358	2776	2724	2612	1.287
Cthrc1	2 < FC > 1.2	cluster_4	cluster_4	547	455	466	786	749	811	1.6
Cxadr	2 < FC > 1.2	cluster_4	cluster_4	185	147	157	350	278	241	1.781
D1Ertd622e	2 < FC > 1.2	cluster_4	cluster_4	312	302	312	510	504	578	1.718
Ddit4l	2 < FC > 1.2	cluster_4	cluster_4	714	633	541	1052	925	803	1.474
Dgat2	2 < FC > 1.2	cluster_4	cluster_4	75	92	100	141	221	138	1.873
Dhx32	2 < FC > 1.2	cluster_4	cluster_4	905	919	942	1106	1210	1231	1.283

Dlc1	2 < FC > 1.2	cluster_4	cluster_4	3811	4268	4132	5522	5622	5412	1.356
Dtx4	2 < FC > 1.2	cluster_4	cluster_4	650	678	645	1165	1184	1108	1.752
Dusp8	2 < FC > 1.2	cluster_4	cluster_4	501	407	422	912	806	798	1.894
Dyrk3	2 < FC > 1.2	cluster_4	cluster_4	73	96	86	153	163	162	1.872
Fam101b	2 < FC > 1.2	cluster_4	cluster_4	2729	2705	2780	4855	4687	5162	1.79
Fam189a2	2 < FC > 1.2	cluster_4	cluster_4	940	1071	1055	1263	1426	1324	1.308
Fam198b	2 < FC > 1.2	cluster_4	cluster_4	682	541	523	1281	893	1023	1.832
Fam3c	2 < FC > 1.2	cluster_4	cluster_4	4259	3650	3443	5614	5076	5567	1.432
Fam46a	2 < FC > 1.2	cluster_4	cluster_4	2092	1741	1667	3306	2751	3155	1.675
Fign	2 < FC > 1.2	cluster_4	cluster_4	250	159	257	405	326	412	1.714
Fmnl3	2 < FC > 1.2	cluster_4	cluster_4	3752	4249	4399	6344	7134	6381	1.601
Frs2	2 < FC > 1.2	cluster_4	cluster_4	3701	3734	3263	5152	4335	5014	1.356
Fzd5	2 < FC > 1.2	cluster_4	cluster_4	1116	1219	1180	1930	1876	2019	1.656
Garem	2 < FC > 1.2	cluster_4	cluster_4	159	186	182	302	266	262	1.575
Gnb4	2 < FC > 1.2	cluster_4	cluster_4	462	421	382	604	593	570	1.398
Gne	2 < FC > 1.2	cluster_4	cluster_4	2072	2357	2779	3413	4032	3801	1.559
Golim4	2 < FC > 1.2	cluster_4	cluster_4	5578	5267	5096	6949	6600	6022	1.228
Gper1	2 < FC > 1.2	cluster_4	cluster_4	516	594	588	880	1067	841	1.642
Gpr153	2 < FC > 1.2	cluster_4	cluster_4	1614	1811	1998	2751	3089	2757	1.585
Gpr180	2 < FC > 1.2	cluster_4	cluster_4	1466	1638	1792	1982	2143	2173	1.286
Gprc5b	2 < FC > 1.2	cluster_4	cluster_4	1321	1459	1304	2431	2153	2391	1.708
Gpsm2	2 < FC > 1.2	cluster_4	cluster_4	5713	5133	4627	7598	6618	7004	1.372
Grb14	2 < FC > 1.2	cluster_4	cluster_4	215	198	176	359	283	337	1.664
Gtf2ird1	2 < FC > 1.2	cluster_4	cluster_4	1658	1775	1863	2601	2933	2529	1.522
Hip1	2 < FC > 1.2	cluster_4	cluster_4	5571	6600	6343	7680	8000	7516	1.253
Hipk2	2 < FC > 1.2	cluster_4	cluster_4	484	552	584	907	764	871	1.568
Hk2	2 < FC > 1.2	cluster_4	cluster_4	1642	1801	1828	2328	2419	2444	1.364
Hmcn1	2 < FC > 1.2	cluster_4	cluster_4	198	134	141	356	258	249	1.831
Hspa4l	2 < FC > 1.2	cluster_4	cluster_4	260	250	192	457	397	364	1.737
Htra3	2 < FC > 1.2	cluster_4	cluster_4	203	152	247	334	333	384	1.743
Impdh1	2 < FC > 1.2	cluster_4	cluster_4	2623	3233	4235	4288	5821	5052	1.502
Jade1	2 < FC > 1.2	cluster_4	cluster_4	522	580	461	647	833	673	1.378
Jph2	2 < FC > 1.2	cluster_4	cluster_4	2253	3050	3215	3800	4372	4089	1.439
Kcnk2	2 < FC > 1.2	cluster_4	cluster_4	204	176	190	355	299	345	1.752
Kif21a	2 < FC > 1.2	cluster_4	cluster_4	1023	953	737	1298	1279	1100	1.357
Klf10	2 < FC > 1.2	cluster_4	cluster_4	689	645	731	963	970	995	1.417
Klf13	2 < FC > 1.2	cluster_4	cluster_4	2611	2752	2908	3310	3521	3352	1.231
Klrg2	2 < FC > 1.2	cluster_4	cluster_4	137	150	166	300	267	261	1.825
Kremen1	2 < FC > 1.2	cluster_4	cluster_4	5866	7088	7701	9282	9610	9017	1.351
Ldlrap1	2 < FC > 1.2	cluster_4	cluster_4	1457	1873	2051	2460	3062	3019	1.587
Lgalsl	2 < FC > 1.2	cluster_4	cluster_4	3109	2914	2507	3992	3421	3713	1.305
Lox	2 < FC > 1.2	cluster_4	cluster_4	19208	17680	15495	26760	22722	24502	1.412
Lrig1	2 < FC > 1.2	cluster_4	cluster_4	635	734	762	1357	1320	1308	1.869
Lrrc1	2 < FC > 1.2	cluster_4	cluster_4	218	240	240	419	355	350	1.61
Lrrc8b	2 < FC > 1.2	cluster_4	cluster_4	128	162	152	212	240	212	1.5
Ltv1	2 < FC > 1.2	cluster_4	cluster_4	3781	3245	2902	4548	4347	4027	1.302
Maged2	2 < FC > 1.2	cluster_4	cluster_4	7172	8426	9896	9948	12608	11297	1.328
Maml3	2 < FC > 1.2	cluster_4	cluster_4	342	442	516	640	705	666	1.546
Map1b	2 < FC > 1.2	cluster_4	cluster_4	9570	9403	8727	16816	14038	16191	1.698
Map3k1	2 < FC > 1.2	cluster_4	cluster_4	120	152	150	249	241	264	1.785

Mapk6	2 < FC > 1.2	cluster_4	cluster_4	2810	2898	2621	3680	3226	3593	1.26
Mb21d2	2 < FC > 1.2	cluster_4	cluster_4	251	242	263	411	436	424	1.682
Me1	2 < FC > 1.2	cluster_4	cluster_4	3496	3336	2732	4636	4149	4441	1.383
Mical3	2 < FC > 1.2	cluster_4	cluster_4	2204	2671	3046	4041	4591	4128	1.611
Mpzl1	2 < FC > 1.2	cluster_4	cluster_4	2319	2066	2076	2635	2757	2631	1.242
Mtmr10	2 < FC > 1.2	cluster_4	cluster_4	1589	1523	1539	2009	1820	1927	1.238
Mtus1	2 < FC > 1.2	cluster_4	cluster_4	2196	2083	1830	2885	2671	2642	1.342
Net1	2 < FC > 1.2	cluster_4	cluster_4	820	774	859	1324	1481	1448	1.734
Nfil3	2 < FC > 1.2	cluster_4	cluster_4	956	1053	1091	1343	1367	1241	1.274
Nkd2	2 < FC > 1.2	cluster_4	cluster_4	313	247	318	518	519	396	1.632
Nrbf2	2 < FC > 1.2	cluster_4	cluster_4	709	726	643	982	909	819	1.305
Nsf	2 < FC > 1.2	cluster_4	cluster_4	4519	4637	4546	5738	5698	5573	1.241
Nuak1	2 < FC > 1.2	cluster_4	cluster_4	4595	4769	4763	8691	8912	9134	1.892
Olfml1	2 < FC > 1.2	cluster_4	cluster_4	137	134	148	304	235	207	1.776
Optn	2 < FC > 1.2	cluster_4	cluster_4	1720	1748	1690	2192	2310	2222	1.304
Pald1	2 < FC > 1.2	cluster_4	cluster_4	1391	1746	2012	2902	3362	2924	1.784
Papss2	2 < FC > 1.2	cluster_4	cluster_4	4033	3692	3574	6414	5711	5925	1.598
Pdia5	2 < FC > 1.2	cluster_4	cluster_4	1693	1842	1937	3016	2885	2867	1.601
Pdzd4	2 < FC > 1.2	cluster_4	cluster_4	1218	1222	1521	1723	1868	1827	1.367
Pfkfb3	2 < FC > 1.2	cluster_4	cluster_4	1007	1069	1046	1567	1504	1519	1.47
Plcd1	2 < FC > 1.2	cluster_4	cluster_4	1442	1982	2332	3272	4240	3472	1.907
Polr3d	2 < FC > 1.2	cluster_4	cluster_4	879	776	1039	1182	1479	1292	1.468
Prdx6	2 < FC > 1.2	cluster_4	cluster_4	4448	4287	4327	5450	5228	5288	1.222
Ptch1	2 < FC > 1.2	cluster_4	cluster_4	542	570	680	1125	1141	1025	1.835
Ptgfr	2 < FC > 1.2	cluster_4	cluster_4	6689	6015	5192	9118	7477	8490	1.402
Ptk2b	2 < FC > 1.2	cluster_4	cluster_4	389	484	414	635	626	597	1.442
Ptprm	2 < FC > 1.2	cluster_4	cluster_4	2352	2567	2599	3356	3155	3383	1.316
Pxdc1	2 < FC > 1.2	cluster_4	cluster_4	885	984	970	1637	1745	1629	1.765
Pxylp1	2 < FC > 1.2	cluster_4	cluster_4	1668	1774	1690	2889	2966	2884	1.703
Rabgap1l	2 < FC > 1.2	cluster_4	cluster_4	1107	972	981	1477	1294	1389	1.36
Raph1	2 < FC > 1.2	cluster_4	cluster_4	4558	4091	3590	7948	6678	7624	1.818
Rasa3	2 < FC > 1.2	cluster_4	cluster_4	3730	3919	4020	5897	6652	6084	1.597
Rassf3	2 < FC > 1.2	cluster_4	cluster_4	1594	1544	1551	2195	2114	2328	1.415
Reps2	2 < FC > 1.2	cluster_4	cluster_4	359	339	288	616	531	617	1.792
Rftn2	2 < FC > 1.2	cluster_4	cluster_4	2295	2226	2222	3018	3073	2747	1.311
Rfx3	2 < FC > 1.2	cluster_4	cluster_4	429	431	382	623	564	553	1.402
Rgl1	2 < FC > 1.2	cluster_4	cluster_4	823	877	816	1339	1255	1275	1.538
Rras2	2 < FC > 1.2	cluster_4	cluster_4	2190	1850	1628	3262	2821	3440	1.681
Rrbp1	2 < FC > 1.2	cluster_4	cluster_4	21350	26403	30205	32133	39325	34855	1.364
Runx1	2 < FC > 1.2	cluster_4	cluster_4	446	439	478	666	571	656	1.387
Runx2	2 < FC > 1.2	cluster_4	cluster_4	11267	10994	10500	17250	15631	17078	1.525
Scara3	2 < FC > 1.2	cluster_4	cluster_4	6451	6817	6991	8459	8482	8318	1.247
Sdc4	2 < FC > 1.2	cluster_4	cluster_4	4566	5163	4851	6128	6478	6428	1.305
Sec13	2 < FC > 1.2	cluster_4	cluster_4	14044	15615	17466	19412	22284	19999	1.309
Sec24a	2 < FC > 1.2	cluster_4	cluster_4	2509	2481	2077	3189	2921	3123	1.307
Sec31a	2 < FC > 1.2	cluster_4	cluster_4	17881	19468	19218	23177	23454	22193	1.217
Sema3e	2 < FC > 1.2	cluster_4	cluster_4	240	253	194	355	325	311	1.444
Sema4b	2 < FC > 1.2	cluster_4	cluster_4	605	736	784	953	1132	1096	1.496
Serpine1	2 < FC > 1.2	cluster_4	cluster_4	456	486	510	671	703	776	1.48
Sertad4	2 < FC > 1.2	cluster_4	cluster_4	1579	1448	1463	2419	2260	2313	1.558

Sipa1l2	2 < FC > 1.2	cluster_4	cluster_4	260	263	295	535	496	487	1.854
Sipa1l3	2 < FC > 1.2	cluster_4	cluster_4	1178	1882	2353	2459	3140	2885	1.567
Slc17a9	2 < FC > 1.2	cluster_4	cluster_4	95	129	102	189	226	142	1.707
Slc1a4	2 < FC > 1.2	cluster_4	cluster_4	4359	4791	4422	5913	5748	5997	1.301
Slc22a15	2 < FC > 1.2	cluster_4	cluster_4	200	250	236	302	414	407	1.634
Slc25a5	2 < FC > 1.2	cluster_4	cluster_4	10976	11356	12052	14068	14117	13762	1.22
Slc39a11	2 < FC > 1.2	cluster_4	cluster_4	1246	1262	1539	1665	1946	1749	1.324
Slc39a13	2 < FC > 1.2	cluster_4	cluster_4	2556	3487	4026	4606	5341	4778	1.462
Slc39a14	2 < FC > 1.2	cluster_4	cluster_4	2505	2333	2386	3108	2932	3082	1.263
Slc41a2	2 < FC > 1.2	cluster_4	cluster_4	2232	2220	1985	3442	3021	3309	1.518
Slc7a6os	2 < FC > 1.2	cluster_4	cluster_4	1414	1374	1330	1783	1692	1688	1.254
Smad1	2 < FC > 1.2	cluster_4	cluster_4	1066	1038	1038	1403	1349	1581	1.379
Smad3	2 < FC > 1.2	cluster_4	cluster_4	3808	4339	3873	5198	4736	5052	1.247
Smarca1	2 < FC > 1.2	cluster_4	cluster_4	278	302	251	497	533	486	1.824
Smco4	2 < FC > 1.2	cluster_4	cluster_4	125	102	110	194	224	194	1.82
Sox6	2 < FC > 1.2	cluster_4	cluster_4	2854	2998	2655	3763	3481	3804	1.299
Sparc	2 < FC > 1.2	cluster_4	cluster_4	246807	250707	253390	338559	339532	329220	1.341
Spef1	2 < FC > 1.2	cluster_4	cluster_4	183	188	169	257	256	304	1.512
Spen	2 < FC > 1.2	cluster_4	cluster_4	1377	1798	2244	2296	2775	2544	1.404
Spock2	2 < FC > 1.2	cluster_4	cluster_4	1715	2147	2148	2451	2838	2882	1.359
Spry1	2 < FC > 1.2	cluster_4	cluster_4	243	256	251	507	483	428	1.889
St6galnac6	2 < FC > 1.2	cluster_4	cluster_4	4660	5202	5686	6162	7014	6441	1.261
Stk39	2 < FC > 1.2	cluster_4	cluster_4	1394	1328	1111	2314	2172	2419	1.802
Tbrg1	2 < FC > 1.2	cluster_4	cluster_4	14054	13093	12983	17703	17189	17158	1.297
Tead4	2 < FC > 1.2	cluster_4	cluster_4	253	217	303	354	408	402	1.504
Tgfbi	2 < FC > 1.2	cluster_4	cluster_4	740	763	652	1393	1206	1302	1.811
Tle1	2 < FC > 1.2	cluster_4	cluster_4	584	688	627	870	929	856	1.398
Tle4	2 < FC > 1.2	cluster_4	cluster_4	326	342	344	526	499	424	1.431
Tll1	2 < FC > 1.2	cluster_4	cluster_4	527	555	564	1042	897	870	1.706
Tnc	2 < FC > 1.2	cluster_4	cluster_4	36676	39977	40793	73198	75957	72996	1.892
Tnfaip2	2 < FC > 1.2	cluster_4	cluster_4	1880	2148	2407	2678	2972	2890	1.327
Tnfrsf19	2 < FC > 1.2	cluster_4	cluster_4	9466	8591	8722	16899	17347	17584	1.936
Tnfrsf23	2 < FC > 1.2	cluster_4	cluster_4	631	428	374	914	696	785	1.674
Tnn	2 < FC > 1.2	cluster_4	cluster_4	1319	1408	1443	1862	1951	1985	1.39
Trpm4	2 < FC > 1.2	cluster_4	cluster_4	600	863	1092	1174	1534	1338	1.583
Trpv4	2 < FC > 1.2	cluster_4	cluster_4	503	691	897	811	1304	1256	1.611
Ttll7	2 < FC > 1.2	cluster_4	cluster_4	553	507	391	849	640	743	1.539
Vav2	2 < FC > 1.2	cluster_4	cluster_4	1071	1242	1326	1984	2167	2153	1.731
Vegfa	2 < FC > 1.2	cluster_4	cluster_4	945	1082	1152	1542	1473	1551	1.435
Vps37b	2 < FC > 1.2	cluster_4	cluster_4	191	336	368	450	606	488	1.723
Xylb	2 < FC > 1.2	cluster_4	cluster_4	244	251	266	373	383	490	1.633
Zbtb40	2 < FC > 1.2	cluster_4	cluster_4	749	1012	1101	1208	1460	1374	1.411
Znrf3	2 < FC > 1.2	cluster_4	cluster_4	1081	1064	1080	1490	1303	1485	1.327
Zswim6	2 < FC > 1.2	cluster_4	cluster_4	1030	1184	970	1361	1376	1505	1.332

Table S4: List of primers used in this study.

Gene	Forward	Reverse
Gapdh	CATCACTGCCACCCAGAAGACTG	ATGCCAGTGAGCTTCCCGTTCAG
Alpl	CCAGAAAGACACCTTGACTGTGG	TCTTGTCCGTGTCGCTCACCAT
Bglap	GCAATAAGGTAGTGAACAGACTCC	CCATAGATGCGTTTGTAGGCGG
Runx2	TCTGGAAAAAAGGAGGGACTATG	GGTGCTCGGATCCCAAAAGAA
Ibsp	GAATGGCCTGTGCTTTCTCG	CCGGTACTTAAAGACCCCGTT
Phex	CAACGTTCCGCGGTCAATAC	GTGTTGCTTGGTCCAGCTTC
Phospho	ATGAGCGGGTGTTTTCCAG	ATCGAAGTCGAAGGTGAGGAG
Gli1	GGAAGTCCTATTCACGCCTTG	CAACCTTCTTGCTCACACATGTAA
Gli2	TACCTCAACCCTGTGGATGC	CTACCAGCGAGTTGGGAGAG
Intergenic	ATTTTGTGCTGCATAACCTCCT	TAGCAACATCCTAAGCTGGACA
Actb	CGTATTAGGTCCATCTTGAGAGTAC	GCCACGATTATTGTAGGCGTGATCGTAGC
Neuro1	TGGTCTCCTGAGTGATGTCG	GCCGTACTTAAGGGGTCCTG

Table S5: List of antibodies used in this study.

Antibody	Company	CAS
H3K27me3	Cell Sig	9733S
Laminin B	Santa Cruz	sc-6216
Ezh2	Cell Signaling	5246s
Total H3	Millipore	05-928
Runx2	MBL International	8G5
Ezh1	Abcam	AB176115
Suz12	Cell Signaling	3737s
H3K27Ac	Millipore	07-360
EDD	Cell Signaling	516731
H3K4me3	Cell Signaling	9751S
H3K9me3	Cell Signaling	139695
GAPDH	Cell Signaling	51745
B-catenin	Cell Signaling	8814
Rabbit IgG	Cell Signaling	7074S
mIgG BP-HRP	Santa Cruz	sc-516102
